# The genetic overlap between major depressive disorder, white blood cell counts and interleukin 6

**DOI:** 10.1016/j.jadr.2025.100889

**Published:** 2025-02-18

**Authors:** Erik D Wiström, Kevin S O’Connell, Elise Koch, Piotr Jaholkowski, Guy F.L. Hindley, Nils Eiel Steen, Pravesh Parekh, Charge Consortium, Oleksandr Frei, Nadine Parker, Alexey Shadrin, Srdjan Djurovic, Anders Dale, Ole A Andreassen, Olav B Smeland

**Affiliations:** a Centre for Precision Psychiatry, Institute of Clinical Medicine, University of Oslo, Oslo, Norway, Division of Mental Health and Addiction, Oslo University Hospital, Oslo, Norway; b Department of Medical Genetics, Oslo University Hospital, Oslo, Norway, Department of Clinical Science, University of Bergen, Bergen, Norway; c Department of Radiology, School of Medicine, University of California San Diego, La Jolla, California, USA, Center for Multimodal Imaging and Genetics, University of California San Diego, La Jolla, California, USA; Department of Cognitive Science, University of California San Diego, La Jolla, California, USA; Department of Psychiatry, University of California San Diego, La Jolla, California, USA; Department of Neuroscience, University of California San Diego, La Jolla, California, USA

**Keywords:** GWAS, Depressive etiology, Leukocyte, Interleukin 6, MoBa

## Abstract

**Background:**

Immune dysregulation may contribute to the pathophysiology of major depressive disorder (MDD). Here we aimed to identify genetic architecture jointly associated with MDD, white blood cell (WBC) count and interleukin 6 (IL-6) levels.

**Methods:**

Using genome-wide association studies summary statistics on MDD (330,173 cases and 727,595 controls), WBC counts (*n*_*max*_ = 563,946) and IL-6 (*n* = 52,654), we performed linkage disequilibrium (LD) score regression, bivariate causal mixture model (MiXeR), conjunctional false discovery rate (conjFDR) and Mendelian randomization (MR) analyses. Additionally, we used an independent MDD dataset (9,582 cases and 84,670 controls) from the Norwegian Mother, Father and Child Cohort Study for polygenic risk score (PRS) analyses.

**Findings:**

We found a significant positive genetic correlation (rg = 0.22) between MDD and IL-6. MiXeR estimates indicated substantial differences in the polygenicity of MDD (13.7K variants), WBC subgroups (0.8K-1.8K variants), and IL-6 (0.2K variants), with 10.1 %-31.4 % of the variants influencing WBC subgroups overlapping with MDD. We identified MDD risk loci shared with basophils (8), eosinophils (17), lymphocytes (23), monocytes (14), neutrophils (20), and total WBC counts (20), as well as two loci shared between MDD and IL-6, at conjFDR *<*0.05. PRS analysis showed a weak, but significantly increased risk for MDD dependent on monocyte count.

**Limitations:**

The analyses only included European ancestry samples, and the causal genes associated with the identified genetic loci were not experimentally validated.

**Conclusions:**

MDD shares genetic underpinnings with immune system components, which implicates immune- mediated pathways in the pathophysiology of MDD. However, this connection may only be relevant for a minority of patients.

## Introduction

Major depressive disorder (MDD) is a common mental illness, accompanied by considerable morbidity and mortality (James et al., 2018; Mann et al., 2005). While the pathophysiology of MDD is mainly unclear (Cui et al., 2024), growing evidence suggests immune dysregulation as a contributing factor in at least a subgroup of patients (Beurel et al., 2020; Gibney and Drexhage, 2013; Grosse et al., 2015; Medina-Rodriguez et al., 2018). A better understanding of the role of immune dysregulation in MDD might inform the development of new treatment targets and options.

White blood cells (WBCs) play a pivotal role in both adaptive and innate immune responses (Flannagan et al., 2012; Goodnow et al., 2010; Masopust and Schenkel, 2013). WBC count is shown to be increased in a subgroup of patients with MDD and has been suggested to be a part of the etiology of the disorder (Beurel et al., 2020; Miller et al., 2009; Miller and Raison, 2016). Interleukin 6 (IL-6) is a pleiotropic cytokine with important functions in immune regulation and hematopoiesis, and plays a significant part in the etiology of several autoimmune disorders (Kishimoto, 2010; Nishimoto and Kishimoto, 2006; Tanaka et al., 2014). Moreover, increased circulating levels of inflammatory factors, such as IL-6, tumor necrosis factor-alpha (TNF-α), and the acute phase protein C-reactive protein (CRP) have been reported in MDD (Goldsmith et al., 2016; Haapakoski et al., 2015; Howren et al., 2009; Miller and Raison, 2016). Following treatment of acute episodes in MDD, IL-6 blood levels have been shown to significantly decrease (Goldsmith et al., 2016). Studies also show that a subgroup of MDD patients characterized by high blood levels of CRP and IL-6, have a suboptimal response to antidepressants with predominantly serotonergic action, but a better response when combining antidepressants with anti-inflammatory agents such as infliximab (Arteaga-Henríquez et al., 2019).

Genetic factors influence MDD, with an estimated heritability from familial studies of around 30–40 % (Sullivan et al., 2000). The heritability attributable to single nucleotide polymorphisms (SNPs) is estimated to be around 10 % (Howard et al., 2018; Romero et al., 2022; Wray et al., 2018). To date, genome-wide association studies (GWAS) have identified hundreds of genetic loci associated with MDD (Als et al., 2023; Howard et al., 2019; Levey et al., 2021). Blood levels of WBCs and IL-6 are also hereditary traits (Chen et al., 2020; Hinckley et al., 2013; Wörns et al., 2006). Recent GWAS have identified more than a thousand loci associated with WBCs (Astle et al., 2016; Chen et al., 2020), with the estimated SNP-heritability ranging from 6.5 % to 20 % for the different WBC types (Chen et al., 2020). A recent GWAS identified three loci associated with IL-6 levels, and estimated a SNP heritability of 4.5 % (Ahluwalia et al., 2021). Intriguingly, polygenic risk scores (PRS) for depression have been linked to increased WBC count, suggesting a shared genetic basis (Sealock et al., 2021). Further, a recent large MDD GWAS reported a significant genetic correlation between WBC count and MDD of 0.14 (Levey et al., 2021). However, the genetic relationship between MDD and these immunological traits remains poorly understood.

Standard statistical methods examining genetic overlap usually focus on estimates of genetic correlation or PRS associations. However, they fail to capture mixed effect directions across shared genetic variants, and therefore give an incomplete picture of overlapping genetic architecture between phenotypes. More recent statistical methods such as bivariate causal mixture model (MiXeR) and conjunctional false discovery rate (conjFDR) enable new opportunities for studying genetic pleiotropy, by detecting and quantifying genetic overlap beyond genetic correlation (Frei et al., 2019; Smeland et al., 2020b). Both these approaches have identified overlapping genetic influences between several complex human traits and disorders in recent years (Andreassen et al., 2023; Hindley et al., 2021; O’Connell et al., 2019; Smeland et al., 2020a; Wiström et al., 2022).

In this exploratory study, we aimed to comprehensively characterize to what extent MDD and the immunological traits IL-6 and WBCs share genetic signal, at both the global and local level, to provide new insights into their relationship. To this end, we applied a set of complementary statistical tools that investigate different aspects of genetic overlap, including linkage disequilibrium (LD) score regression, MiXeR, conjFDR and Mendelian randomization (MR) on recent large-scale GWAS to identify genetic correlations and overlapping genetic underpinnings independent of effect directions (Bulik-Sullivan et al., 2015; Frei et al., 2019; Smeland et al., 2020b). We also conducted PRS analysis leveraging the large-scale Norwegian Mother, Father and Child Cohort Study (MoBa) to further assess the genetic relationship between these immune traits and MDD (Murray et al., 2021; Smeland and Andreassen, 2021).

## Material and methods

### Participant samples and phenotypes

We used GWAS summary statistics (*p* values and *Z*-scores/odds ratios). Data on MDD was provided by a meta-analysis including data from Million Veteran Program (MVP), UK Biobank, 23andMe, and Psychiatric Genomics Consortium (PGC); totaling 330,173 cases and 727,595 controls (Levey et al., 2021). The definition of the MDD phenotype varied across the individual studies; some studies utilized self-reporting, while others relied on electronic health records. See the original GWAS for detailed descriptions (Howard et al., 2019; Levey et al., 2021).

For the PRS analysis, we utilized MoBa as test sample (Magnus et al., 2016). MoBa is a population-based pregnancy cohort study conducted by the Norwegian Institute of Public Health. Participants were recruited from all over Norway from 1999–2008. The women consented to participation in 41 % of the pregnancies. The cohort includes approximately 114.500 children, 95.200 mothers and 75.200 fathers. The current study is based on version 12 of the quality-assured data files released for research in January 2019. We only included parents in our study. Blood samples were obtained from both parents during pregnancy. We identified cases of Major Depressive Disorder (MDD) using data from the Norwegian Patient Registry spanning from 2008 to 2022. Cases were determined based on the presence of a F32 or F33 diagnosis according to the International Classification of Diseases (ICD) 10th revision. We excluded participants with a diagnosis of bipolar disorder or psychosis (any F2 disorder), as well as anyone with a F00-F09 diagnosis. For each related pair of individuals with a kinship coefficient greater than 0.05, one member was excluded (we prioritized cases, otherwise participants were excluded randomly). After accounting for missing data, we were left with 9582 cases with MDD and 84,670 controls (Corfield et al., 2022; Paltiel et al., 2014). In separate PRS analyses, where we added smoking and BMI as covariates, we ended up with 7092 cases and 58,975 controls due to missing data. Smoking was defined as if the participants stated they had ever smoked, and BMI was based on self-reported height and weight.

To assess the robustness of the results, we conducted a sign-concordance test using an independent GWAS of individuals of East Asian ancestry that included 12,588 MDD cases and 85,914 controls (Giannakopoulou et al., 2021). Cases were defined with a variety of methods, including self-reporting, structured clinical interviews and electronic health records. For a more detailed description, see the original GWAS (Giannakopoulou et al., 2021).

GWAS data on basophil, eosinophil, lymphocyte, monocyte, neutrophil and total WBC counts originated from the Blood Cell Consortium (BCX) and comprised *n*_max_ = 563,946 individuals (Chen et al., 2020). The IL-6 sample was provided by the Cohorts for Heart and Aging Research in Genomic Epidemiology (CHARGE) consortium, and comprised 52,654 individuals (Ahluwalia et al., 2021). For exclusion criteria, implementation and quality control of the WBC and IL-6 phenotypes, we refer to our Supplementary Methods and the original publications (Ahluwalia et al., 2021; Chen et al., 2020).

For all analyses, except the validation dataset, we excluded participants of non-European ancestry to ensure linkage disequilibrium (LD) compatibility across the GWAS. For the conjFDR analysis, we excluded cohorts from the MDD sample that were overlapping with the WBC and IL-6 samples.

### Statistical analysis

#### LD score regression

We utilized bivariate LD score regression to estimate the genetic correlation between MDD and the immune phenotypes (Bulik-Sullivan et al., 2015). To adjust for multiple testing, we applied the FDR method of Benjamini-Yekutieli (Benjamini and Yekutieli, 2001) due to the genetic correlation between WBC subgroups (Chen et al., 2020).

#### MiXeR

We applied MiXeR version 1.3 to quantify the polygenicity (the estimated number of causal variants) and the discoverability (causal SNP effect size variance) for each trait, as well as the number of unique and shared variants between two traits (Frei et al., 2019; Holland et al., 2020). MiXeR utilizes GWAS summary statistics and is based on Gaussian mixture models. The model first builds a univariate mixture model for each trait, where it estimates the amount of causal SNP variants that explain 90 % of the SNP heritability to avoid misrepresentative results of variants with insignificant effect sizes. The bivariate model then estimates the number of shared variants between the two traits irrespective of effect directions. The results are presented as Venn diagrams. Point estimates and standard deviations are computed by conducting 20 iterations with 2 million random SNPs followed by random pruning at an r^2^ threshold of 0.8.

The model fit for the univariate MiXeR is evaluated by calculating the difference between the Akaike information criterion (AIC) for the MiXeR estimates and a model in which all variants are assumed to be causal as a reference. Positive AIC differences are interpreted as evidence that the best-fitting MiXeR estimates are discernible from the reference model. The same calculation is used to evaluate the model fit for the bivariate MiXeR, only that the best fitting model is compared to both minimum possible overlap and maximum possible overlap as a reference instead.

#### ConjFDR

To improve power for identifying shared genomic loci, we applied the conjFDR method. The conjFDR method is an extension of the conditional FDR (condFDR) method, which increases pleiotropic discovery by leveraging cross trait enrichment between two phenotypes (Smeland et al., 2020b). The condFDR re-ranks the test statistics in a primary phenotype conditioned on the associations with a secondary phenotype. Reversing the process, shifting the primary and secondary phenotypes, and then selecting the maximum of the two condFDR values provides a conservative estimate of the FDR for association with both phenotypes, defined as the conjFDR. We set the threshold for significant loci at conjFDR *<*0.05 in accordance with previous literature (Bahrami et al., 2020; Hindley et al., 2023; Smeland et al., 2020a; Smeland et al., 2017; Smeland et al., 2020b; Steen et al., 2023).

Quantile-quantile (Q-Q) plots are used to determine if there is a cross-trait enrichment between the two phenotypes analyzed (Smeland et al., 2020b). The graph shows the distribution of -log *p* values for the primary phenotype for all SNPs, and for subset of SNPs conditioned of their association with the secondary phenotype. Enrichment is evident by visual inspection if there is an increased proportion of lower *p* values of SNPs associated with the primary phenotype as a function of association with the secondary phenotype.

We controlled for spurious enrichment by random pruning, which was averaged over 500 iterations, where one SNP in each LD block (r^2^ *>* 0.1) was randomly selected for each iteration. We excluded SNPs within the major histocompatibility complex (MHC; genome build 19 location 25,119,106–33,854,733) and the chromosomal region 8p23.1 (location 7200,000–12,500,000) due to their complex LD structures, to avoid biased FDR estimation (Schwartzman and Lin, 2011).

#### PRS

Additionally, we utilized PRS to test the predictive ability of MDD case control status based on risk variants for WBC phenotypes, IL-6 and MDD. PRS quantifies an individual’s summary genetic risk estimate based on risk alleles that an individual carries, weighted by effect sizes determined by a GWAS for a given trait (Choi et al., 2020). Examining the PRS in large pools of individuals can be used for further analyses of differences in PRS scores for different subgroups. We utilized PRSice (2.3.3) to calculate PRS *p* threshold = 5e-8, 1e-6, 1e-5, 1e-4, 1e-3, 1e-2, 5e-2, 1e-1, 5e-1, 1) from GWAS of MDD (Choi and O’Reilly, 2019; Howard et al., 2018), WBCs and IL-6. Then, based on the principal component approach described by Coombes et al (Coombes et al., 2020), we extracted the first principal component for each PRS across all *p* threshold-values. We also did separate analyses where we added smoking and BMI as covariates.

#### MR

We conducted MR to explore the potential causal relationship between MDD and the immune phenotypes. We performed bidirectional MR using GWAS summary statistics for MDD and each immune trait as exposure and outcome of interest. We used the R package TwoSam-pleMR (Hemani et al., 2018), and report the results using three different methods (i) inverse variance weighted, (ii) weighted median and (iii) MR Egger (Bowden et al., 2015; Bowden et al., 2016; Burgess et al., 2013). We applied the FDR correction for multiple comparisons using the Benjamini-Hochberg method (Benjamini and Hochberg, 1995).

#### Validation in east asian dataset

We performed a sign concordance test of all lead SNPs detected in our conjFDR analyses using an independent East Asian MDD sample (Giannakopoulou et al., 2021), and evaluated whether their effect directions were consistent in the two datasets. We assessed statistical significance utilizing the exact binomial one-sided test.

### Locus definition

We defined a genomic locus according to the FUMA protocol for functional annotation of SNPs and genes (Watanabe et al., 2017). We identified independent significant SNPs as variants with conjFDR *<*0.05 and LD r^2^ *<*0.6 with each other. A subset of these variants at approximately LD r^2^ *<* 0.1 of each other, were considered lead SNPs. Candidate SNPs were defined as all SNPs in LD r^2^ ≥ 0.6 with the lead variant, and determined the borders of each locus. Loci separated by less than 250 kb were merged. The most significant SNP was considered the lead SNP of the merged locus. Overlapping signals within complex LD regions were represented by one independent lead SNP only. All LD information was calculated from the 1000 Genomes Project European-ancestry reference haplotype reference panel. We evaluated directional effects of the loci by comparing their *Z-*scores and odds ratios.

### Functional annotation

We used the platform Open Targets Genetics (https://genetics.opentargets.org/) to link genomic loci to the most likely causal genes (Ghoussaini et al., 2021). Open Targets is an open-access web platform which utilizes a machine learning model to assign genes to genomic loci based on GWAS data and functional genomics data, including gene expression, chromatin interaction, protein abundance and conformation data from a wide array of cell types and tissues. The likelihood of a gene’s association with a given locus is quantified by the variant to gene (V2G) score. We included the gene with the highest V2G score in our study. If there were two genes with the same score, both genes were included. We also utilized Open Targets to assess the tractability of the implicated genes, which refers to the suitability of a potential target for various therapeutic interventions (Brown et al., 2018).

Applying FUMA and Genotype-Tissue Expression (GTEx) dataset v8, we conducted gene expression and gene-set analysis of the identified genes (GTEx Consortium 2017; Lonsdale et al., 2013; Watanabe et al., 2017). We also applied FUMA to functionally annotate each candidate SNP with a conjFDR value *<*0.10 based on combined annotation dependent depletion score, which predicts how deleterious the SNP effect is on protein structure/function, RegulomeDB score, which estimates the probability of the SNP having a regulatory function, and chromatin states, which predicts transcription/regulatory effects from chromatin states at the SNP locus. Using a publicly available RNA-sequencing and splicing database, we also determined the expression of the top genes identified by Open Targets in six different cell types in the human cerebral cortex, including fetal astrocytes, mature astrocytes, neurons, oligodendrocytes, endothelial cells and microglia (Zhang et al., 2016).

## Results

### Genetic correlation

We found a significant positive correlation between MDD and IL-6 using LD score regression (*r*_g_ = 0.22, corrected *p* = 0.001) at FDR *<* 0.05 (Supplementary Table 1). There was a negative correlation between MDD and eosinophils (*r*_g_ = −0.04, corrected *p* = 0.02). While neutrophils and total WBC both had a positive correlation with MDD, this did not survive correction for multiple testing (Supplementary Table 1). No other WBC subgroup had a significant genetic correlation with MDD.

### Shared and distinct genetic architecture

**Fig. 1.**
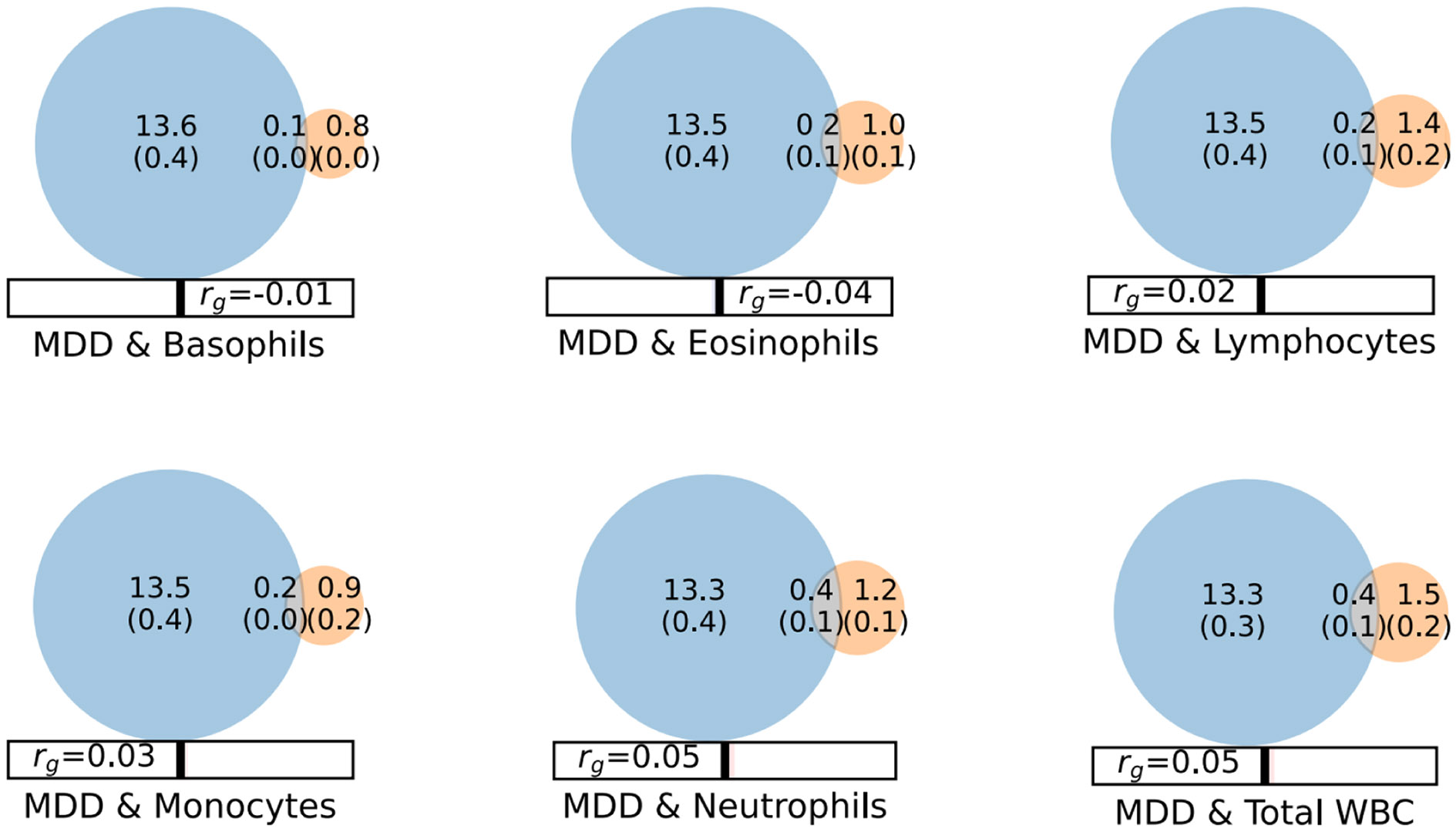
MiXeR Venn diagrams illustrate the estimates (in thousands) of causal SNPs to account for the heritability of major depressive disorder (MDD) (blue circles) and the different white blood cell (WBC) subgroups (orange circles). Estimated overlapping SNPs are represented with the grey area, r_g_ is the genome-wide genetic correlation.

The univariate MiXeR model estimated greatest polygenicity for MDD with *n* = 13.7K (SD 0.4K) variants. For the WBC phenotypes, estimates ranged from *n* = 0.8K (SD 0.03K) variants for basophils as the lowest, to *n* = 1.9K (SD 0.1K) variants for total WBC as the highest (Supplementary Table 2). For IL-6 we estimated *n* = 0.2K (SD 0.5K) variants. The bivariate MiXeR analysis estimated partial overlap between MDD and the WBC phenotypes, with 0.08K (SD 0.03K) out of 0.8K (10.1 %) basophil variants overlapping with MDD, 0.2K (SD 0.08K) out of 1.0K (19.7 %) eosinophil variants, 0.2K (SD 0.1K) out of 1.4K (15.7 %) lymphocyte variants, 0.2K (SD 0.04K) out of 0.9K (19.1 %) monocyte variants, 0.4K (SD 0.1K) out of 1,2K (33.4 %) neutrophil variants and 0.4K (SD 0.1K) out of 1.4K (27.6 %) total WBC variants (Fig. 1). The MiXeR model indicated that only 18 % and 27 % of the shared variants between MDD and eosinophils and basophils had concordant effect directions, respectively (Supplementary Table 3). The concordance rates among the shared variants with MDD for the other WBC subgroups ranged from 68 % for lymphocytes to 79 % for monocytes. These estimates were in accordance with the overall genetic correlations estimated using LD score regression (Supplementary Table 1). The bivariate MiXeR analysis for MDD and IL-6 displayed poor model fit with negative AIC scores, likely due to the low polygenicity of IL-6 compared to MDD (Supplementary Table 4).

### Genetic loci and functional analysis

In line with the MiXeR results, the conditional Q-Q plots demonstrated cross-trait enrichment between MDD and every subgroup of WBCs, as well as between MDD and IL-6, indicating polygenic overlap. This is evident from the increasing leftward deflection of the Q-Q plot curves, with increasing levels of SNP associations for MDD conditioned on increasing SNP associations with the auxiliary phenotypes (Supplementary Figures 1a-g).

Using conjFDR analysis, we identified MDD risk loci shared with basophils (8), eosinophils (17), lymphocytes (23), monocytes (14), neutrophils (20) and total WBC (20), see Supplementary Table 5-10. There were 22 loci that were linked to at least two different WBC subgroups as well as MDD, three loci were linked to five different WBC subgroups. The loci had mixed effect directions, with mainly concordant effect directions in 65 % for lymphocytes (15/23) and total WBC (13/20), and 64 % for monocytes (9/14), an even split between concordant and discordant effect directions in basophils (4/8) and neutrophils (10/20), while eosinophils had a 41 % proportion of concordant effect directions (7/17). We found two loci shared between MDD and IL-6, one of which had concordant effect directions (Supplementary Table 11).

For information on the genes implicated by the lead SNPs in Open Targets, see Supplementary Tables 5-11. Notably, five of the implicated genes encode proteins that are either targets of approved drugs (*ESR2*), or drugs which have been investigated in clinical trials (*KDM1A, XPNPEP3, LY75* and *NOX4*), see Supplementary Table 12. FUMA analysis of the top genes identified by Open Targets showed that genes implicated by the shared loci between MDD and eosinophils were significantly downregulated in the anterior cingulate cortex of the brain, while the genes implicated for MDD and monocytes were significantly downregulated in the salivary glands. The gene set Transcription Factor targets (MsigDB c3) was linked to genes implicated by the loci shared between MDD and total WBC. All top genes identified by Open Targets were expressed in the human cerebral cortex (Zhang et al., 2016). For detailed information of their different cell-type specific expressions, see Supplementary Figure 3. For functional annotation of all candidate SNPs, see Supplementary Tables 13-19. Most of the candidate SNPS were intronic or intergenic. However, 114 candidate SNPs were exonic, that is, located in the protein coding part of the gene. Of these exonic SNPs, 73 were nonsynonymous, implying that they result in a change to the protein’s amino acid sequence. A total of 45 of the exonic candidate SNPs had a CADD score *>*12.37, suggesting deleteriousness, (Kircher et al., 2014).

### Mendelian randomization

The bidirectional MR analysis revealed no statistically significant associations (Supplementary Table 20), demonstrating that no causal association between MDD and the immune phenotypes could be detected using the current GWAS samples.

### Polygenic risk score

The PRS analysis showed a small but significant increased risk for MDD associated with PRS for monocytes (beta = 0.04, corrected *p* = 6×10^−4^), see Fig. 3 and Supplementary Table 21. The result was still significant after we added BMI and smoking as covariates (Supplementary Figure 2 and Supplementary Table 22). While PRS analysis also showed a weak increased risk for MDD associated with basophils, lymphocytes, neutrophils, and total WBC, this did not remain significant after FDR correction. Eosinophils and IL-6 showed no significant predictive value of risk.

### Sign concordance test

There were 67 SNPs present in both the discovery and validation dataset out of the 76 discovered lead SNPs. Of these SNPs, 44 (65.6 %) had concordant allelic effect directions, which is significantly higher than expected by chance (*p* = 0.007), supporting the robustness of the results.

## Discussion

In the present study, we characterized and dissected the shared genetic architecture of MDD and immunological phenotypes, revealing different patterns of shared genetic influences across WBC counts and IL-6 levels. Applying a complementary set of statistical tools, we quantified the shared genetic underpinnings between MDD and these immunological traits, and we identified several overlapping loci between the phenotypes. While our findings suggest that common genetic variants may jointly contribute to immune dysregulation and increased risk of MDD, this is likely to only be applicable in a minority of patients with MDD, since the shared variants only account for a small part of the genetic risk architecture of MDD.

Our univariate MiXeR analysis indicated substantial differences in the polygenicities of the phenotypes, which governs the extent of which complex phenotypes may share genetic variants. While IL-6 was the least polygenic trait with only 0.2K estimated causal variants, the polygenicity for WBCs ranged between 0.8K variants for basophils to 1.5K variants for total WBC, and we estimated MDD to be influenced by 13.7K variants, which is in line with previous studies (Hindley et al., 2023; Steen et al., 2023). Despite the disparate polygenicity estimates, bivariate MiXeR estimated that MDD and different WBC subgroups shared between 0.08K to 0.4K common genetic variants.

Our results provide further evidence that MDD and these immunological traits are genetically linked (Levey et al., 2021; Sealock et al., 2021), highlighted by the genetic correlation of 0.22 found between MDD and IL-6. Our study revealed a spectrum of genetic relationship between MDD and the different WBC subgroups. While around 70–80 % of the overlapping genetic variants with neutrophils, monocytes, lymphocytes, and total WBC had concordant effect directions, about 70–80 % of the overlapping variants with basophils and eosinophils had discordant effect directions. The LD score regression estimates of global genetic correlations were in line with these MiXeR estimates, although only the negative correlation between MDD and eosinophils reached significance (*r*_g_ = −0.04, *p* = 0.004). The results are consistent with clinical studies reporting that circulating neutrophils and monocytes are elevated in depressed patients, while there has been little evidence of increased basophil or eosinophil counts (Foley et al., 2023; Maes et al., 1992; Seidel et al., 1996). Elevated neutrophils and monocytes, together with elevated levels of proinflammatory cytokines and acute phase proteins, may reflect the activation of the innate immune system (Beurel et al., 2020; Grosse et al., 2015; Miller et al., 2009). This is supported by the fact that 18 of the loci that we found to be overlapping between MDD and total WBC were also identified in a recent conjFDR study investigating the genetic overlap between the acute phase protein CRP and psychiatric disorders (Hindley et al., 2023). MDD has also been shown to be associated with abnormalities in lymphocytes including increased number of CD4^+^ T cells and a reduction of regulatory B cells and regulatory T cells, indicating an imbalance in the adaptive immune system (Ahmetspahic et al., 2018; Foley et al., 2023; Li et al., 2010; Lynall et al., 2020), which is in line with the concordant effect directions found among lymphocytes in our bivariate MiXeR analysis.

**Fig. 2.**
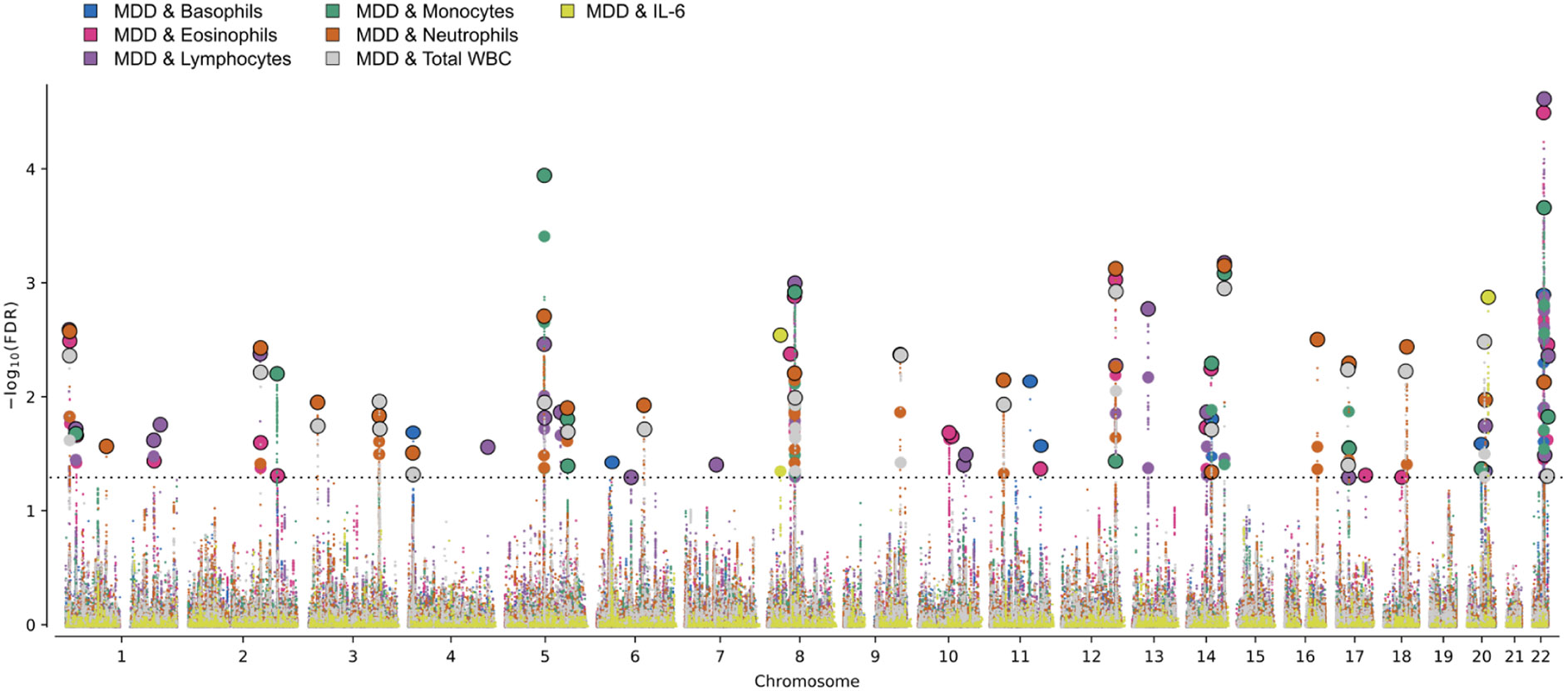
Conjunctional FDR (conjFDR) Manhattan plot illustrating the common genetic variants shared between major depressive disorder (MDD) and basophils (blue dots), eosinophils (pink dots), lymphocytes (purple dots), monocytes (green dots), neutrophils (orange dots), total white blood cells (WBC) (grey dots) and interleukin 6 (IL-6) (yellow dots). The *x*-axis represents chromosomal position, while the *y*-axis displays -log_10_ transformed FDR *p* values. The black dotted horizontal line represents cutoff for significant FDR at conjFDR*<*0.05. Independent lead SNPs are circled in black.

Among the multiple MDD risk loci found to overlap with WBCs using conjFDR (Fig. 2), three loci were shared with five WBC subgroups. The first locus on chromosome 8q12.2 (top lead SNP rs4737565) implicates the chromodomain helicase DNA binding protein 7 (*CHD7*) gene. This gene has important roles in chromatin remodeling in neurons, for which mutations can lead to the developmental disorder CHARGE syndrome, which includes a variety of neurodevelopmental deficiencies, and has been linked to immunodeficiency and reduced number of T cells (Goodman and Bonni, 2019; Liu et al., 2018; Reddy et al., 2021). The second locus on chromosome 12q24.31 (top lead SNP rs77741769) implicates the signal peptide peptidase-like 3 (*SPPL3*) gene, which is also related to T cell activity (Jongsma et al., 2021), and the 2′-5′-oligoadenylate synthetase like (*OASL*) gene, which is linked to regulation of interferon production (Ghosh et al., 2019). The third of these loci on chromosome 14q24.3 (top lead SNP rs2003490) implicates the dihydrolipoamide S-succinyltransferase (*DLST*) gene and the ribosomal protein S6 kinase like 1 (*RPS6KL1*) gene. *DLST* encodes an enzyme involved in the tricarboxylic acid cycle (Shen et al., 2021), and is also involved in the metabolic fine-tuning and survival of B lymphocytes (Diaz-Muñoz et al., 2015). In general, *RPS6KL1* controls cell survival and apoptosis (MacKeigan et al., 2005), but has also been implicated in the cytokine network regulation of chronic inflammatory states, such as coronary artery disease (Bon-Baret et al., 2021).

Using conjFDR analysis, we identified two overlapping loci between MDD and IL-6, both of which are novel for IL-6 to our knowledge. Previously, only two genomic loci outside the IL-6 gene have been associated with IL-6 (Ahluwalia et al., 2021), while the IL-6 gene itself was not implicated by our present analysis. The shared locus located on chromosomal region 20q13.13 (lead SNP rs2295714) had discordant effect directions with MDD and IL-6, and implicated the gene staufen1 (*STAU1)*, which specifically regulates several genes involved in inflammatory and immune response regulation (Diaz-Muñoz et al., 2015). It also plays important roles in controlling the post-transcriptional modification of autophagy-related RNA species, and is linked to several neurodegenerative disorders (Heraud-Farlow and Kiebler, 2014; Paul et al., 2023). The other shared locus on chromosome 8p21.3 (lead SNP rs7824087) had concordant effect directions with MDD, but had insufficient evidence to implicate any specific gene (Ghoussaini et al., 2021). Notably, the loci associated with IL-6 did not overlap with any of the WBC loci. Identifying genes associated with both MDD and markers of immune dysfunction could reveal potential targets for future drug treatment. Recent research on MDD have increasingly focused on drugs that modulate the immune system, with encouraging results for nonsteroidal anti-inflammatory agents and antibodies targeting cytokines among others (Drevets et al., 2022; Simon et al., 2023). One potential druggable target identified by our study is the estrogen receptor 2 (*ESR2*) gene, implicated by a locus shared between MDD and lymphocytes. The *ESR2* gene is highly expressed in lymphocytes, and have important roles in the regulation of both the innate and adaptive immune system, as well as in immune cell development (Kovats, 2015; Scariano et al., 2008). Interestingly, estrogens have recently been suggested as potential therapeutic options to treat depression owing to genetic support (Parker et al., 2024b).

Functional annotation identified candidate SNPs more likely to substantially impact the phenotypes investigated, making them promising targets for follow-up studies (Supplementary Tables 13-19). Notable examples of promising candidate SNPs are rs2286913 and rs7156590, both of which are nonsynonymous, located in the exon part of the above mentioned *RPS6KL1* gene, and have CADD scores above 12.37, indicating deleteriousness (Kircher et al., 2014). All top genes implicated by the identified loci are significantly expressed in the human cerebral cortex (Zhang et al., 2016), but with different expression patterns across specific cell types (Supplementary Figure 3), underscoring the relevance of the cerebral cortex for the shared genetic influences between MDD and these immunological traits (Parker et al., 2024a). While our MiXeR, LD score regression and conjFDR findings indicate that MDD, WBC count and IL-6 levels partly share genetic signal, the MR analysis was unable to detect any significant causal association between the phenotypes. This indicates that the increased WBC count and IL-6 levels observed in some MDD patients may not be causally linked to the disorder but reflect common underlying biological processes. However, we cannot exclude a causal relationship between these immunological phenotypes, warranting follow-up studies using larger GWAS datasets or subgroups of patients.

The shared genetic variants only account for a minor proportion of the genetic architecture of MDD, and it remains unclear whether immunological processes related to WBCs and IL-6 represent relevant pathophysiological mechanisms underlying MDD. The same may apply to other immunological traits like blood levels of CRP and immune-mediated diseases such as inflammatory bowel disease and type 2 diabetes, which also are considerably less polygenic than MDD (Hindley et al., 2023; Smeland et al., 2023). MDD might consist of several distinct subgroups with different genetic architectures, and that immune dysregulation may be relevant to a subgroup or smaller portion of patients with depression, for instance patients with atypical or melancholic depression (Dunjic-Kostic et al., 2013; Karlović et al., 2012; Miller and Raison, 2016), or more severe forms of depression (Lamers et al., 2019; Vogelzangs et al., 2014). By comparison, the number of overlapping variants between MDD and WBCs was less than half of that observed between WBCs and schizophrenia, another severe psychiatric disorder, which ranged between 0.3K variants for basophils to 1.0K for lymphocytes (Steen et al., 2023).

**Fig. 3.**
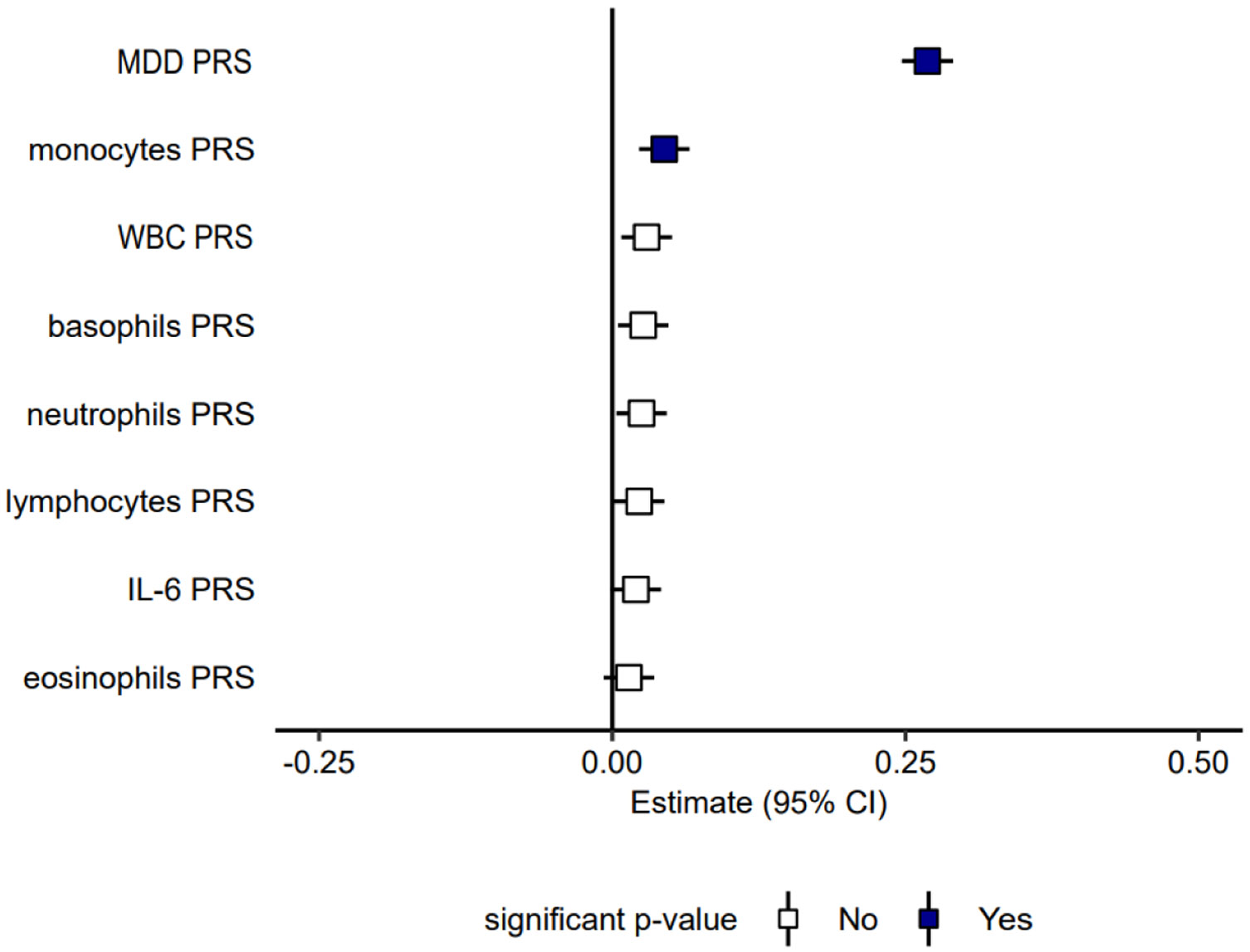
Polygenic risk scores (PRS) for major depressive disorder (MDD) and immunological phenotypes (WBC counts and IL-6) and their association with MDD casecontrol status in the MoBa sample (9582 cases, 84,670 controls). The *x*-axis shows effect sizes for standardized PRS. The black squares denote significant findings after FDR correction for multiple comparisons.

We also assessed whether PRS based on the immunological traits could predict cases with MDD in the MoBa sample (Fig. 3). While the PRS for monocytes was significantly associated with MDD, the effect size was small, suggesting little clinical relevance. The low polygenicity of the immunological traits, which captures only a minor fraction of the MDD risk architecture, as well as global genetic correlations close to zero, likely explains the inability of the immunological PRS to predict MDD status.

There are some limitations to our study. While we estimated a significant positive genetic correlation of 0.22 between MDD and IL-6, the very low polygenicity of IL-6 estimated using MiXeR indicates that this correlation estimate should be interpreted cautiously, given that the LD score regression method assumes an infinitesimal model with all SNPs having an effect (Bulik-Sullivan et al., 2015). MDD, WBCs and IL-6 have been associated with smoking and BMI (Beurel et al., 2020), which may confound some of the associations reported here. All participants in our study were of European ancestry, and the results might not translate to other ancestry groups, as the genetic architecture of MDD is shown to imperfectly correlate across ancestries (Giannakopoulou et al., 2021). However, comparing the effect directions of the lead MDD SNPs in an independent MDD GWAS dataset with individuals of East Asian ancestry showed that the majority of effect directions were concordant (Giannakopoulou et al., 2021), implying that our results may be generalizable to other ancestries. While we applied state-of-the-art tools for mapping genomic loci to causal genes such as Open Targets, there is still challenges in translating associated loci to the causal genes (Ghoussaini et al., 2021). Similarly, the conjFDR identifies a genomic region jointly associated with two traits, which could harbor both shared or separate causal variants (Smeland et al., 2020b).

In conclusion, we showed that MDD shares genetic architecture with the immunological traits WBC counts and IL-6 blood levels, related to a small part of the genetic susceptibility of MDD. The findings suggest a complex genetic relationship between these phenotypes with a mixed pattern of effect directions, and a diverse pattern of overlap across the different types of WBCs. This provided a better understanding of the pathophysiology of MDD related to the immune system and should be followed up with experimental studies.

## Supplementary Material

1

2

3

Supplementary material associated with this article can be found, in the online version, at doi:10.1016/j.jadr.2025.100889.

## Abbreviations:

MDD: major depressive disorder
WBC: White blood cell
MiXeR: bivariate causal mixture model
conjFDR: conjunctional false discovery rate
condFDR: conditional FDR
LD: linkage disequilibrium
PRS: polygenic risk score
MoBa: Norwegian Mother, Father and Child Cohort Study

## References

[R1] Ahluwalia TS, Prins BP, Abdollahi M, Armstrong NJ, Aslibekyan S, Bain L, Jefferis B, Baumert J, Beekman M, Ben-Shlomo Y, 2021. Genome-wide association study of circulating interleukin 6 levels identifies novel loci. Hum. Mol. Genet 30 (5), 393–409. 33517400 10.1093/hmg/ddab023 PMC8098112

[R2] Ahmetspahic D, Schwarte K, Ambrée O, Bürger C, Falcone V, Seiler K, Kooybaran MR, Grosse L, Roos F, Scheffer J, 2018. Altered B cell homeostasis in patients with major depressive disorder and normalization of CD5 surface expression on regulatory B cells in treatment responders. J. Neuroimmune Pharmacol 13, 90–99. 28905187 10.1007/s11481-017-9763-4

[R3] Als TD, Kurki MI, Grove J, Voloudakis G, Therrien K, Tasanko E, Nielsen TT, Naamanka J, Veerapen K, Levey DF, 2023. Depression pathophysiology, risk prediction of recurrence and comorbid psychiatric disorders using genome-wide analyses. Nat. Med 29 (7), 1832–1844. 37464041 10.1038/s41591-023-02352-1 PMC10839245

[R4] Andreassen OA, Hindley GF, Frei O, Smeland OB, 2023. New insights from the last decade of research in psychiatric genetics: discoveries, challenges and clinical implications. World Psychiatry 22 (1), 4–24. 36640404 10.1002/wps.21034 PMC9840515

[R5] Arteaga-Henríquez G, Simon MS, Burger B, Weidinger E, Wijkhuijs A, Arolt V, Birkenhager TK, Musil R, Müller N, Drexhage HA, 2019. Low-grade inflammation as a predictor of antidepressant and anti-inflammatory therapy response in MDD patients: a systematic review of the literature in combination with an analysis of experimental data collected in the EU-MOODINFLAME consortium. Front. Psychiatry 10, 458. 31354538 10.3389/fpsyt.2019.00458 PMC6630191

[R6] Astle WJ, Elding H, Jiang T, Allen D, Ruklisa D, Mann AL, Mead D, Bouman H, Riveros-Mckay F, Kostadima MA, 2016. The allelic landscape of human blood cell trait variation and links to common complex disease. Cell 167 (5), 1415–1429 e1419. 27863252 10.1016/j.cell.2016.10.042 PMC5300907

[R7] Bahrami S, Steen NE, Shadrin A, O’Connell K, Frei O, Bettella F, Wirgenes KV, Krull F, Fan CC, Dale AM, 2020. Shared genetic loci between body mass index and major psychiatric disorders: a genome-wide association study. JAMA Psychiatry 77 (5), 503–512. 31913414 10.1001/jamapsychiatry.2019.4188 PMC6990967

[R8] Benjamini Y, Hochberg Y, 1995. Controlling the false discovery rate: a practical and powerful approach to multiple testing. J. R. Stat. Soc.: Ser. B (Methodol.) 57 (1), 289–300.

[R9] Benjamini Y, Yekutieli D, 2001. The control of the false discovery rate in multiple testing under dependency. Ann. Stat 1165–1188.

[R10] Beurel E, Toups M, Nemeroff CB, 2020. The bidirectional relationship of depression and inflammation: double trouble. Neuron 107 (2), 234–256. 32553197 10.1016/j.neuron.2020.06.002 PMC7381373

[R11] Bon-Baret V, Chignon A, Boulanger M-C, Li Z, Argaud D, Arsenault BJ, Thériault S, Bossé Y, Mathieu P, 2021. System genetics including causal inference identify immune targets for coronary artery disease and the lifespan. Circ.: Genom. Precis. Med 14 (2), e003196. 33625251 10.1161/CIRCGEN.120.003196 PMC8284374

[R12] Bowden J, Davey Smith G, Burgess S, 2015. Mendelian randomization with invalid instruments: effect estimation and bias detection through Egger regression. Int. J. Epidemiol 44 (2), 512–525. 26050253 10.1093/ije/dyv080 PMC4469799

[R13] Bowden J, Davey Smith G, Haycock PC, Burgess S, 2016. Consistent estimation in mendelian randomization with some invalid instruments using a weighted median estimator. Genet. Epidemiol 40 (4), 304–314. 27061298 10.1002/gepi.21965 PMC4849733

[R14] Brown KK, Hann MM, Lakdawala AS, Santos R, Thomas PJ, Todd K, 2018. Approaches to target tractability assessment–a practical perspective. Medchemcomm 9 (4), 606–613. 30108951 10.1039/c7md00633k PMC6072525

[R15] 3G.C.f.A.N.o.t.W.T.C.C.C. Bulik-Sullivan B, Finucane HK, Anttila V, Gusev A, Day FR, Loh P-R, Consortium, R., Consortium, P.G., Duncan L, 2015. An atlas of genetic correlations across human diseases and traits Nat. Genet 47 (11), 1236–1241. 26414676 10.1038/ng.3406 PMC4797329

[R16] Burgess S, Butterworth A, Thompson SG, 2013. Mendelian randomization analysis with multiple genetic variants using summarized data. Genet. Epidemiol 37 (7), 658–665. 24114802 10.1002/gepi.21758 PMC4377079

[R17] Chen M-H, Raffield LM, Mousas A, Sakaue S, Huffman JE, Moscati A, Trivedi B, Jiang T, Akbari P, Vuckovic D, 2020. Trans-ethnic and ancestry-specific blood-cell genetics in 746,667 individuals from 5 global populations. Cell 182 (5), 1198–1213 e1114. 32888493 10.1016/j.cell.2020.06.045 PMC7480402

[R18] Choi SW, Mak TS-H, O’Reilly PF, 2020. Tutorial: a guide to performing polygenic risk score analyses. Nat. Protoc 15 (9), 2759–2772. 32709988 10.1038/s41596-020-0353-1 PMC7612115

[R19] Choi SW, O’Reilly PF, 2019. PRSice-2: polygenic Risk Score software for biobank-scale data. Gigascience 8 (7), giz082. 31307061 10.1093/gigascience/giz082 PMC6629542

[R20] Coombes BJ, Ploner A, Bergen SE, Biernacka JM, 2020. A principal component approach to improve association testing with polygenic risk scores. Genet. Epidemiol 44 (7), 676–686. 32691445 10.1002/gepi.22339 PMC7722089

[R21] Corfield EC, Frei O, Shadrin AA, Rahman Z, Lin A, Athanasiu L, Akdeniz BC, Hannigan L, Wootton RE, Austerberry C, 2022. The Norwegian mother, Father, and Child cohort study (MoBa) genotyping data resource: MoBaPsychGen pipeline v. bioRxiv 1, 2022.2006. 2023.496289.

[R22] Cui L, Li S, Wang S, Wu X, Liu Y, Yu W, Wang Y, Tang Y, Xia M, Li B, 2024. Major depressive disorder: hypothesis, mechanism, prevention and treatment. Signal. Transduct. Target. Ther 9 (1), 1–32. 38331979 10.1038/s41392-024-01738-y PMC10853571

[R23] Diaz-Muñoz MD, Bell SE, Fairfax K, Monzon-Casanova E, Cunningham AF, Gonzalez-Porta M, Andrews SR, Bunik VI, Zarnack K, Curk T, 2015. The RNA-binding protein HuR is essential for the B cell antibody response. Nat. Immunol 16 (4), 415–425. 25706746 10.1038/ni.3115 PMC4479220

[R24] Drevets WC, Wittenberg GM, Bullmore ET, Manji HK, 2022. Immune targets for therapeutic development in depression: towards precision medicine. Nat. Rev. Drug Discov 21 (3), 224–244. 35039676 10.1038/s41573-021-00368-1 PMC8763135

[R25] Dunjic-Kostic B, Ivkovic M, Radonjic NV, Petronijevic ND, Pantovic M, Damjanovic A, Poznanovic ST, Jovanovic A, Nikolic T, Jasovic-Gasic M, 2013. Melancholic and atypical major depression—Connection between cytokines, psychopathology and treatment. Prog. Neuro-Psychopharmacol. Biol. Psychiatry 43, 1–6. 10.1016/j.pnpbp.2012.11.009 23200828

[R26] Flannagan RS, Jaumouillé V, Grinstein S, 2012. The cell biology of phagocytosis. Annu. Rev. Pathol.: Mech. Dis 7, 61–98. 10.1146/annurev-pathol-011811-132445 21910624

[R27] Foley ÉM, Parkinson JT, Mitchell RE, Turner L, Khandaker GM, 2023. Peripheral blood cellular immunophenotype in depression: a systematic review and meta-analysis. Mol. Psychiatry 28 (3), 1004–1019. 36577838 10.1038/s41380-022-01919-7 PMC10005954

[R28] Frei O, Holland D, Smeland OB, Shadrin AA, Fan CC, Maeland S, O’Connell KS, Wang Y, Djurovic S, Thompson WK, 2019. Bivariate causal mixture model quantifies polygenic overlap between complex traits beyond genetic correlation. Nat. Commun 10 (1), 2417. 31160569 10.1038/s41467-019-10310-0 PMC6547727

[R29] Ghosh A, Shao L, Sampath P, Zhao B, Patel NV, Zhu J, Behl B, Parise RA, Beumer JH, O’Sullivan RJ, 2019. Oligoadenylate-synthetase-family protein OASL inhibits activity of the DNA sensor cGAS during DNA virus infection to limit interferon production. Immunity 50 (1), 51–63 e55. 30635239 10.1016/j.immuni.2018.12.013 PMC6342484

[R30] Ghoussaini M, Mountjoy E, Carmona M, Peat G, Schmidt EM, Hercules A, Fumis L, Miranda A, Carvalho-Silva D, Buniello A, 2021. Open Targets Genetics: systematic identification of trait-associated genes using large-scale genetics and functional genomics. Nucleic. Acids. Res 49 (D1), D1311–D1320. 33045747 10.1093/nar/gkaa840 PMC7778936

[R31] Giannakopoulou O, Lin K, Meng X, Su M-H, Kuo P-H, Peterson RE, Awasthi S, Moscati A, Coleman JR, Bass N, 2021. The genetic architecture of depression in individuals of East Asian ancestry: a genome-wide association study. JAMA Psychiatry 78 (11), 1258–1269. 34586374 10.1001/jamapsychiatry.2021.2099 PMC8482304

[R32] Gibney SM, Drexhage HA, 2013. Evidence for a dysregulated immune system in the etiology of psychiatric disorders. J. Neuroimmune Pharmacol 8 (4), 900–920. 23645137 10.1007/s11481-013-9462-8

[R33] Goldsmith D, Rapaport M, Miller B, 2016. A meta-analysis of blood cytokine network alterations in psychiatric patients: comparisons between schizophrenia, bipolar disorder and depression. Mol. Psychiatry 21 (12), 1696–1709. 26903267 10.1038/mp.2016.3 PMC6056174

[R34] Goodman JV, Bonni A, 2019. Regulation of neuronal connectivity in the mammalian brain by chromatin remodeling. Curr. Opin. Neurobiol 59, 59–68. 31146125 10.1016/j.conb.2019.04.010 PMC6879819

[R35] Goodnow CC, Vinuesa CG, Randall KL, Mackay F, Brink R, 2010. Control systems and decision making for antibody production. Nat. Immunol 11 (8), 681–688. 20644574 10.1038/ni.1900

[R36] Grosse L, Carvalho LA, Wijkhuijs AJ, Bellingrath S, Ruland T, Ambrée O, Alferink J, Ehring T, Drexhage HA, Arolt V, 2015. Clinical characteristics of inflammation-associated depression: monocyte gene expression is age-related in major depressive disorder. Brain Behav. Immun 44, 48–56. 25150007 10.1016/j.bbi.2014.08.004

[R37] Consortium, GTEx, 2017. Genetic effects on gene expression across human tissues. Nature 550 (7675), 204–213. 29022597 10.1038/nature24277 PMC5776756

[R38] Haapakoski R, Mathieu J, Ebmeier KP, Alenius H, Kivimäki M, 2015. Cumulative meta-analysis of interleukins 6 and 1β, tumour necrosis factor α and C-reactive protein in patients with major depressive disorder. Brain Behav. Immun 49, 206–215. 26065825 10.1016/j.bbi.2015.06.001 PMC4566946

[R39] Hemani G, Zheng J, Elsworth B, Wade KH, Haberland V, Baird D, Laurin C, Burgess S, Bowden J, Langdon R, 2018. The MR-Base platform supports systematic causal inference across the human phenome. Elife 7, e34408. 29846171 10.7554/eLife.34408 PMC5976434

[R40] Heraud-Farlow JE, Kiebler MA, 2014. The multifunctional Staufen proteins: conserved roles from neurogenesis to synaptic plasticity. Trends. Neurosci 37 (9), 470–479. 25012293 10.1016/j.tins.2014.05.009 PMC4156307

[R41] Hinckley JD, Abbott D, Burns TL, Heiman M, Shapiro AD, Wang K, Paola JD, 2013. Quantitative trait locus linkage analysis in a large Amish pedigree identifies novel candidate loci for erythrocyte traits. Mol. Genet. Genomic. Med 1 (3), 131–141. 24058921 10.1002/mgg3.16 PMC3775389

[R42] Hindley G, Bahrami S, Steen NE, O’Connell KS, Frei O, Shadrin A, Bettella F, Rødevand L, Fan CC, Dale AM, 2021. Characterising the shared genetic determinants of bipolar disorder, schizophrenia and risk-taking. Transl. Psychiatry 11 (1), 466. 34497263 10.1038/s41398-021-01576-4 PMC8426401

[R43] Hindley G, Drange OK, Lin A, Kutrolli G, Shadrin AA, Parker N, O’Connell KS, Rødevand L, Cheng W, Bahrami S, 2023. Cross-trait genome-wide association analysis of C-reactive protein level and psychiatric disorders. Psychoneuroendocrinology 157, 106368. 37659117 10.1016/j.psyneuen.2023.106368 PMC10802833

[R44] Holland D, Frei O, Desikan R, Fan C-C, Shadrin AA, Smeland OB, Sundar VS, Thompson P, Andreassen OA, Dale AM, 2020. Beyond SNP heritability: polygenicity and discoverability of phenotypes estimated with a univariate gaussian mixture model. PLoS Genet. 16 (5), e1008612. 32427991 10.1371/journal.pgen.1008612 PMC7272101

[R45] Howard DM, Adams MJ, Clarke T-K, Hafferty JD, Gibson J, Shirali M, Coleman JR, Hagenaars SP, Ward J, Wigmore EM, 2019. Genome-wide meta-analysis of depression identifies 102 independent variants and highlights the importance of the prefrontal brain regions. Nat. Neurosci 22 (3), 343–352. 30718901 10.1038/s41593-018-0326-7 PMC6522363

[R46] Howard DM, Adams MJ, Shirali M, Clarke T-K, Marioni RE, Davies G, Coleman JR, Alloza C, Shen X, Barbu MC, 2018. Genome-wide association study of depression phenotypes in UK Biobank identifies variants in excitatory synaptic pathways. Nat. Commun 9 (1), 1470. 30166530 10.1038/s41467-018-05310-5 PMC6117285

[R47] Howren MB, Lamkin DM, Suls J, 2009. Associations of depression with C-reactive protein, IL-1, and IL-6: a meta-analysis. Psychosom. Med 71 (2), 171–186. 19188531 10.1097/PSY.0b013e3181907c1b

[R48] James SL, Abate D, Abate KH, Abay SM, Abbafati C, Abbasi N, Abbastabar H, Abd-Allah F, Abdela J, Abdelalim A, 2018. Global, regional, and national incidence, prevalence, and years lived with disability for 354 diseases and injuries for 195 countries and territories, 1990–2017: a systematic analysis for the Global Burden of Disease Study 2017. The Lancet 392 (10159), 1789–1858. 10.1016/S0140-6736(18)32279-7 PMC6227754 30496104

[R49] Jongsma ML, de Waard AA, Raaben M, Zhang T, Cabukusta B, Platzer R, Blomen VA, Xagara A, Verkerk T, Bliss S, 2021. The SPPL3-defined glycosphingolipid repertoire orchestrates HLA class I-mediated immune responses. Immunity 54 (1), 132–150 e139. 33271119 10.1016/j.immuni.2020.11.003 PMC8722104

[R50] Karlović D, Serretti A, Vrkić N, Martinac M, Marčinko D, 2012. Serum concentrations of CRP, IL-6, TNF-α and cortisol in major depressive disorder with melancholic or atypical features. Psychiatry Res. 198 (1), 74–80. 22386567 10.1016/j.psychres.2011.12.007

[R51] Kircher M, Witten DM, Jain P, O’roak BJ, Cooper GM, Shendure J, 2014. A general framework for estimating the relative pathogenicity of human genetic variants. Nat. Genet 46 (3), 310–315. 24487276 10.1038/ng.2892 PMC3992975

[R52] Kishimoto T., 2010. IL-6: from its discovery to clinical applications. Int. Immunol 22 (5), 347–352. 20410258 10.1093/intimm/dxq030

[R53] Kovats S., 2015. Estrogen receptors regulate innate immune cells and signaling pathways. Cell Immunol. 294 (2), 63–69. 25682174 10.1016/j.cellimm.2015.01.018 PMC4380804

[R54] Lamers F, Milaneschi Y, Smit JH, Schoevers RA, Wittenberg G, Penninx BW, 2019. Longitudinal association between depression and inflammatory markers: results from the Netherlands study of depression and anxiety. Biol. Psychiatry 85 (10), 829–837. 30819515 10.1016/j.biopsych.2018.12.020

[R55] Levey DF, Stein MB, Wendt FR, Pathak GA, Zhou H, Aslan M, Quaden R, Harrington KM, Nuñez YZ, Overstreet C, 2021. Bi-ancestral depression GWAS in the Million Veteran Program and meta-analysis in>1.2 million individuals highlight new therapeutic directions. Nat. Neurosci 24 (7), 954–963. 34045744 10.1038/s41593-021-00860-2 PMC8404304

[R56] Li Y, Xiao B, Qiu W, Yang L, Hu B, Tian X, Yang H, 2010. Altered expression of CD4+ CD25+ regulatory T cells and its 5-HT1a receptor in patients with major depression disorder. J. Affect. Disord 124 (1-2), 68–75. 19900711 10.1016/j.jad.2009.10.018

[R57] Liu Z-Z, Wang Z-L, Choi T-I, Huang W-T, Wang H-T, Han Y-Y, Zhu L-Y, Kim H-T, Choi J-H, Lee J-S, 2018. Chd7 is critical for early T-cell development and thymus organogenesis in zebrafish. Am. J. Pathol 188 (4), 1043–1058. 29353058 10.1016/j.ajpath.2017.12.005

[R58] Lonsdale J, Thomas J, Salvatore M, Phillips R, Lo E, Shad S, Hasz R, Walters G, Garcia F, Young N, 2013. The genotype-tissue expression (GTEx) project. Nat. Genet 45 (6), 580–585. 23715323 10.1038/ng.2653 PMC4010069

[R59] Lynall M-E, Turner L, Bhatti J, Cavanagh J, de Boer P, Mondelli V, Jones D, Drevets WC, Cowen P, Harrison NA, 2020. Peripheral blood cell–stratified subgroups of inflamed depression. Biol. Psychiatry 88 (2), 185–196. 32000983 10.1016/j.biopsych.2019.11.017

[R60] MacKeigan JP, Murphy LO, Blenis J, 2005. Sensitized RNAi screen of human kinases and phosphatases identifies new regulators of apoptosis and chemoresistance. Nat. Cell Biol 7 (6), 591–600. 15864305 10.1038/ncb1258

[R61] Maes M, Van der Planken M, Stevens WJ, Peeters D, DeClerck L, Bridts C, Schotte C, Cosyns P, 1992. Leukocytosis, monocytosis and neutrophilia: hallmarks of severe depression. J. Psychiatr. Res 26 (2), 125–134. 1613679 10.1016/0022-3956(92)90004-8

[R62] Magnus P, Birke C, Vejrup K, Haugan A, Alsaker E, Daltveit AK, Handal M, Haugen M, Høiseth G, Knudsen GP, 2016. Cohort profile update: the Norwegian mother and child cohort study (MoBa). Int. J. Epidemiol 45 (2), 382–388. 27063603 10.1093/ije/dyw029

[R63] Mann JJ, Apter A, Bertolote J, Beautrais A, Currier D, Haas A, Hegerl U, Lonnqvist J, Malone K, Marusic A, 2005. Suicide prevention strategies: a systematic review. JAMa 294 (16), 2064–2074. 16249421 10.1001/jama.294.16.2064

[R64] Masopust D, Schenkel JM, 2013. The integration of T cell migration, differentiation and function. Nat. Rev. Immunol 13 (5), 309–320. 23598650 10.1038/nri3442

[R65] Medina-Rodriguez EM, Lowell JA, Worthen RJ, Syed SA, Beurel E, 2018. Involvement of innate and adaptive immune systems alterations in the pathophysiology and treatment of depression. Front. Neurosci 12, 547. 30174579 10.3389/fnins.2018.00547 PMC6107705

[R66] Miller AH, Maletic V, Raison CL, 2009. Inflammation and its discontents: the role of cytokines in the pathophysiology of major depression. Biol. Psychiatry 65 (9), 732–741. 19150053 10.1016/j.biopsych.2008.11.029 PMC2680424

[R67] Miller AH, Raison CL, 2016. The role of inflammation in depression: from evolutionary imperative to modern treatment target. Nat. Rev. Immunol 16 (1), 22–34. 26711676 10.1038/nri.2015.5 PMC5542678

[R68] Murray GK, Lin T, Austin J, McGrath JJ, Hickie IB, Wray NR, 2021. Could polygenic risk scores be useful in psychiatry?: a review. JAMA Psychiatry 78 (2), 210–219. 33052393 10.1001/jamapsychiatry.2020.3042

[R69] Nishimoto N, Kishimoto T, 2006. Interleukin 6: from bench to bedside. Nat. Clin. Pract. Rheumatol 2 (11), 619–626. 17075601 10.1038/ncprheum0338

[R70] O’Connell KS, Shadrin A, Bahrami S, Smeland OB, Bettella F, Frei O, Krull F, Askeland RB, Walters GB, Davíðsdóttir K, 2019. Identification of genetic overlap and novel risk loci for attention-deficit/hyperactivity disorder and bipolar disorder. Mol. Psychiatry 1–11. 10.1038/s41380-019-0613-z 31792363

[R71] Paltiel L, Anita H, Skjerden T, Harbak K, Bækken S, Kristin SN, Knudsen GP, Magnus P, 2014. The biobank of the Norwegian Mother and Child Cohort Study–present status. Nor. Epidemiol 24 (1-2).

[R72] Parker N, Cheng W, Hindley GF, O’Connell KS, Karthikeyan S, Holen B, Shadrin AA, Rahman Z, Karadag N, Bahrami S, 2024a. Genetic overlap between global cortical brain structure, C-reactive protein, and white blood cell counts. Biol. Psychiatry 95 (1), 62–71. 37348803 10.1016/j.biopsych.2023.06.008 PMC11684752

[R73] Parker N, Koch E, Shadrin AA, Fuhrer J, Hindley GFL, Stinson S, Jaholkowski P, Tesfaye M, Dale AM, Wingo TS, Wingo AP, Frei O, O’Connell KS, Smeland OB, Andreassen OA, 2024b. Leveraging the genetics of psychiatric disorders to prioritize potential drug targets and compounds. medRxiv, 2024.2009.2024.24314069. 10.1038/s41386-026-02380-8 PMC13212996 41795042

[R74] Paul S, Dansithong W, Gandelman M, Figueroa KP, Zu T, Ranum LP, Scoles DR, Pulst SM, 2023. Staufen impairs autophagy in neurodegeneration. Ann. Neurol 93 (2), 398–416. 36151701 10.1002/ana.26515 PMC9892312

[R75] Reddy NC, Majidi SP, Kong L, Nemera M, Ferguson CJ, Moore M, Goncalves TM, Liu H-K, Fitzpatrick JA, Zhao G, 2021. CHARGE syndrome protein CHD7 regulates epigenomic activation of enhancers in granule cell precursors and gyrification of the cerebellum. Nat. Commun 12 (1), 5702. 34588434 10.1038/s41467-021-25846-3 PMC8481233

[R76] Romero C, Werme J, Jansen PR, Gelernter J, Stein MB, Levey D, Polimanti R, de Leeuw C, Posthuma D, Nagel M, 2022. Exploring the genetic overlap between twelve psychiatric disorders. Nat. Genet 1–8. 36471075 10.1038/s41588-022-01245-2

[R77] Scariano JK, Emery-Cohen AJ, Pickett GG, Morgan M, Simons PC, Alba F, 2008. Estrogen receptors alpha (ESR1) and beta (ESR2) are expressed in circulating human lymphocytes. J. Recept. Signal Transduct 28 (3), 285–293. 10.1080/10799890802084614 18569528

[R78] Schwartzman A, Lin X, 2011. The effect of correlation in false discovery rate estimation. Biometrika 98 (1), 199–214. 23049127 10.1093/biomet/asq075 PMC3412603

[R79] Sealock JM, Lee YH, Moscati A, Venkatesh S, Voloudakis G, Straub P, Singh K, Feng Y-CA, Ge T, Roussos P, 2021. Use of the PsycheMERGE network to investigate the association between depression polygenic scores and white blood cell count. JAMA Psychiatry 78 (12), 1365–1374. 34668925 10.1001/jamapsychiatry.2021.2959 PMC8529528

[R80] Seidel A, Arolt V, Hunstiger M, Rink L, Behnisch A, Kirchner H, 1996. Major depressive disorder is associated with elevated monocyte counts. Acta Psychiatr. Scand 94 (3), 198–204. 8891088 10.1111/j.1600-0447.1996.tb09849.x

[R81] Shen N, Korm S, Karantanos T, Li D, Zhang X, Ritou E, Xu H, Lam A, English J, Zong W-X, 2021. DLST-dependence dictates metabolic heterogeneity in TCA-cycle usage among triple-negative breast cancer. Commun. Biol 4 (1), 1289. 34785772 10.1038/s42003-021-02805-8 PMC8595664

[R82] Simon MS, Arteaga-Henríquez G, Fouad Algendy A, Siepmann T, Illigens BM, 2023. Anti-inflammatory treatment efficacy in major depressive disorder: a systematic review of meta-analyses. Neuropsychiatr. Dis. Treat 1–25. 36636142 10.2147/NDT.S385117 PMC9830720

[R83] Smeland OB, Andreassen OA, 2021. Polygenic risk scores in psychiatry–Large potential but still limited clinical utility. Eur. Neuropsychopharmacol 51, 68–70. 34091254 10.1016/j.euroneuro.2021.05.007

[R84] Smeland OB, Bahrami S, Frei O, Shadrin A, O’Connell K, Savage J, Watanabe K, Krull F, Bettella F, Steen NE, 2020a. Genome-wide analysis reveals extensive genetic overlap between schizophrenia, bipolar disorder, and intelligence. Mol. Psychiatry 25 (4), 844–853. 30610197 10.1038/s41380-018-0332-x PMC6609490

[R85] Smeland OB, Frei O, Kauppi K, Hill WD, Li W, Wang Y, Krull F, Bettella F, Eriksen JA, Witoelar A, 2017. Identification of genetic loci jointly influencing schizophrenia risk and the cognitive traits of verbal-numerical reasoning, reaction time, and general cognitive function. JAMa Psychiatry 74 (10), 1065–1075. 28746715 10.1001/jamapsychiatry.2017.1986 PMC5710474

[R86] Smeland OB, Frei O, Shadrin A, O’Connell K, Fan C-C, Bahrami S, Holland D, Djurovic S, Thompson WK, Dale AM, 2020b. Discovery of shared genomic loci using the conditional false discovery rate approach. Hum. Genet 139 (1), 85–94. 31520123 10.1007/s00439-019-02060-2

[R87] Smeland OB, Kutrolli G, Bahrami S, Fominykh V, Parker N, Hindley GFL, Rødevand L, Jaholkowski P, Tesfaye M, Parekh P, Elvsåshagen T, Grotzinger AD, Consortium, T.I.M.S.G., Consortium, T.I.H.G., Steen NE, Meer D.v.d., O’Connell KS, Djurovic S, Dale AM, Shadrin AA, Frei O, Andreassen OA, 2023. The shared genetic risk architecture of neurological and psychiatric disorders: a genome-wide analysis. medRxiv, 2023.2007.2021.23292993.

[R88] Steen NE, Rahman Z, Szabo A, Hindley GF, Parker N, Cheng W, Lin A, O’Connell KS, Sheikh MA, Shadrin A, 2023. Shared genetic loci between schizophrenia and white blood cell counts suggest genetically determined systemic immune abnormalities. Schizophr. Bull sbad082. 10.1093/schbul/sbad082 PMC10483470 37319439

[R89] Sullivan PF, Neale MC, Kendler KS, 2000. Genetic epidemiology of major depression: review and meta-analysis. Am. J. Psychiatry 157 (10), 1552–1562. 11007705 10.1176/appi.ajp.157.10.1552

[R90] Tanaka T, Narazaki M, Kishimoto T, 2014. IL-6 in inflammation, immunity, and disease. Cold. Spring. Harb. Perspect. Biol 6 (10), a016295. 25190079 10.1101/cshperspect.a016295 PMC4176007

[R91] Vogelzangs N, Beekman AT, van Reedt Dortland AK, Schoevers RA, Giltay EJ, De Jonge P, Penninx BW, 2014. Inflammatory and metabolic dysregulation and the 2-year course of depressive disorders in antidepressant users. Neuropsychopharmacology 39 (7), 1624–1634. 24442097 10.1038/npp.2014.9 PMC4023159

[R92] Watanabe K, Taskesen E, Van Bochoven A, Posthuma D, 2017. Functional mapping and annotation of genetic associations with FUMA. Nat. Commun 8 (1), 1826. 29184056 10.1038/s41467-017-01261-5 PMC5705698

[R93] Wiström ED, O’Connell KS, Karadag N, Bahrami S, Hindley GF, Lin A, Cheng W, Steen NE, Shadrin A, Frei O, 2022. Genome-wide analysis reveals genetic overlap between alcohol use behaviours, schizophrenia and bipolar disorder and identifies novel shared risk loci. Addiction 117 (3), 600–610. 34472679 10.1111/add.15680

[R94] Wörns M, Victor A, Galle P, Höhler T, 2006. Genetic and environmental contributions to plasma C-reactive protein and interleukin-6 levels–a study in twins. Genes Immunity 7 (7), 600–605. 16900203 10.1038/sj.gene.6364330

[R95] Wray NR, Ripke S, Mattheisen M, Trzaskowski M, Byrne EM, Abdellaoui A, Adams MJ, Agerbo E, Air TM, Andlauer TM, 2018. Genome-wide association analyses identify 44 risk variants and refine the genetic architecture of major depression. Nat. Genet 50 (5), 668. 29700475 10.1038/s41588-018-0090-3 PMC5934326

[R96] Zhang Y, Sloan SA, Clarke LE, Caneda C, Plaza CA, Blumenthal PD, Vogel H, Steinberg GK, Edwards MS, Li G, 2016. Purification and characterization of progenitor and mature human astrocytes reveals transcriptional and functional differences with mouse. Neuron 89 (1), 37–53. 26687838 10.1016/j.neuron.2015.11.013 PMC4707064

