# Supplementary figures and images for: The genetic overlap between major depressive disorder, white blood cell counts and interleukin 6
Source: J Affect Disord Rep. Author manuscript; Available in PMC 2025 Nov 26. (PMC12646636; doi:10.1016/j.jadr.2025.100889)

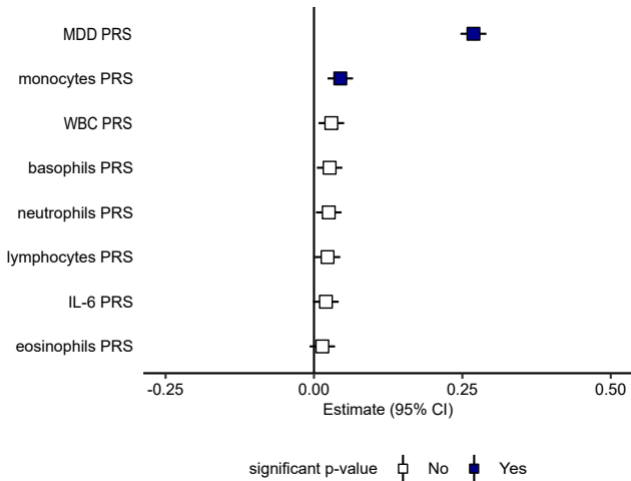

Supplement: 1 [file NIHMS2115889-supplement-1.zip › SUPPLEMENTARY FILES/depression_all_final_2024_02_14.pdf]

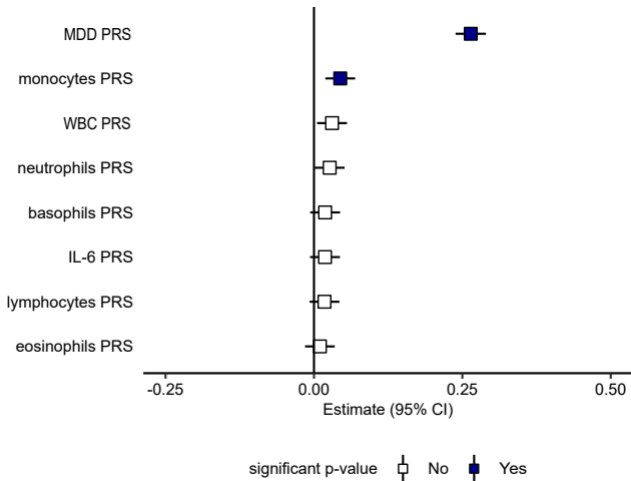

Supplement: 1 [file NIHMS2115889-supplement-1.zip › SUPPLEMENTARY FILES/depression_all__smoking_BMI_final2024_02_14.pdf]

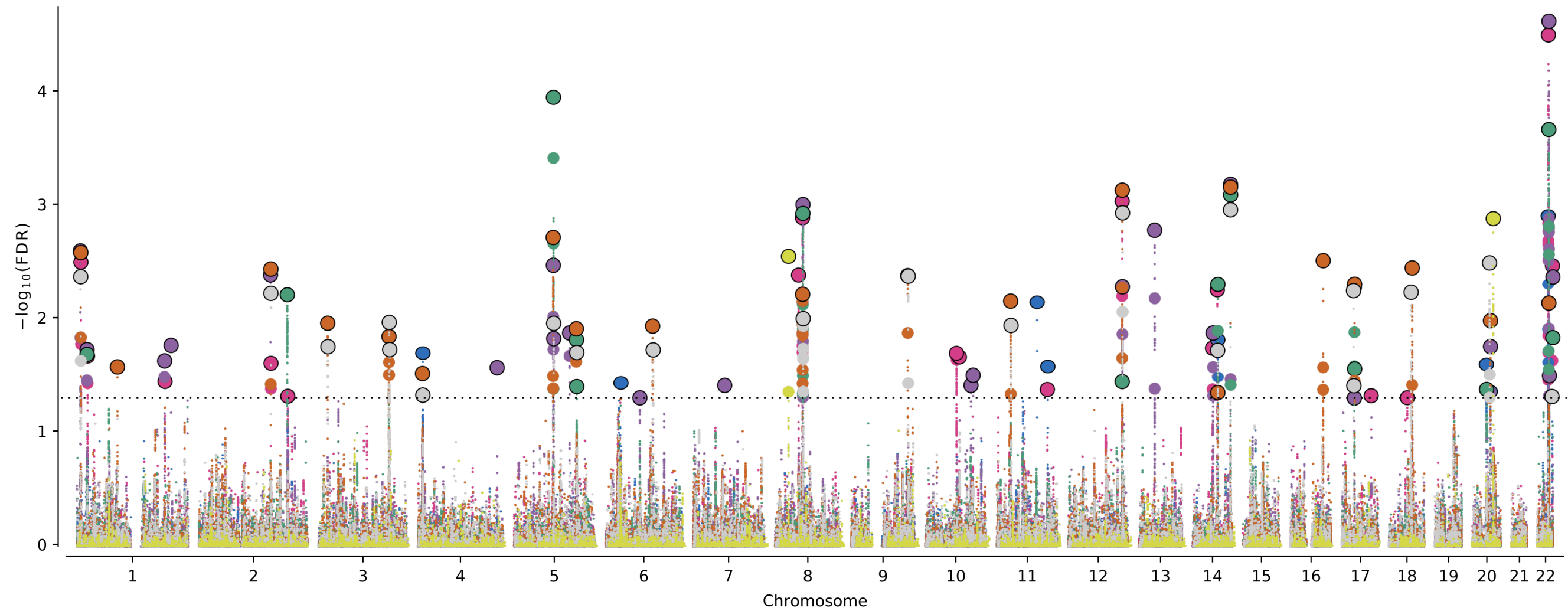

Supplement: 1 [file NIHMS2115889-supplement-1.zip › SUPPLEMENTARY FILES/Manhattan composite plot (1).pdf]

# MDD | Basophils

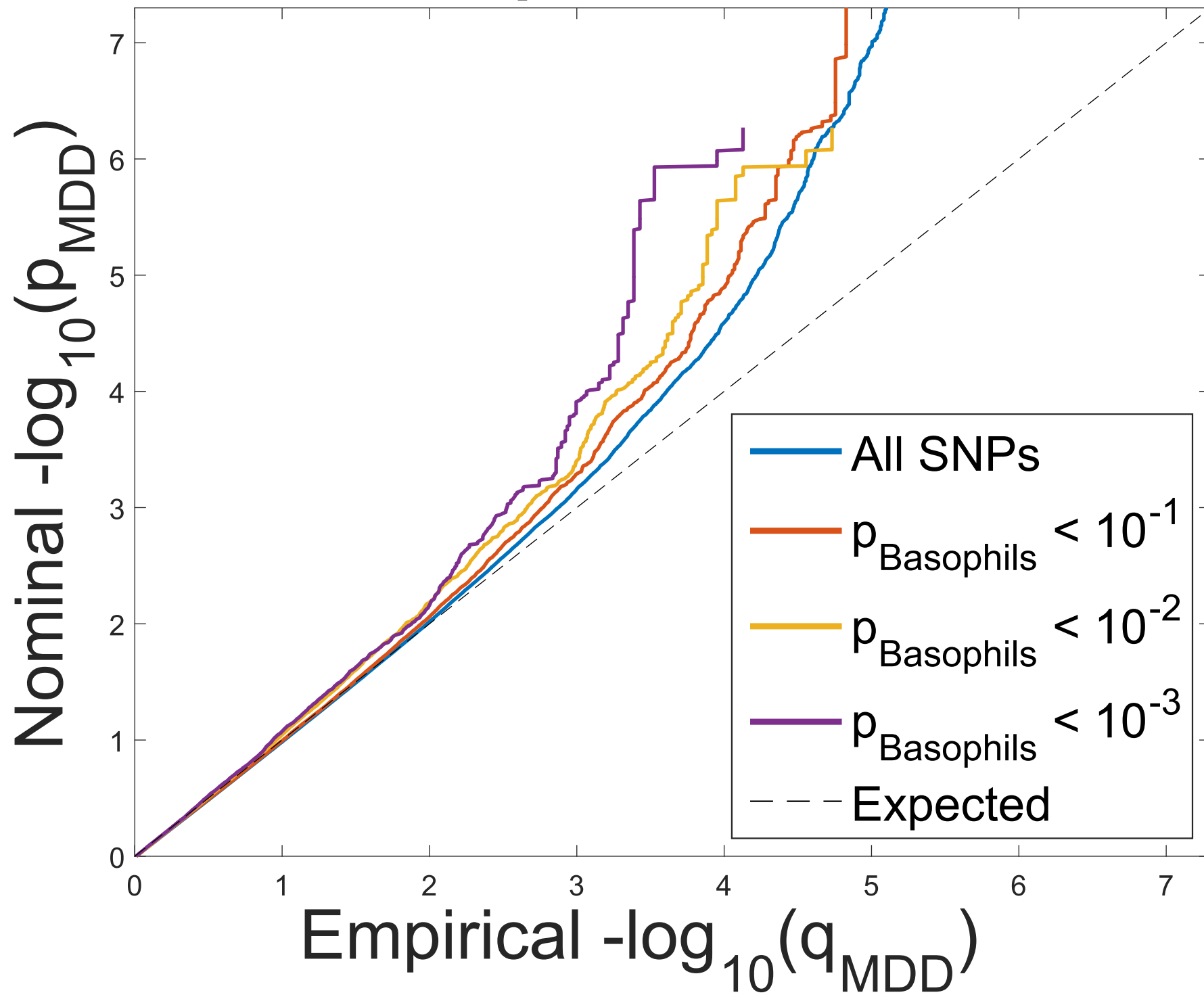

Supplement: 1 [file NIHMS2115889-supplement-1.zip › SUPPLEMENTARY FILES/MDD_vs_Basophils_qq01.pdf]

# MDD | Eosinophils

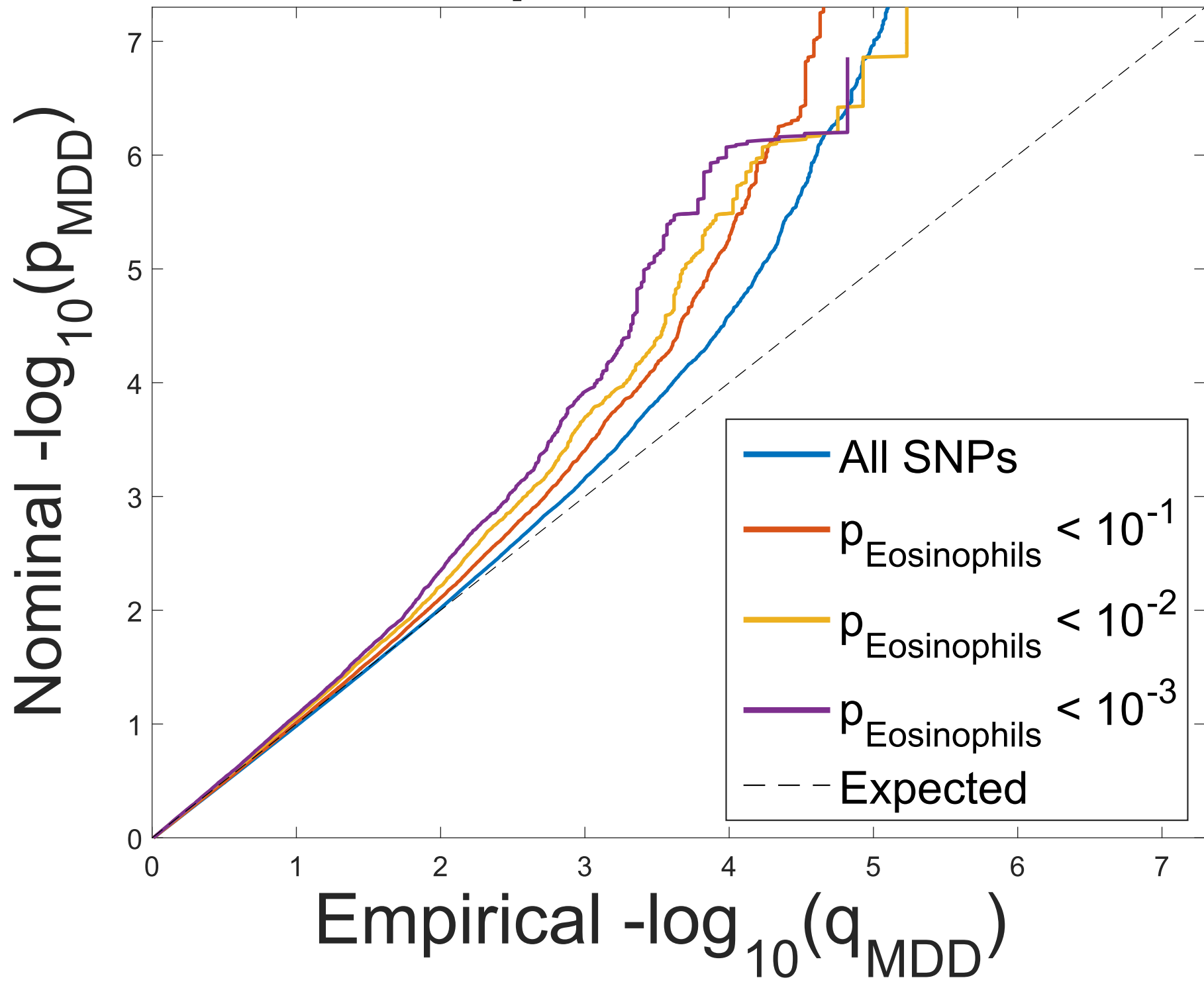

Supplement: 1 [file NIHMS2115889-supplement-1.zip › SUPPLEMENTARY FILES/MDD_vs_Eosinophils_qq01.pdf]

# MDD | IL-6

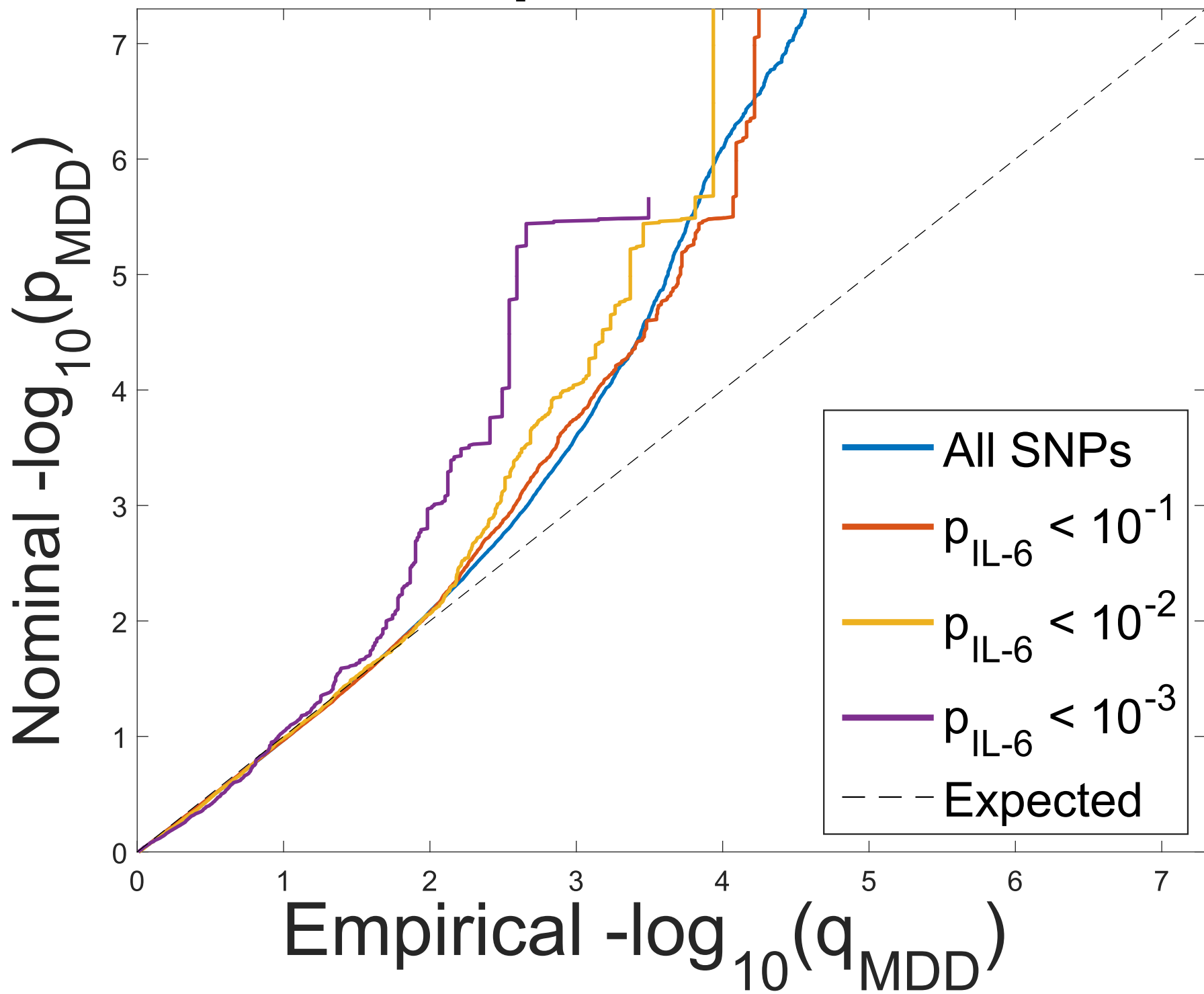

Supplement: 1 [file NIHMS2115889-supplement-1.zip › SUPPLEMENTARY FILES/MDD_vs_IL-6_01_qq.pdf]

# MDD | Lymphocytes

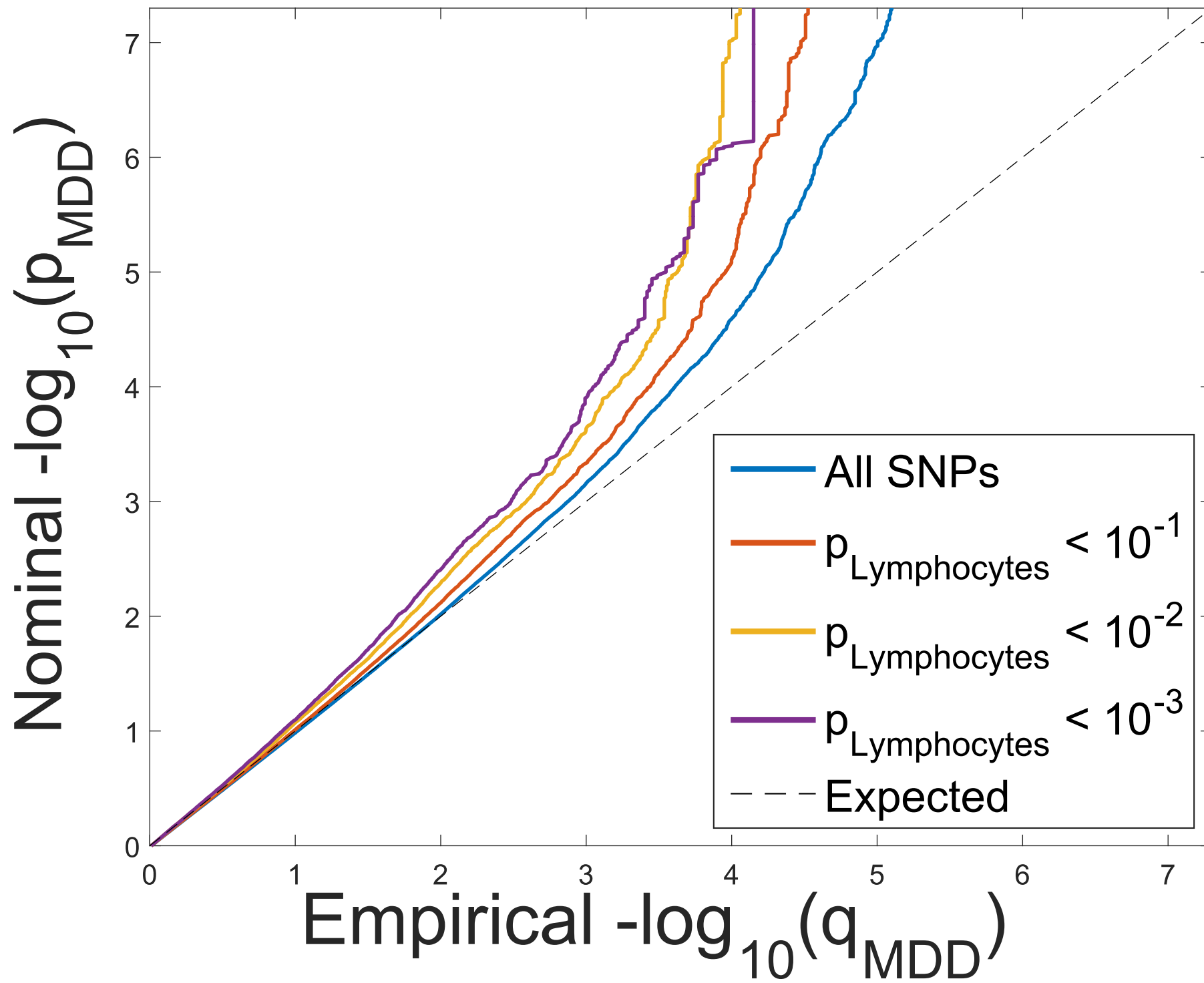

Supplement: 1 [file NIHMS2115889-supplement-1.zip › SUPPLEMENTARY FILES/MDD_vs_Lymphocytes_qq.pdf]

# MDD | Monocytes

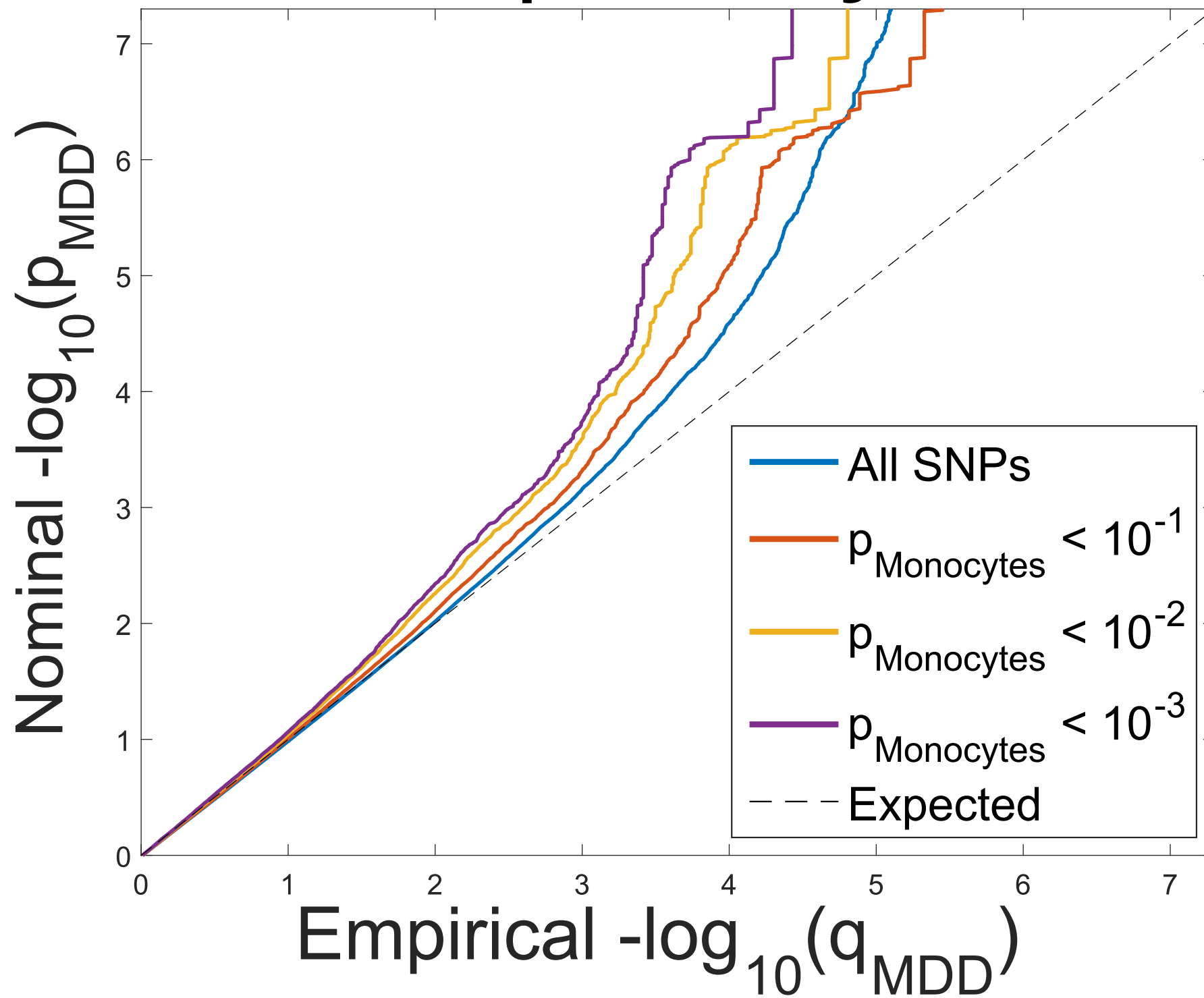

Supplement: 1 [file NIHMS2115889-supplement-1.zip › SUPPLEMENTARY FILES/MDD_vs_Monocytes_qq01.pdf]

# MDD | Neutrophils

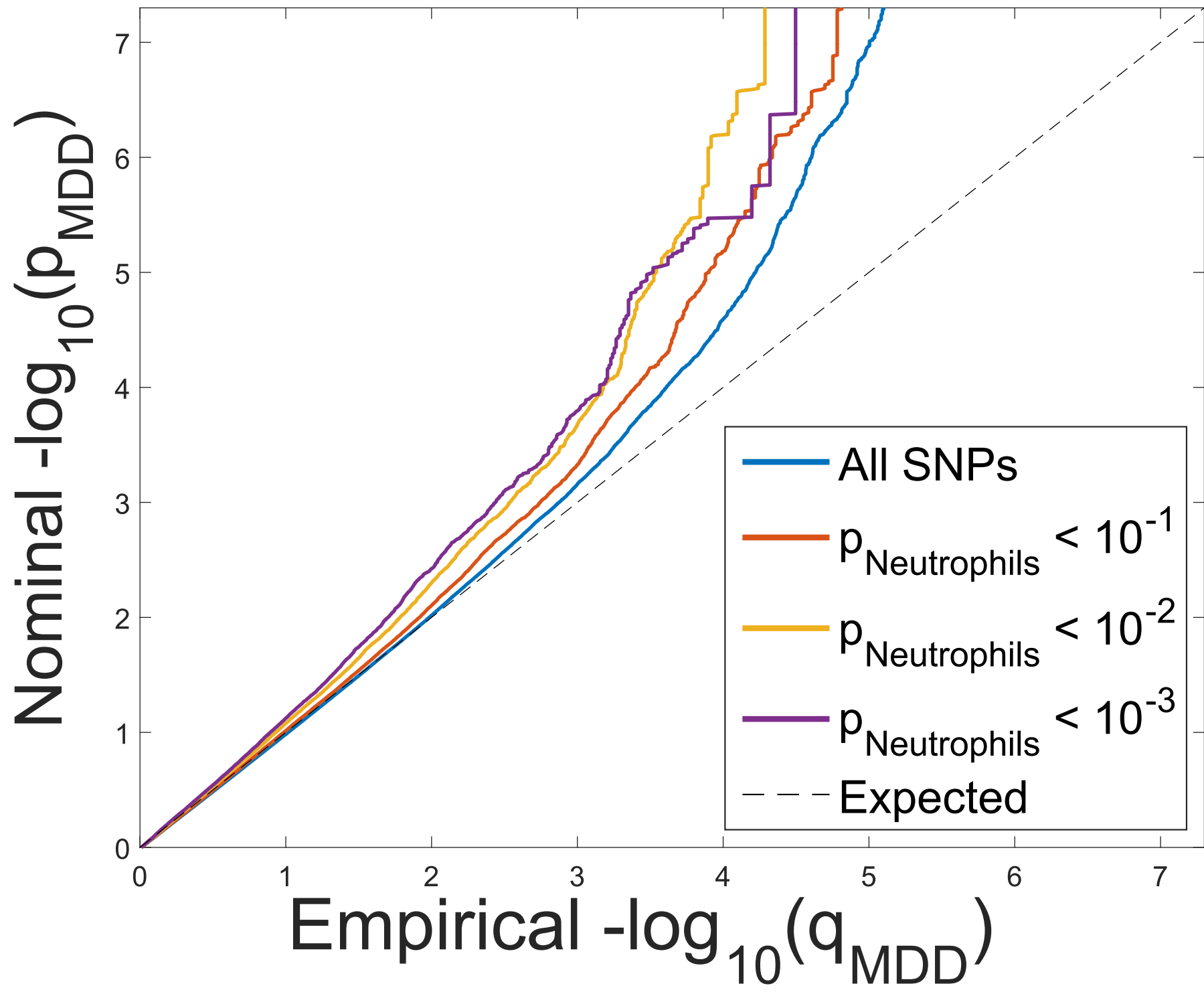

Supplement: 1 [file NIHMS2115889-supplement-1.zip › SUPPLEMENTARY FILES/MDD_vs_Neutrophils_qq01.pdf]

# MDD | Total WBC

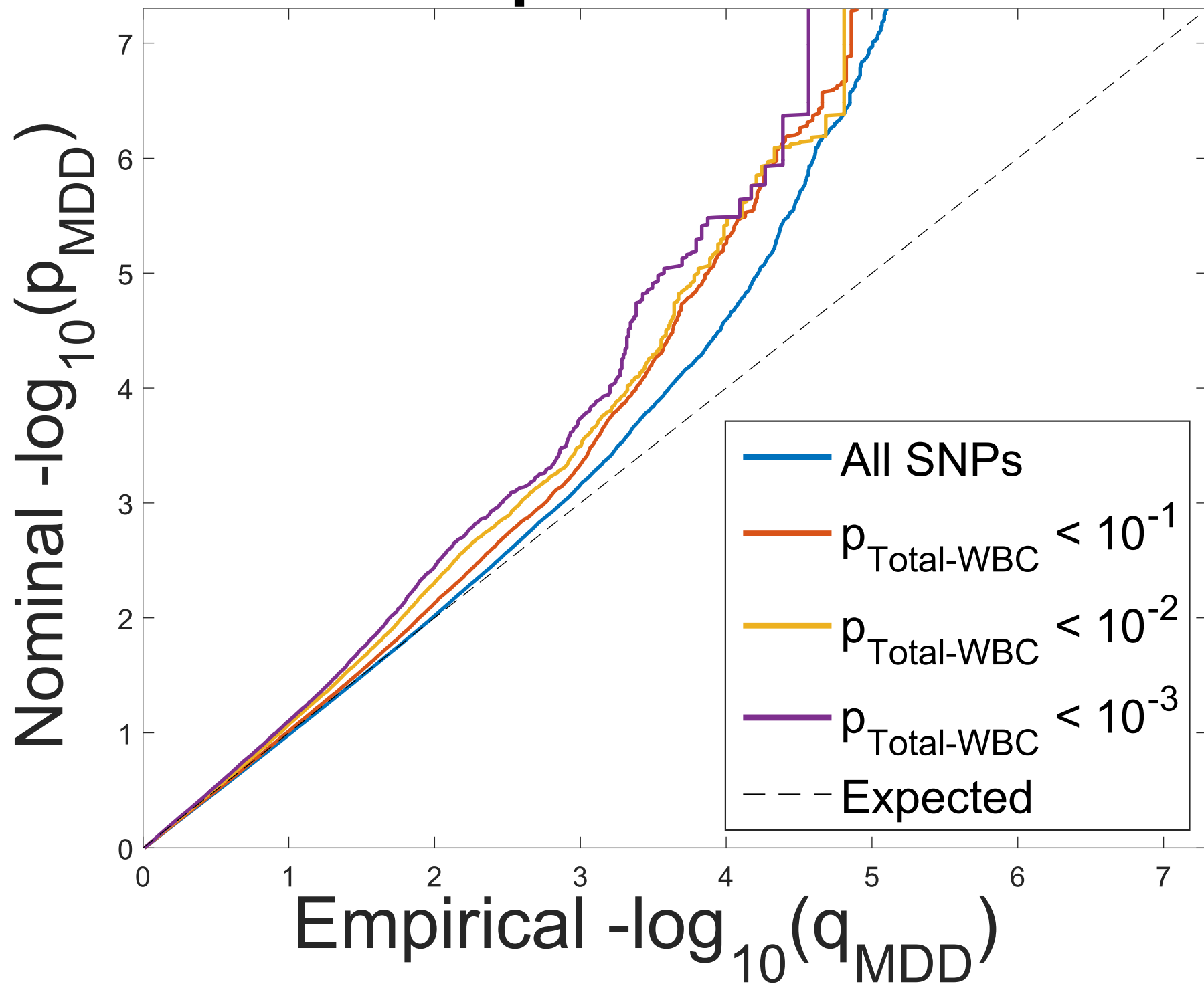

Supplement: 1 [file NIHMS2115889-supplement-1.zip › SUPPLEMENTARY FILES/MDD_vs_Total-WBC_qq01.pdf]

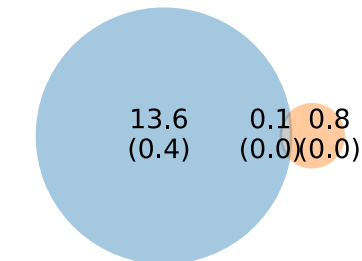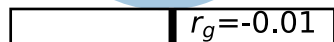

MDD & Basophils

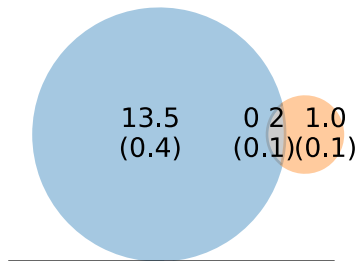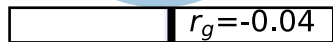

MDD & Eosinophils

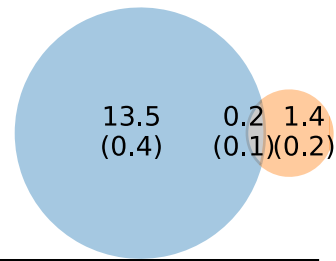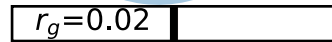

MDD & Lymphocytes

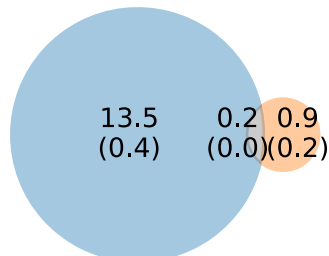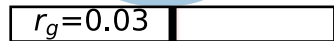

MDD & Monocytes

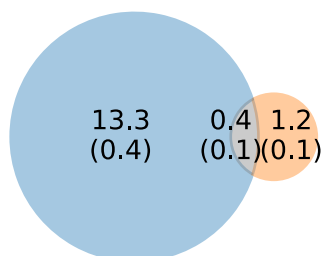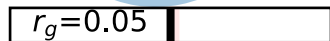

MDD & Neutrophils

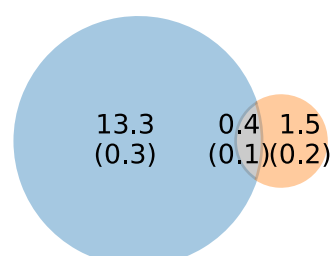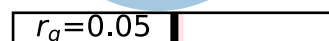

MDD & Total WBC

Supplement: 1 [file NIHMS2115889-supplement-1.zip › SUPPLEMENTARY FILES/MiXeR.pdf]
