# Supplementary material for: The genetic overlap between major depressive disorder, white blood cell counts and interleukin 6
Source: J Affect Disord Rep. Author manuscript; Available in PMC 2025 Nov 26. (PMC12646636; doi:10.1016/j.jadr.2025.100889)
Supplement: 3 [file NIHMS2115889-supplement-3.docx]

**Supplementary Methods**

Genome Wide Analysis Identifies Patterns of Shared Genetic Architecture and Overlapping Loci Between Major depressive disorder, White Blood Cell Counts and Interleukin 6

**Genome-wide association study summary datasets**

Major depressive disorder

Summary statistics on major depressive disorder (MDD) were obtained from a large-scale genome-wide association study (GWAS) published in 2021 (*n* cases=330,173 and *n* controls=727,595)(Levey et al., 2021). The definition of the MDD phenotype varied across the individual studies included in the GWAS. The broadest definition was applied in the UK Biobank sample, determined by a positive response to questions of whether the participants ever had seen a general practitioner or psychiatrist for nerves, anxiety, tension, or depression. In some cohorts, the phenotype was defined by patient self-reporting having received a clinical diagnosis or treatment of depression, while in other cohorts the diagnosis was determined by electronic health records. See the original GWAS for detailed descriptions (Howard et al., 2019; Levey et al., 2021). The GWAS analysis was carried out in MVP covarying for age, sex and the first 10 principal components (PCs), in UKB covarying for age, sex, genotyping array, and the first 8 principal components, while in 23andMe covaried for age, sex and the first five PCs (Howard et al., 2019; Hyde et al., 2016; Levey et al., 2021).

White blood cells

Summary statistics on white blood cells (WBC) were obtained from a GWAS study published be Chen et al. in 2020 (*n*_max_=563,946). For detailed account on the number of participants for each WBC subgroup, see the original publication (Chen et al., 2020). Individuals with potential blood cancer, hereditary amnesia, HIV and other serious conditions affecting blood count were excluded along with pregnant women (Chen et al., 2020). Additionally, individuals with extreme WBC measures exceeding 200x10^9^ cells/L were excluded. For more details, see the original publication (Chen et al., 2020). The phenotypes were corrected for sex, age, age-squared, the first 10 principal components and other study-specific covariates. The imputation and quality control varied among different sub-studies, as detailed in the original GWAS (Chen et al., 2020). For most studies, samples were removed if the genotyping call rate was <95%, if there were indications of excess heterozygosity, if there were gender mismatches, sample duplicates, or if they appeared as population outliers in principal component analyses. Monomorphic variants and variants with Hardy-Weinberg *p*<1×10^−6^ and call rate <98% were also removed.

Interleukin 6

Summary statistics on interleukin 6 (Il-6) was obtained from a GWAS published in 2021 (*n*=52,654). Each cohort collected venous blood samples. Measurements with extreme distribution (mean ±4 standard deviations) were excluded. Participating cohorts conducted assessments for missingness and evaluated for indices of quality control. Substudies utilised various genotyping platforms for genome-wide genotyping and adhered to predefined quality control of genotype data. Imputation of non-genotyped genetic variants was carried out with the assistance of the Hapmap Phase II reference panel, and statistical software such as Impute, Minimac, MACH or BIMBAM. For more details on quality control and imputation, see the original GWAS (Ahluwalia et al., 2021).

**Statistical analytical tools**

MiXeR analysis

MiXeR (v1.3) constructs causal mixture models from GWAS summary statistics (<https://github.com/precimed/mixer>) (Frei et al., 2019; Holland et al., 2020; Karadag et al., 2023). Initially, MiXeR performs a univariate analysis by modelling the additive genetic effect of allele substitution, 𝛽_𝑖_ , for each SNP, 𝑖, as a point normal mixture, 𝛽_𝑖_ = (1 − 𝜋_1_ )𝑁(0,0) + 𝜋_1_𝑁(0, $\sigma_{\beta}^{2}$ ), whereby 𝜋_1_ is the proportion of trait influencing SNPs (i.e. “causal” SNPs/non-null SNPs/SNPs with true genetic effect beyond linkage disequilibrium), referred to as ‘polygenicity’, and $\sigma_{\beta}^{2}$ is the variance of effect sizes of “trait-influencing” SNPs, referred to as ‘discoverability'. Subsequently, MiXeR combines LD data and allele frequencies for 9,997,231 SNPs from the 1000 Genomes Phase3 data for each SNP, 𝑗 (Bulik-Sullivan et al., 2015). Using this information, the expected probability distribution of the signed test statistic is computed as 𝑧_𝑗_ = 𝛿_𝑗_ + 𝜖_𝑗_ = 𝑁 ∑_𝑖_ $\sqrt{H_{i}}$𝑟_𝑖𝑗_𝛽_𝑖_ + 𝜖_𝑗_ , in which 𝑁 represents sample size, 𝐻_𝑖_ represents the heterozygosity of SNP i, 𝑟_𝑖𝑗_ represents allelic correlation between SNP i and j, and 𝜖_𝑗_ ∼ 𝑁(0, $\sigma_{0}^{2}$) represents residual variance. Direct maximization of the likelihood function is used to fit the three parameters, 𝜋_1_, $\sigma_{\beta}^{2}$ , $\sigma_{0}^{2}$ . The number of “trait-influencing” variants is finally computed as 𝑀𝜋_1_, in which M = the number of SNPs within the LD reference panel. SNP-based heritability was estimated on the observed scale. All point estimates and standard deviations were computed by conducting 20 iterations with 2 million random SNPs followed by random pruning at an r^2^ threshold of 0.8, (i.e. ~600K SNPs per iteration).

To evaluate model fit, the Akaike information criterion (𝐴𝐼𝐶 = 2𝑘 − 2𝑙𝑛𝐿) was used, in which 𝑘 represents the number of free parameters, 𝐿 represents the value of the likelihood function, and 𝑛 represents the number of SNPs used in the optimization procedure. A positive AIC value indicates that the model fits the data better than the infinitesimal model.

Conditional Q-Q plots and cross-trait enrichment

Under large-scale testing paradigms, such as GWAS, quantitative estimates of likely true associations can be obtained from the distributions of summary statistics (Efron, 2007; Schweder and Spjøtvoll, 1982). A widely used method for visualizing the enrichment of statistical association relative to that expected under the global null hypothesis is through Q-Q plots of nominal p-values obtained from GWAS summary statistics. The Q-Q curve has as the y-ordinate the nominal p-value, denoted by “p”, and as the x-ordinate the corresponding value of the empirical cdf, denoted by “q”. Under the global null hypothesis, the theoretical distribution is uniform on the interval [0,1]. In the presence of all null relationships, nominal p-values form a straight line on a Q-Q plot when plotted against the empirical distribution. Leftward deflections of the observed distribution from the projected null line reflect increased tail probabilities in the distribution of test statistics (z-scores) and consequently an over-abundance of low p-values compared to that expected by chance, also named ‘enrichment’. To emphasize tail probabilities of the theoretical and empirical distributions, the log_10_ p is commonly plotted against the -log_10_ q.

Conditional Q-Q plots are constructed by creating subsets of SNPs based on levels of an auxiliary measure for each SNP, and computing Q-Q plots separately for each level (Smeland et al., 2020). If SNP enrichment is captured by variation in the auxiliary measure, this is expressed as successive leftward deflections in a conditional Q-Q plot as levels of the auxiliary measure increase. The enrichment can be directly interpreted in terms of the true discovery rate (1−FDR) (see below) (Efron, 2010). Cross-trait enrichment exists if the proportion of SNPs associated with a phenotype increases as a function of the strength of the association with a secondary phenotype. We constructed conditional Q-Q plots of empirical quantiles of nominal -log_10_ p-values for SNP association for all SNPs, and for subsets (strata) of SNPs determined by the nominal p-values of their association with the conditional phenotypes, and vice versa. Specifically, we computed the empirical cumulative distribution of nominal p-values for a given phenotype for all SNPs and for SNPs with significance levels below the indicated cut-offs for the conditional phenotypes (-log_10_(p) ≥ 1, -log_10_(p) ≥ 2, log_10_(p) ≥ 3 corresponding to p < 0.1, p < 0.01, p < 0.001 respectively). The nominal p-values (– log_10_(p)) are plotted on the y-axis, and the empirical quantiles (–log_10_(q), where q=1-cdf(p)) are plotted on the x-axis. To assess for polygenic effects below the standard GWAS significance threshold, we focused the conditional Q-Q plots on SNPs with nominal –log_10_(p) < 7.3 (corresponding to p > 5x10-^8^) (Andreassen et al., 2013b).

Detection of genetic variants using conjunctional FDR

The FDR can be interpreted as the probability that a SNP is null given that its p-value is as small as or smaller than its observed p-value. The condFDR is an extension of the standard FDR, which incorporates information from GWAS summary statistics of a second phenotype to adjust its significance level. The condFDR is defined as the probability that a SNP is null in the first phenotype given that the p-values in the first and second phenotypes are as small as or smaller than the observed ones. The condFDR estimates are obtained for each nominal SNP p-value in the primary phenotype after computing the stratified empirical cumulative distribution functions (cdfs) of the nominal p-values (Sun et al., 2006; Yoo et al., 2009). The separate strata are determined by the relative enrichment of SNP associations as a function of increased nominal SNP p-values in a secondary phenotype.

The standard FDR framework stems from a model that assumes that the distribution of test statistics in a GWAS can be formulated as a mixture of null and non-null effects, with true associations having more extreme test statistics than false associations on average. Ranking SNPs by the standard FDR or by p-values gives the same ordering of SNPs. In contrast, if the primary and secondary phenotypes are related genetically, the condFDR reorders SNPs and results in a different ranking than that based solely on p-values. The conjFDR is defined as the posterior probability that a SNP is null for either phenotype or both simultaneously, given that its p-values for association with both phenotypes are as small as or smaller than the observed p-values (Andreassen et al., 2015a; Andreassen et al., 2013a; Andreassen et al., 2015b; Andreassen et al., 2014; Andreassen et al., 2013b). A conservative estimate of the conjFDR is given by the maximum condFDR for a given SNP after repeating the condFDR procedure for both traits and inverting their roles (Smeland et al., 2020). If summary statistics for the same SNP were not available in both of the discovery datasets, overlapping SNP associations could not be assessed using conjFDR analysis (Wiström et al., 2022).

Conditional and conjunctional false discovery rate

The ‘enrichment’ seen in conditional Q-Q plots can be directly interpreted in terms of a Bayesian true discovery rate (1 – FDR) (Andreassen et al., 2013a; Efron, 2010). More specifically, for a given p-value, under a simple two-group (null and non-null) model, Bayes’ rule gives the posterior probability of being null as

FDR(p) = π_0_F_0_ (p) / F(p), [1]

where π_0_ is the proportion of null SNPs, F_0_ is the CDF of p-values under the null hypothesis *H_0_* (no genetic associations with the phenotype), and F is the CDF of the distribution of p-values of all SNPs, both null and non-null (Efron, 2007). Here, we assume the SNP p-values are independent and identically distributed. Under the null hypothesis, F_0_ is the CDF of the uniform distribution on the unit interval [0,1], so that Eq. [1] reduces to

FDR(p) = π_0_ p / F(p). [2]

for a given p-value p. F can be estimated by the empirical CDF q = N_p_ / Ν, where N_p_ is the number of SNPs with p-values less than or equal to p, and N is the total number of SNPs. Replacing F by q in Eq. [2], we get

Estimated FDR(p) = π_0_ p / q, [3]

which is biased upwards as an estimate of the FDR (Efron and Tibshirani, 2002). Replacing π_0_ in Equation [3] with unity gives an estimated FDR that is further biased upwards and defined as

q* = p/q. [4]

If π_0_ is close to one, as is likely true for most GWASs, the increase in bias from Eq. [3] is minimal.

The quantity 1 – p/q, is therefore biased downwards, and hence a conservative estimate of the TDR. Referring to the Q-Q plots, we see that q* is equivalent to the nominal p-value divided by the empirical quantile, as defined earlier. We can thus read the FDR estimate directly off the Q-Q plot as

-log_10_(q*) = log_10_(q) – log_10_(p), [5]

i.e., the horizontal shift of the curves in the Q-Q plots from the expected line x = y, with a larger shift corresponding to a smaller FDR. This shift is illustrated in Figure 1. To estimate the conditional FDR (condFDR) of a given SNP, we repeat the above procedure for a subset of SNPs with p-values in the secondary GWAS equal to or lower than that observed for the given SNP. Formally, this is given by

FDR_Phenotype1|Phenotype2_ = condFDR(p_1_,p_2_) = π_0_ (p_2_)p_1_/ F(p_1_|p_2_), [6]

where p_1_ is the p-value for the first phenotype, p_2_ is the p-value for the second, F(p_1_ | p_2_) is the conditional CDF, and π_0_ (p_2_) the conditional proportion of null SNPs for the first phenotype given that p-values for the second phenotype are less than or equal to p_2_. For a given SNP condFDR thus represents the estimated posterior probability of no-association with the primary phenotype given its p-values for both primary and secondary phenotypes. The condFDR framework is closely related to the stratified FDR method developed by Sun et al. (Sun et al., 2006). Whereas they propose computing FDR separately conditional on membership in pre-defined discrete strata of p-values, here, we condition the estimated FDR on a continuous random variable, the SNP p-values with respect to a second phenotype.

To identify SNPs jointly associated with two phenotypes using conjunctional FDR (conjFDR), the condFDR procedure is repeated after inverting the roles of the primary and secondary phenotypes. Similar to previous conjunction tests for p-value statistics (Nichols et al., 2005), the conjFDR estimate is defined as the maximum of both condFDR values, which minimizes the effect of a single phenotype driving the common association signal. Formally, for a given SNP with p-values for association with the first and the second phenotype equal to p_1_ and p_2_ respectively, the conjFDR is given by

FDR_Phenotype1&Phenotype2_ = conjFDR (p_1_, p_2_) = π_0_ F_0_(p_1_, p_2_) / F(p_1_, p_2_) + π_1_ F_1_(p_1_, p_2_) / F(p_1_, p_2_) + π_2_ F_2_(p_1_, p_2_) / F(p_1_, p_2_), [7]

where π_0_ is the *a priori* proportion of SNPs null for both phenotypes simultaneously and F_0_(p_1_, p_2_) is the joint null CDF, π_1_ is the *a priori* proportion of SNPs non-null for the first phenotype and null for the second with F_1_(p_1_, p_2_) the joint CDF of p-value distribution of these SNPs, and π_2_ is the *a priori* proportion of SNPs non-null for the second phenotype and null for the first, with joint CDF of p-value distribution of these SNPs F_2_(p_1_, p_2_). F(p_1_, p_2_) is the joint CDF of p-value distribution of all SNPs for phenotype 1 and 2. ConjFDR thus represents the posterior probability that a given SNP is null for either phenotype or both phenotypes simultaneously when the p-values for both phenotypes are as small or smaller than the observed p-values.

Conditional empirical CDFs provide a model-free method to obtain conservative estimates of Eq (7). This can be seen as follows. For a given SNP, estimate the conjFDR by

Estimated FDR_Phenotype1&Phenotype2 =_

max {Estimated FDR_Phenotype1|Phenotype2_, Estimated FDR_Phenotype2|Phenotype1_}, [8]

where Estimated FDR_Phenotype1|Phenotype2_ and Estimated FDR_Phenotype2|Phenotype1_ are conservative (upwards biased) estimates of Eq. [6]. Thus, Eq (8) is a conservative estimate of max {p_1_/F(p_1_| p_2_), p_2_/F(p_2_|p_1_)} = max{p_1_F_2_(p_2_)/F(p_1_, p_2_), p_2_F_1_(p_1_)/F(p_1_, p_2_)}, with F_1_(p_1_) and F_2_(p_2_) the marginal non-null CDFs of SNPs for phenotype 1 and 2, respectively. For enriched samples, p-values will tend to be smaller than predicted from the uniform distribution, so that

F_1_(p_1_) ≥ p_1_ and F_2_(p_2_) ≥ p_2_. Then

max {p_1_F_2_(p_2_) / F(p_1_, p_2_), p_2_F_1_(p_1_) / F(p_1_, p_2_)}

≥ [π_0_ + π_1_ + π_2_] max{p_1_F_2_(p_2_) / F(p_1_, p_2_), p_2_F_1_(p_1_) / F(p_1_, p_2_)}

≥ [π_0_p_1_p_2_ + π_1_p_2_F_1_(p_1_) + π_2_p_1_F_2_(p_2_)] / F(p_1_, p_2_).

Under the assumption that SNPs are independent if one or both are null, reasonable for disjoint samples, this last quantity is precisely the conjFDR given in Eq (7). Thus, Eq (8) is a conservative model-free estimate of the conjFDR (Smeland et al., 2020).

Functional annotation and gene prioritization

Utilizing FUMA, an online annotation platform (http://fuma.ctglab.nl/), we functionally annotated all candidate SNPs in the genomic loci with a conjFDR value <0.1 and having an r2≥0.6 with one of the independent significant SNPs in the identified loci (Watanabe et al., 2017). SNPs were annotated with Combined Annotation Dependent Depletion (CADD) scores (Kircher et al., 2014), RegulomeDB scores (Boyle et al., 2012), and chromatin states (Kundaje et al., 2015; Zhu et al., 2016). The CADD score is a deleterious score of variants computed by integrating 63 functional annotations (Kircher et al., 2014). The higher the score, the more deleterious. A CADD score above 12.37 is the threshold to be potentially pathogenic (Kircher et al., 2014). The RegulomeDB score is a categorical score to guide the interpretation of regulatory variants (Boyle et al., 2012). It is based on information from expression quantitative trait locus (eQTL) and chromatin marks, ranging from 1a to 7 with lower scores indicating an increased likelihood of having a regulatory function. Scores are as follows: 1a=eQTL + Transcription Factor (TF) binding + matched TF motif + matched DNase Footprint + DNase peak; 1b=eQTL + TF binding + any motif + DNase Footprint + DNase peak; 1c=eQTL + TF binding + matched TF motif + DNase peak; 1d=eQTL + TF binding + any motif + DNase peak; 1e=eQTL + TF binding + matched TF motif; 1f=eQTL + TF binding / DNase peak; 2a=TF binding + matched TF motif + matched DNase Footprint + DNase peak; 2b=TF binding + any motif + DNase Footprint + DNase peak; 2c=TF binding + matched TF motif + DNase peak; 3a=TF binding + any motif + DNase peak; 3b=TF binding + matched TF motif; 4=TF binding + DNase peak; 5=TF binding or DNase peak; 6=other; 7=Not available (Boyle et al., 2012). The chromatin state represents the accessibility of genomic regions (every 200bp) with 15 categorical states predicted by a hidden Markov model based on 5 chromatin marks for 127 epigenomes in the Roadmap Epigenomics Project (Roadmap Epigenomics Consortium 2015). A lower state indicates higher accessibility, with states 1-7 referring to open chromatin states. We annotated the minimum chromatin state across tissues to SNPs. The 15-core chromatin states as suggested by Roadmap are as follows: 1=Active Transcription Start Site (TSS); 2=Flanking Active TSS; 3=Transcription at gene 5’ and 3’; 4=Strong transcription; 5= Weak Transcription; 6=Genic enhancers; 7=Enhancers; 8=Zinc finger genes & repeats; 9=Heterochromatic; 10=Bivalent/PoisedTSS; 11=Flanking Bivalent/Poised TSS/Enh; 12=Bivalent Enhancer; 13=Repressed PolyComb; 14=Weak Repressed PolyComb; 15=Quiescent/Low. Standardized SNP effect sizes were calculated for the most impactful SNPs by transforming the sample size-weighted meta-analysis Z score, as described by Zhu et al (Zhu et al., 2016).

Furthermore, we applied V2G, a tool developed by Open Targets Genetics to prioritize genes according to the highest overall V2G score (Ghoussaini et al., 2021). For each genetic variant, the overall V2G score aggregates differentially weighted evidence of variant–gene associations from several data sources, including molecular cis-QTL data (for example, cis-protein QTLs, cis-eQTLs from GTEx v.8 and so on), interaction-based datasets (for example, promoter capture Hi-C), genomic distance and variant effect predictions (VEP) from Ensembl (Sun et al., 2018). See (https://genetics-docs.opentargets.org/our-approach/data-pipeline) for a detailed description of the evidence sources and weights used (Ghoussaini et al., 2021). We applied V2G to each lead SNP in the identified loci

Next, we used publicly available data from the Genotype-Tissue Expression (GTEx) dataset v8 as implemented in FUMA to determine the expression of the genes mapped to the shared loci with Open Targets across 54 human tissue types (GTEx Consortium 2017; Watanabe et al., 2017). The GTEx v8 dataset is based on multiple tissues from 948 deceased donors, where expression data was determined using Illumina TrueSeq RNA sequencing. For full details, see http://www.gtexportal.org or the original publication (GTEx Consortium 2017).

**Supplementary Figures:**

**d)**

**c)**

**b)**

**a)**


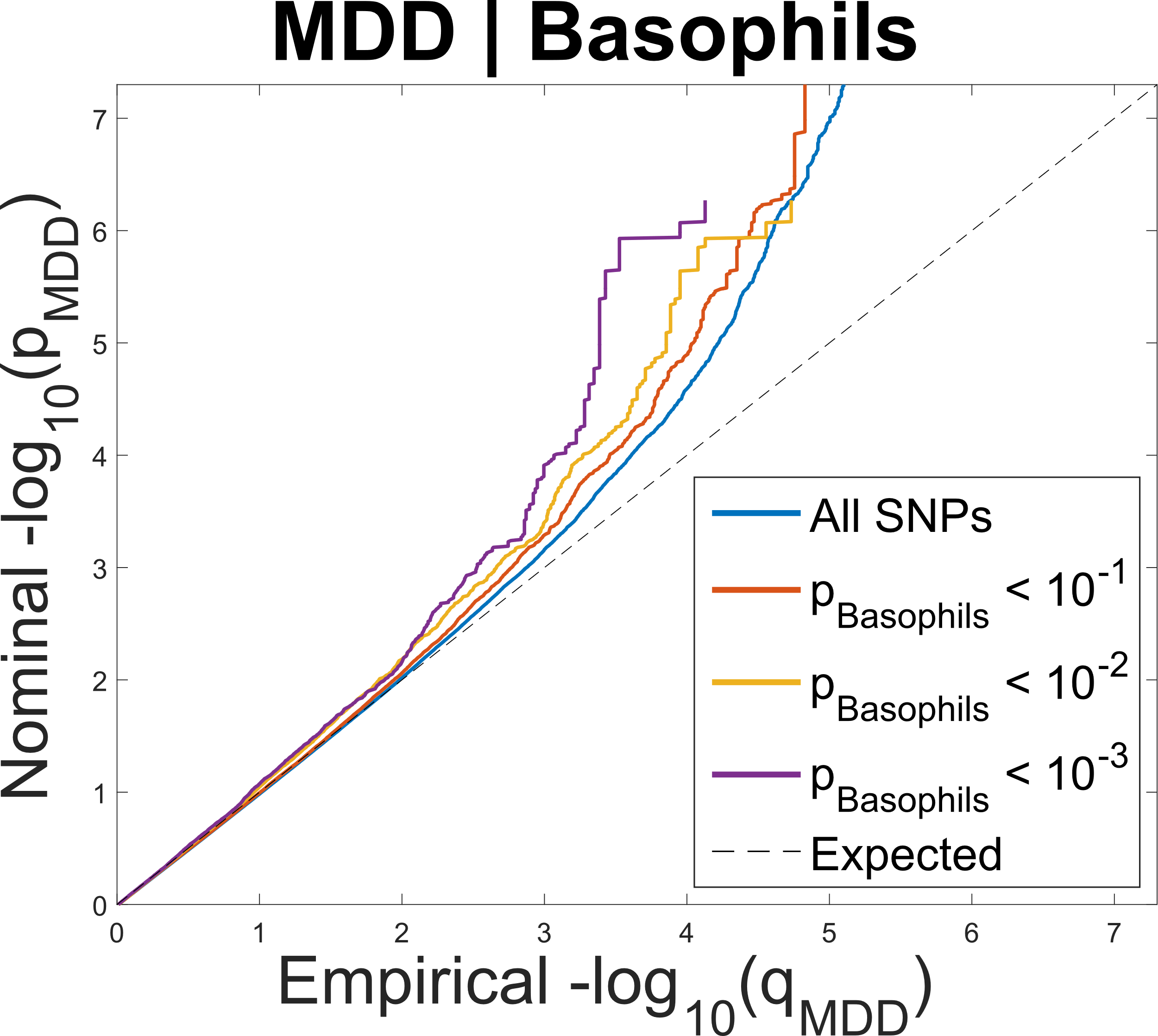

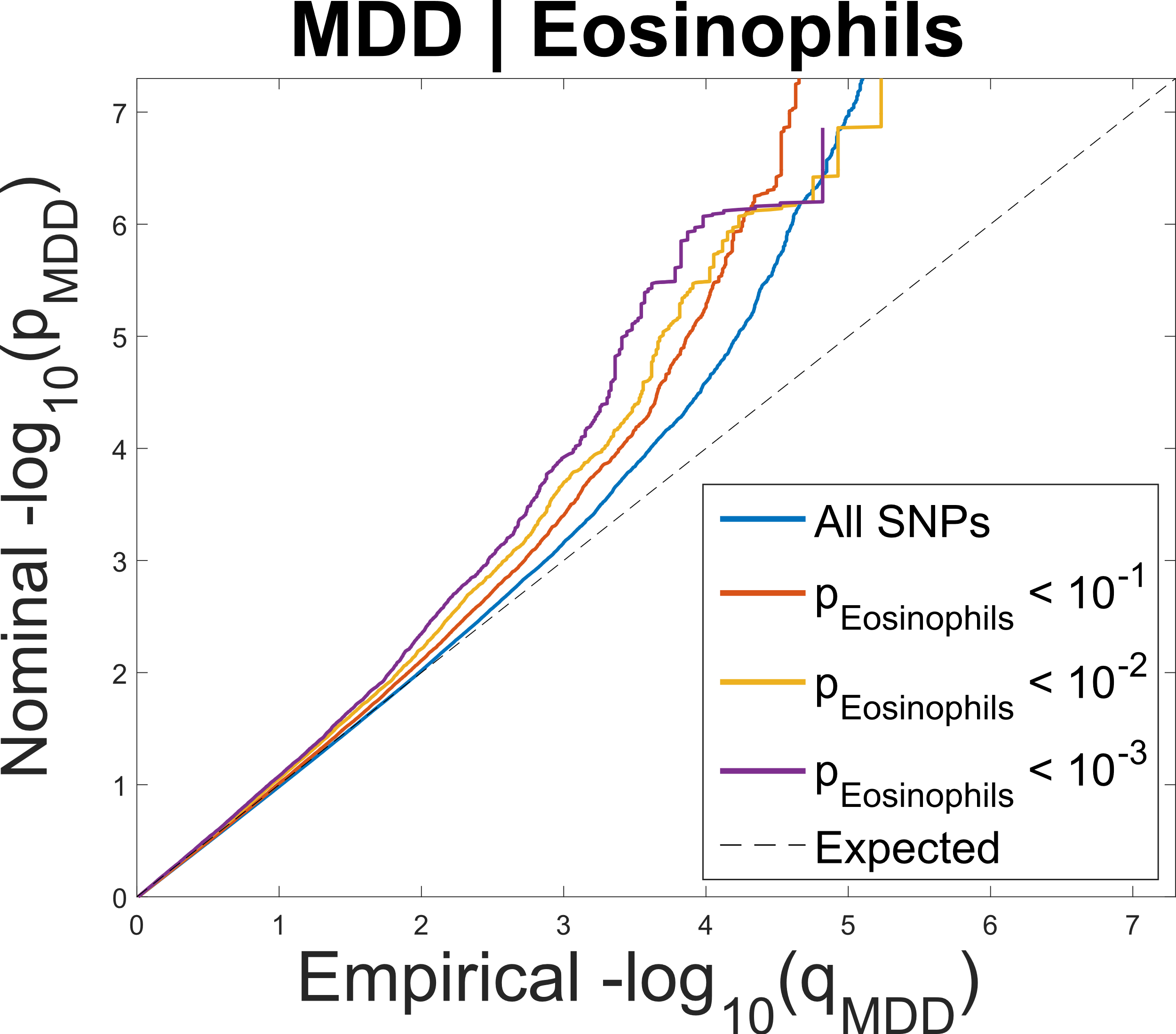

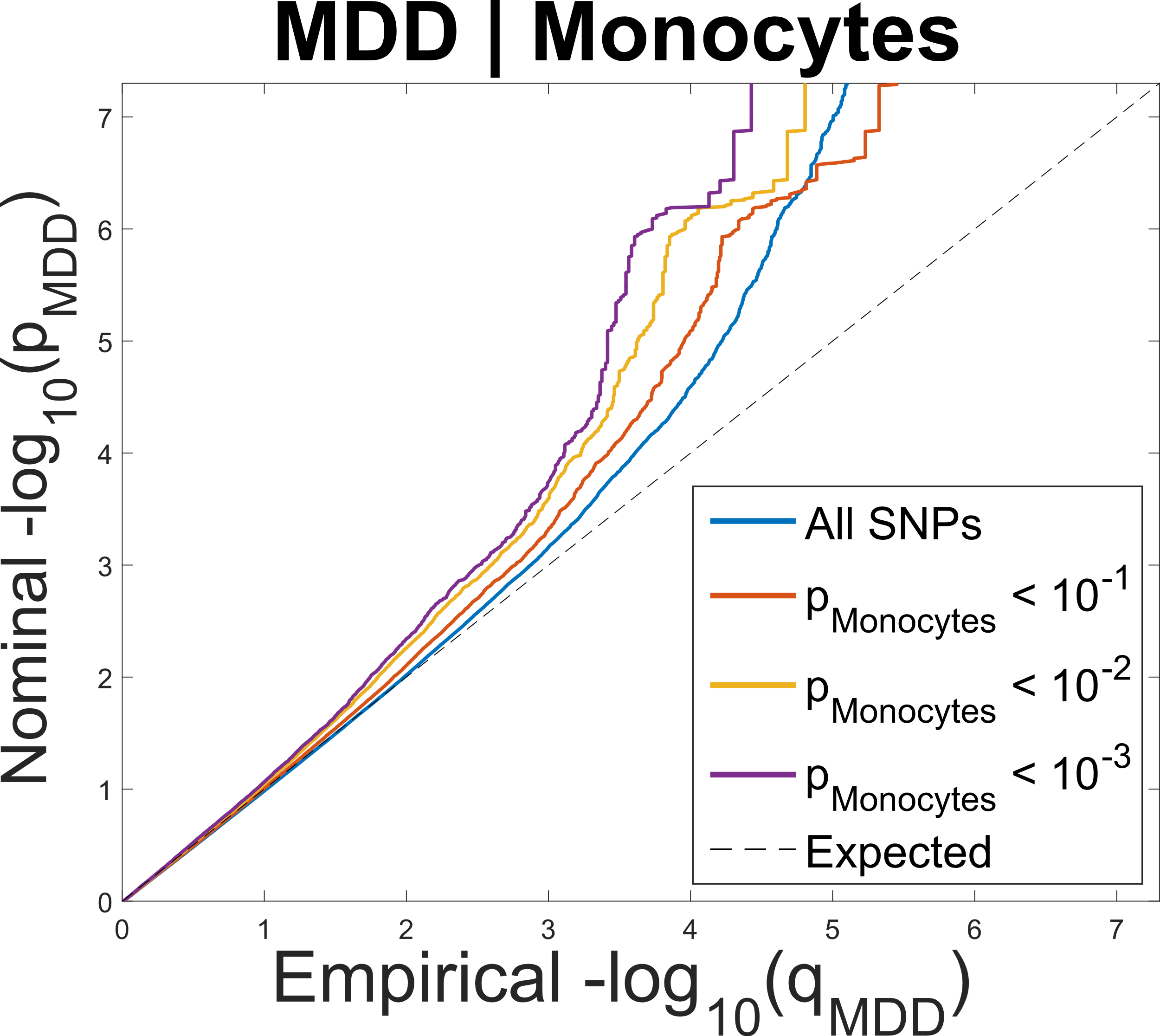

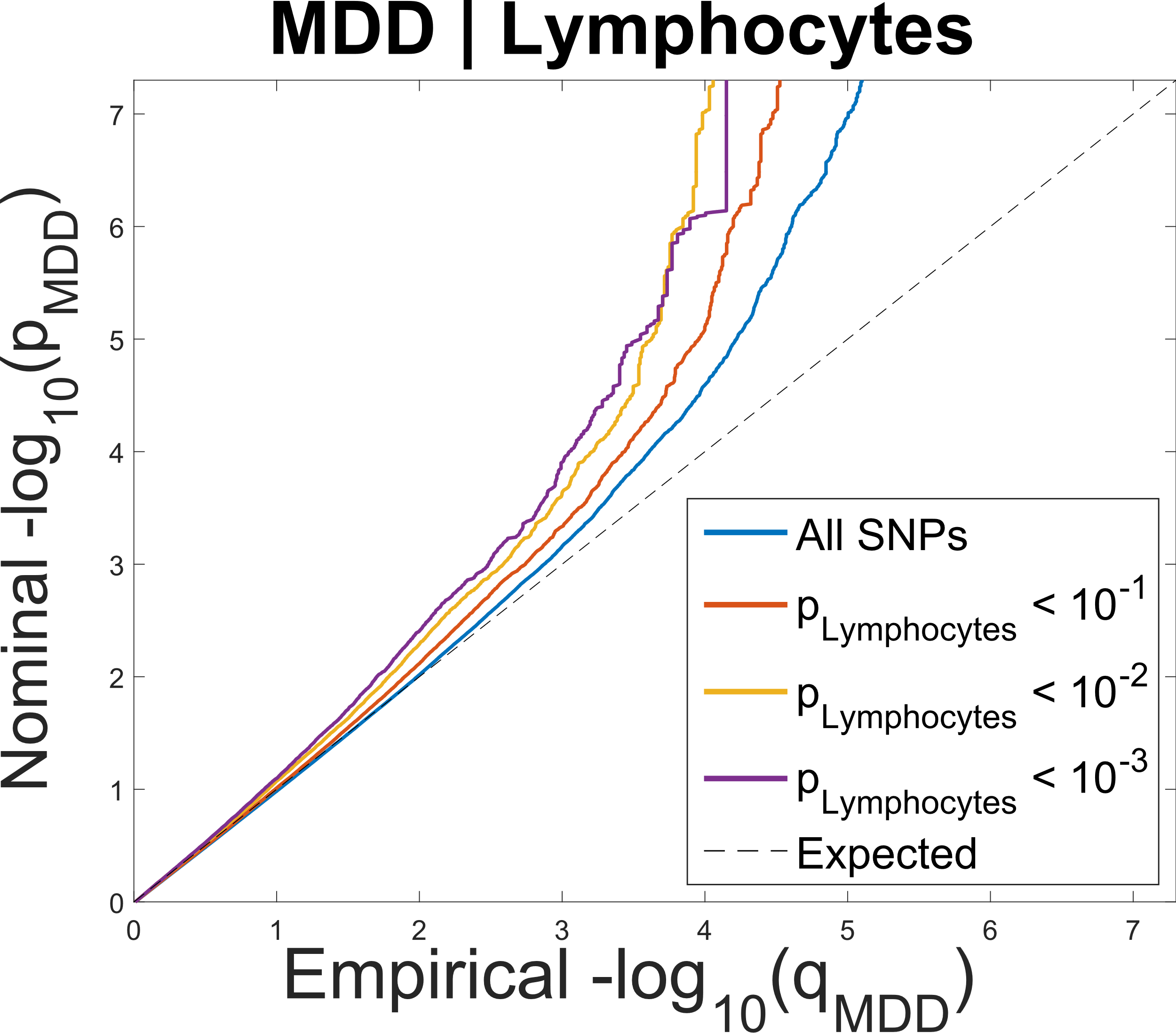


**e)**

**f)**


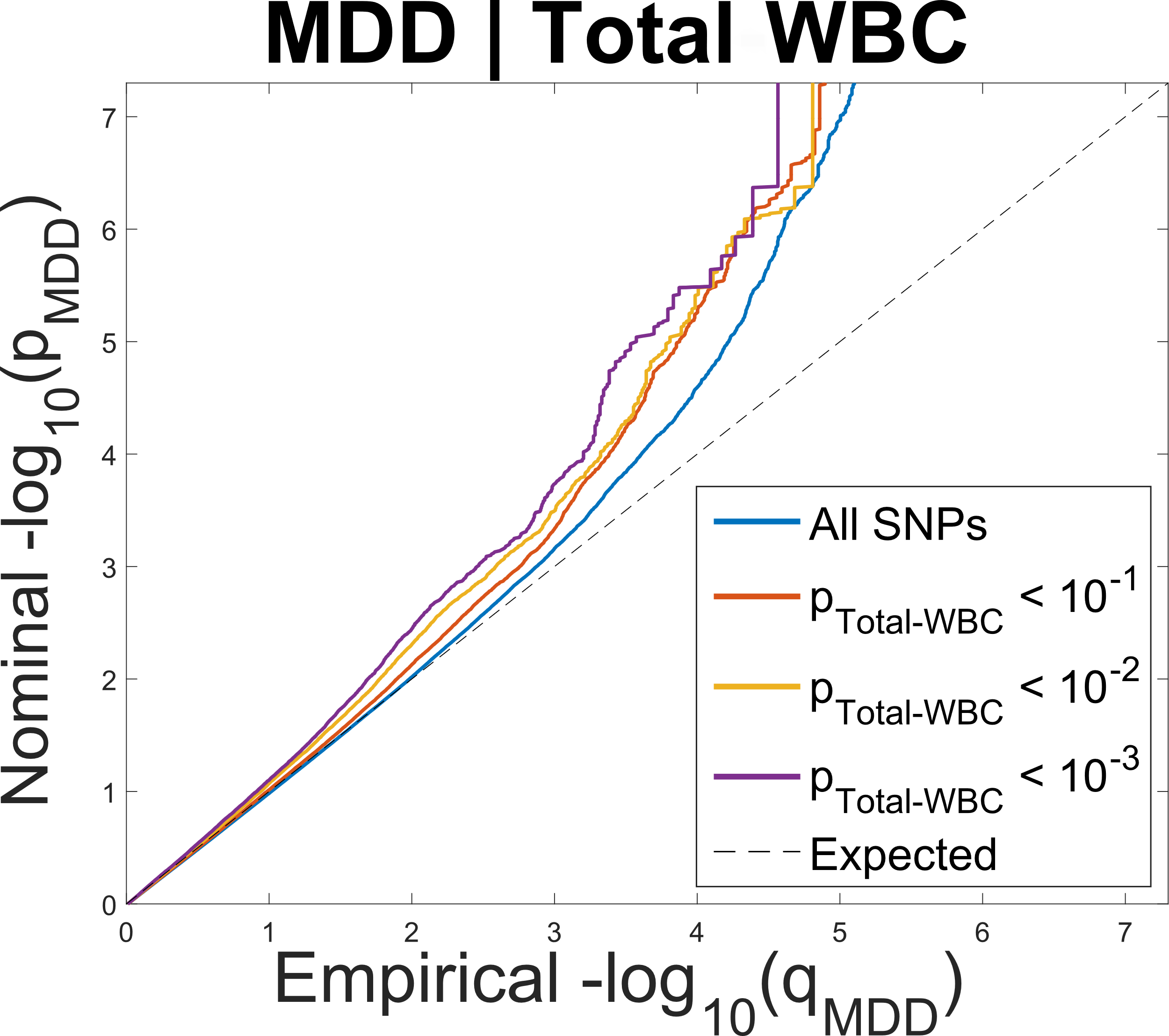

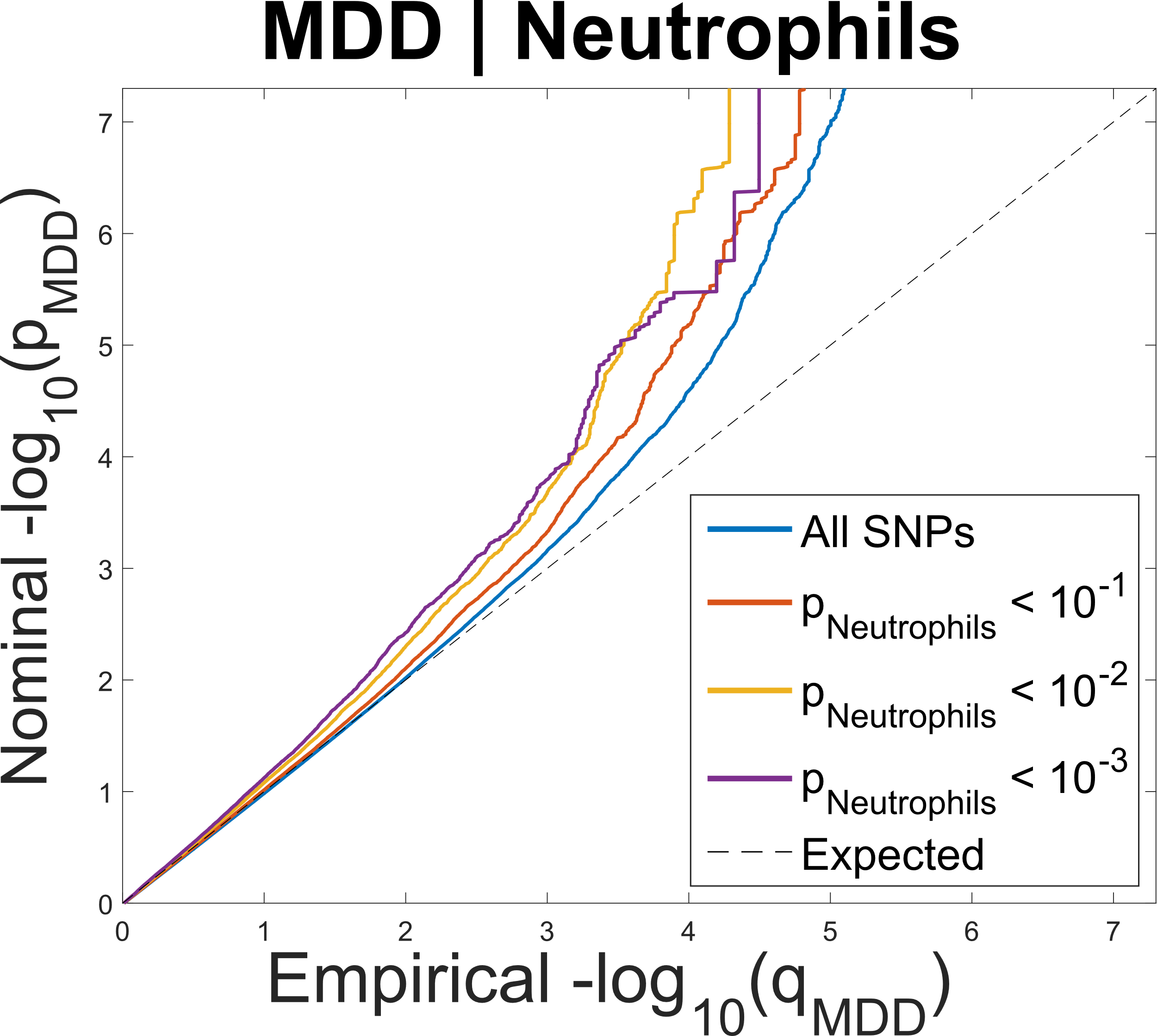


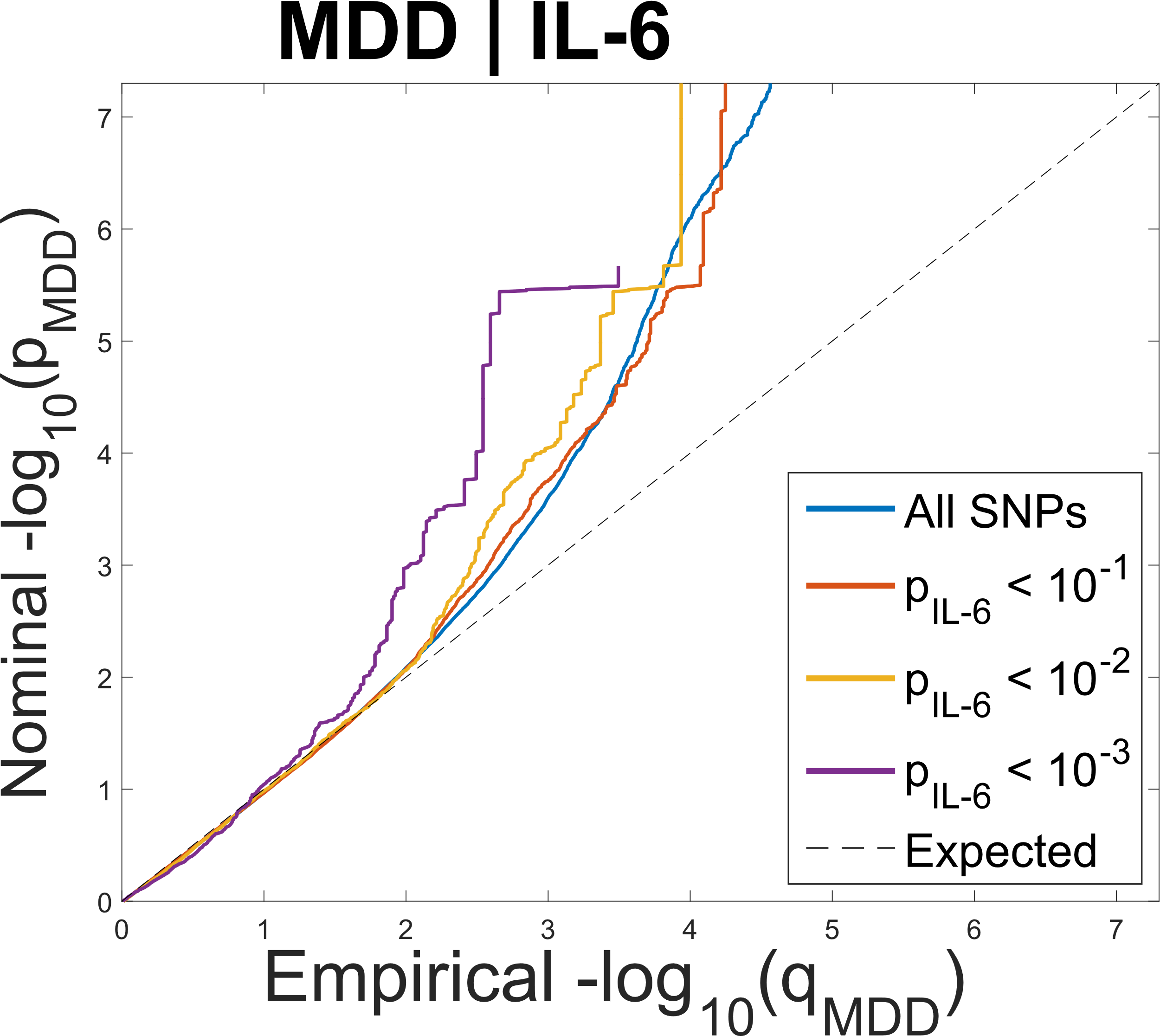


**g)**

**Supplementary Figure 1a-g.** Conditional quantile-quantile (Q-Q) plots demonstrate the enrichment of the primary phenotype major depressive disorder (MDD) as a function of increasing association with a) basophils, b) eosinophils, c) monocytes, d) lymphocytes, e) neutrophils, f) total white blood cells (WBC) and g) interleukin 6 (IL-6). The distribution of *p*-values for MDD for all SNPs is represented by the blue line, while the distribution of *p*-values for a subset of SNPs conditioned on their association with the secondary phenotype, at the levels of *p*≤ 0.1, *p*≤0.01 and *p*≤0.001 respectively, are represented with the red, yellow and purple lines. The y-axis represents the nominal (observed) -log_10_ *p*-values, the *x*-axis represents the empirical (expected) -log_10_ *p*-values. The black dotted diagonal line serves as indicator for the null hypothesis.


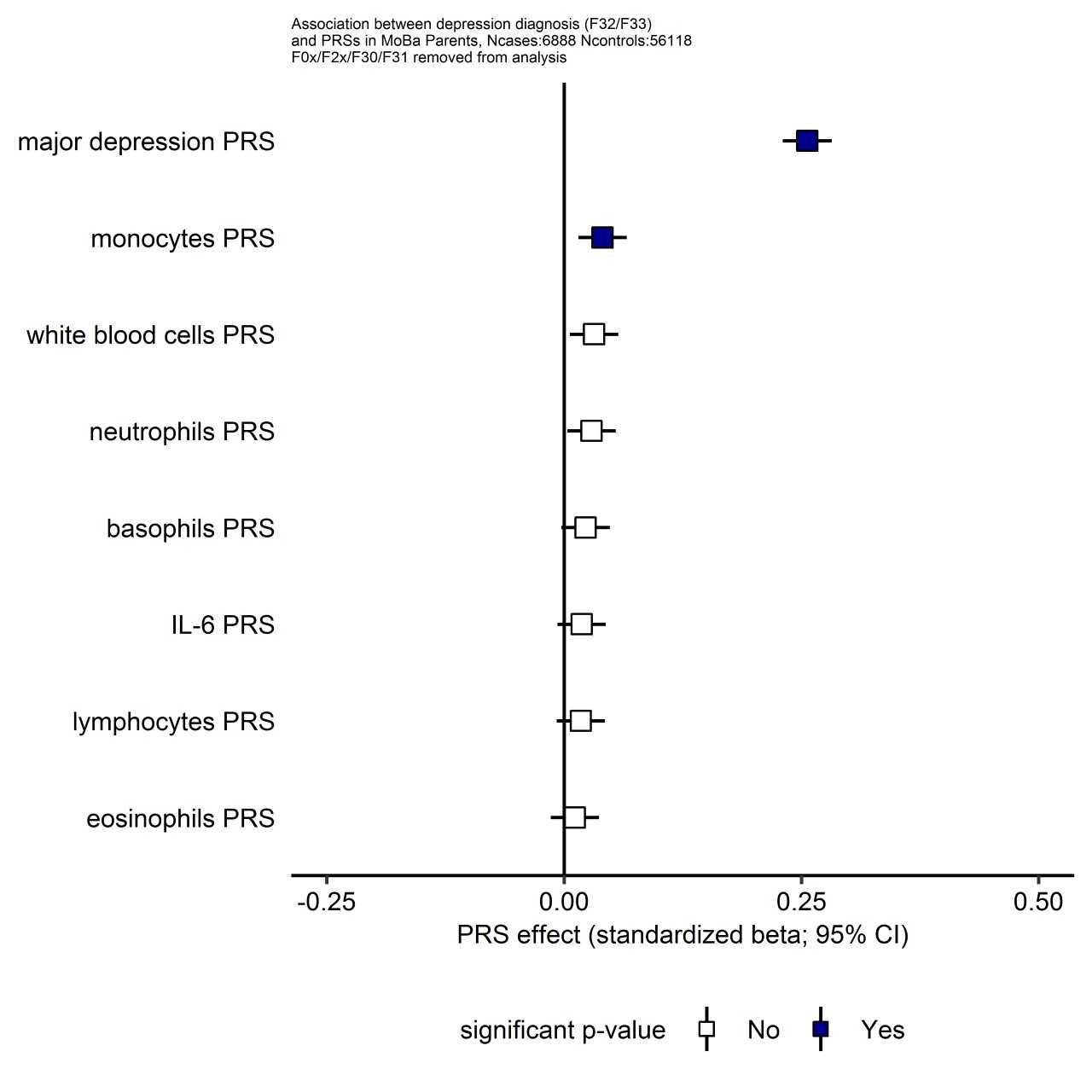


**Supplementary Figure 2.** The association between polygenic risk scores (PRS) for major depression and immunological traits with case-control status of major depressive disorder (MDD) in the MoBa sample. The results are corrected for smoking and BMI. The risk alleles are weighted based on effect sizes uncovered in recent GWAS. The *x*-axis shows absolute PRS values. The filled squares denoting significant findings following FDR correction for multiple testing.


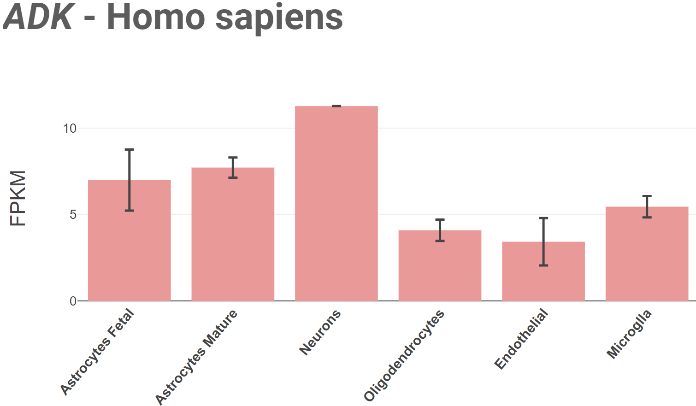

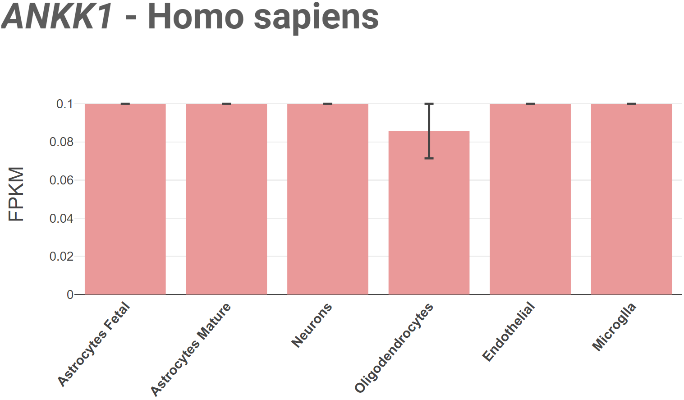


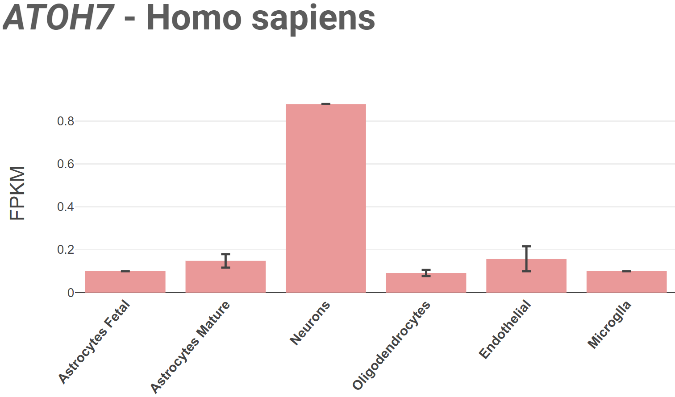

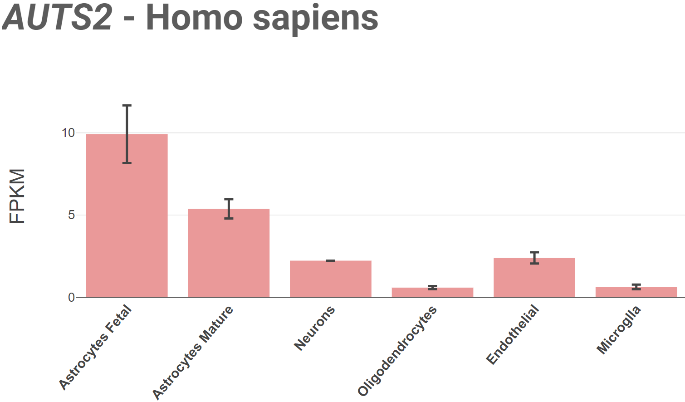


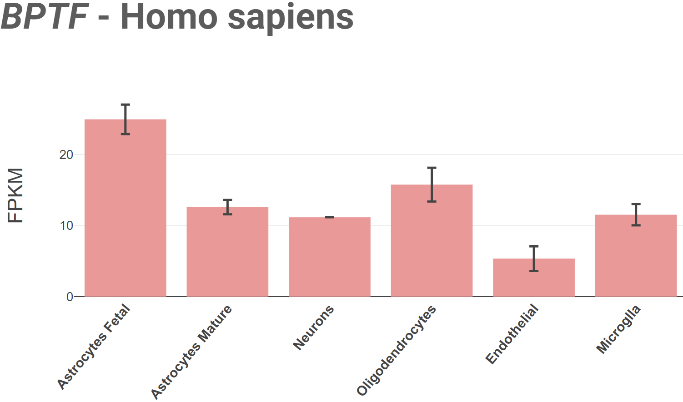

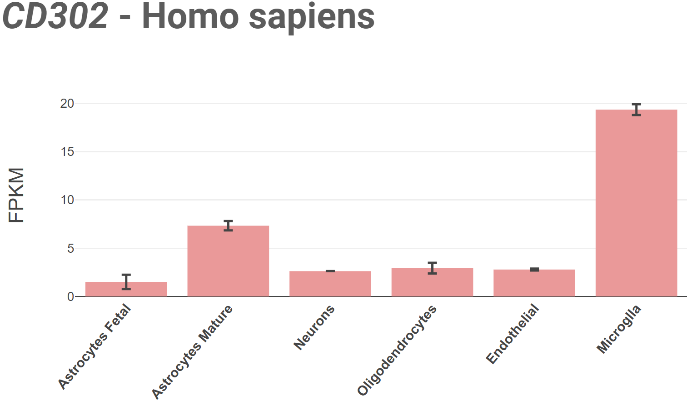


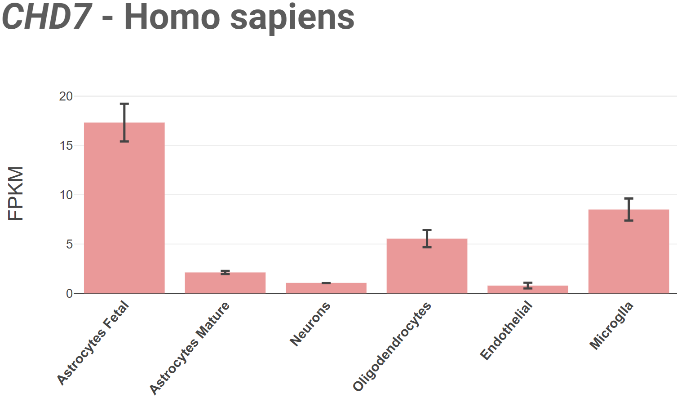

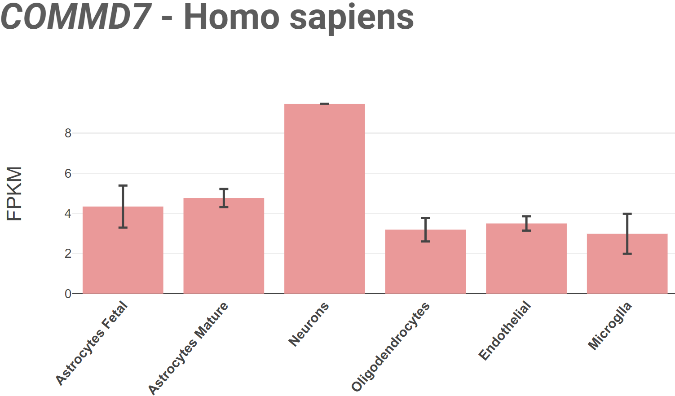


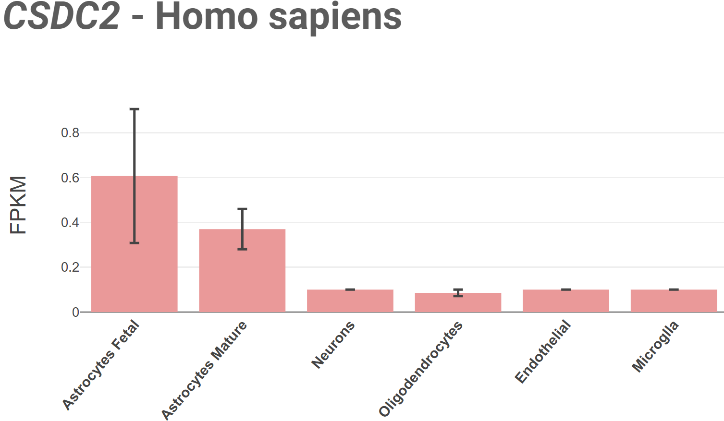

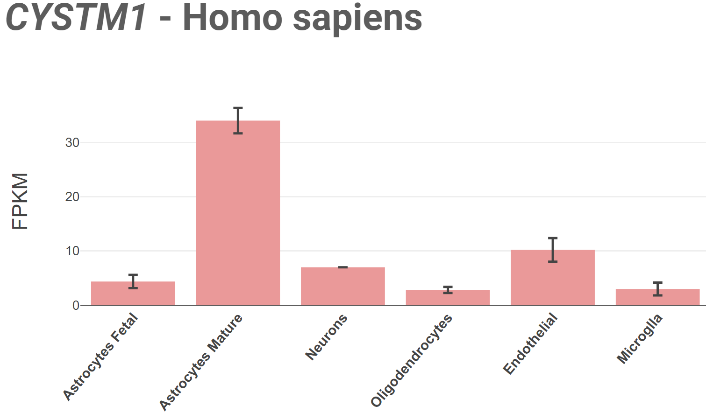


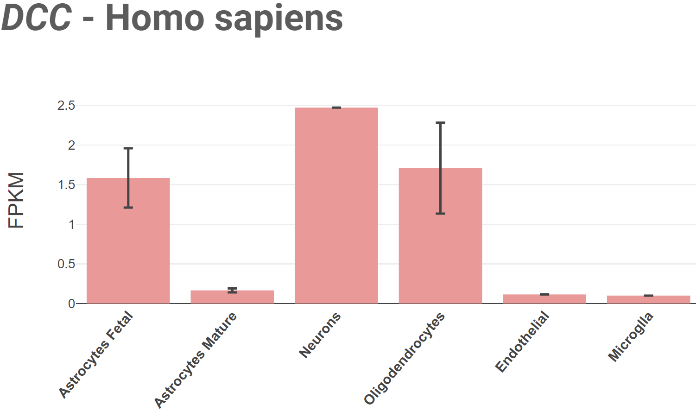

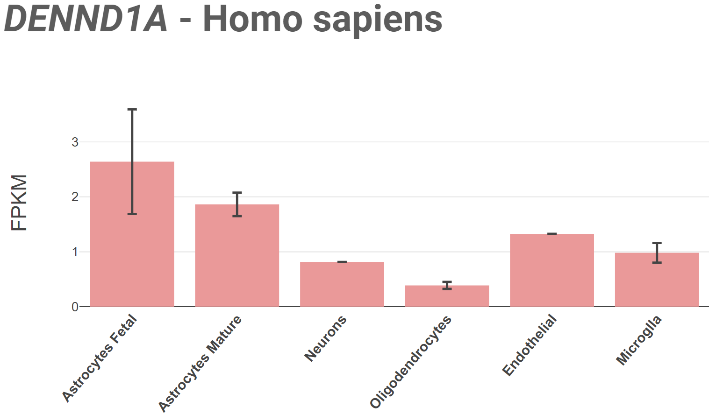


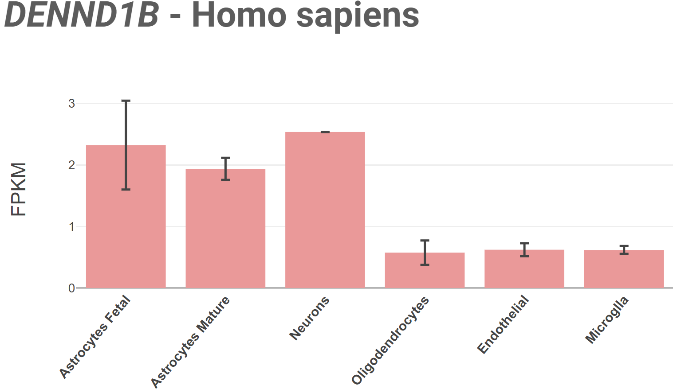

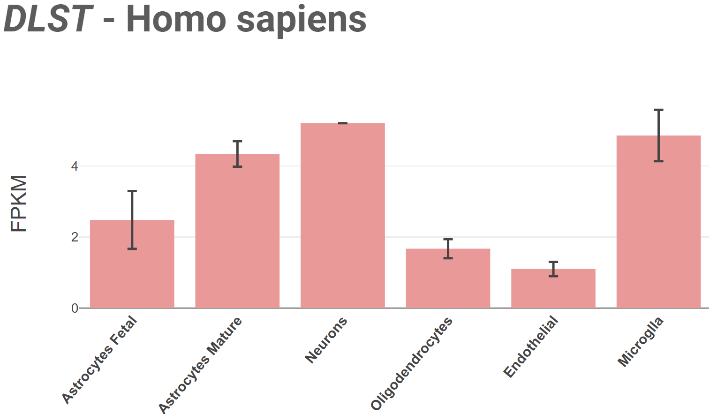


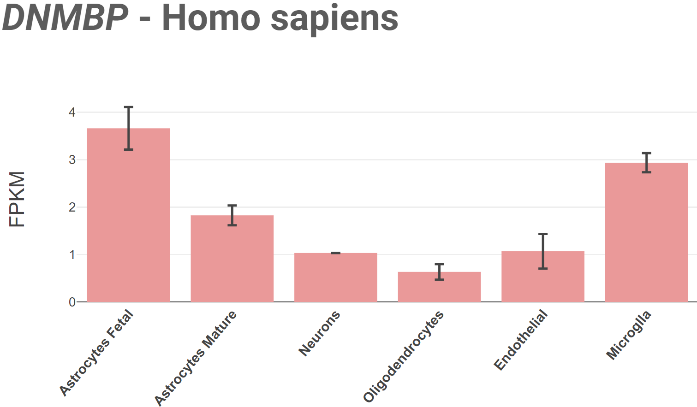

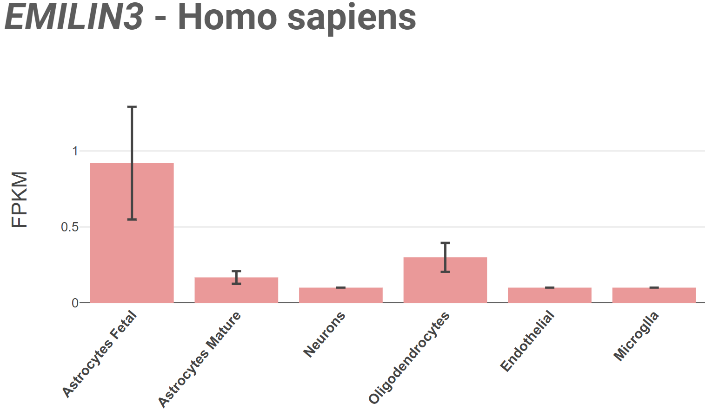


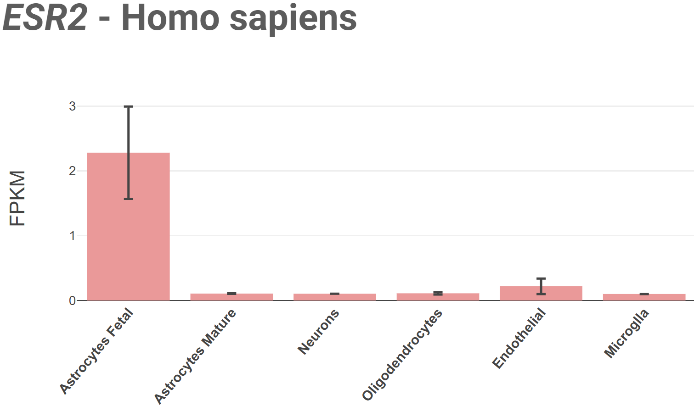

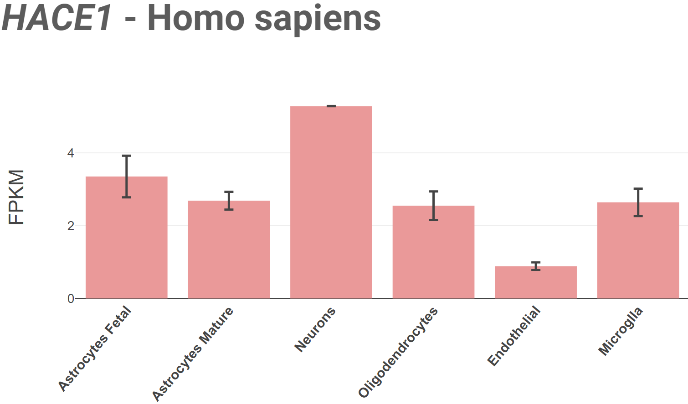


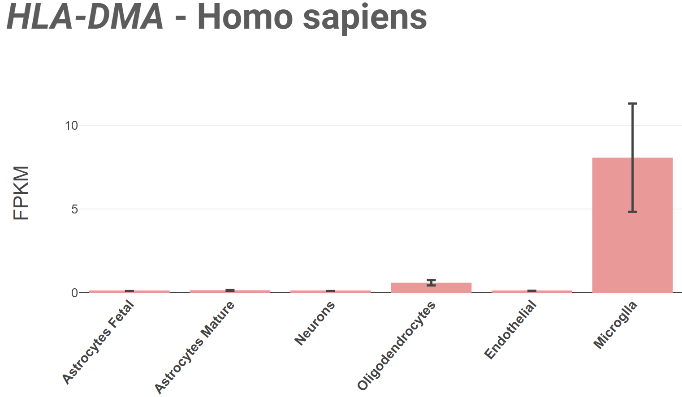

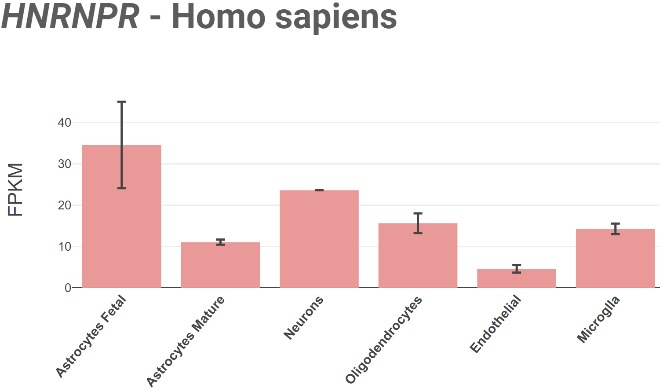


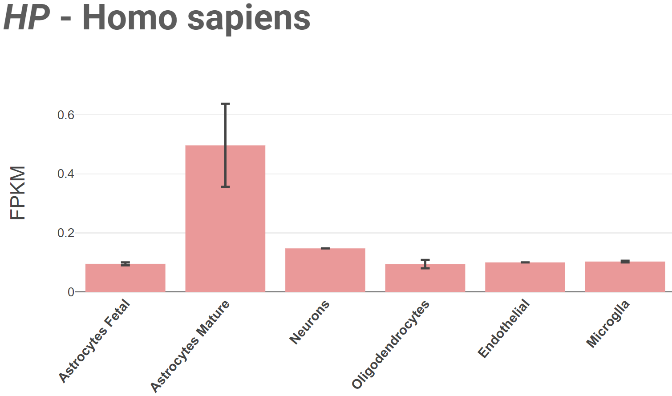

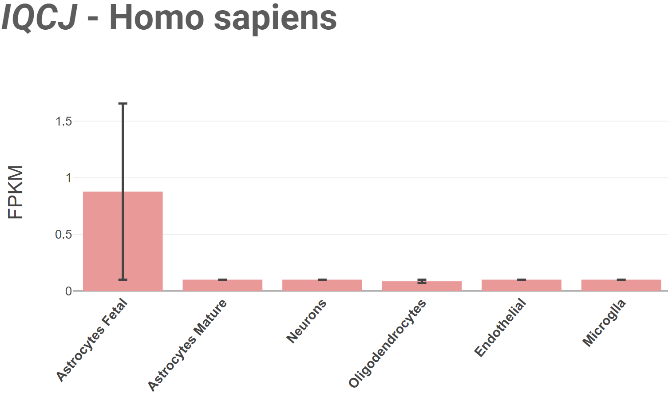


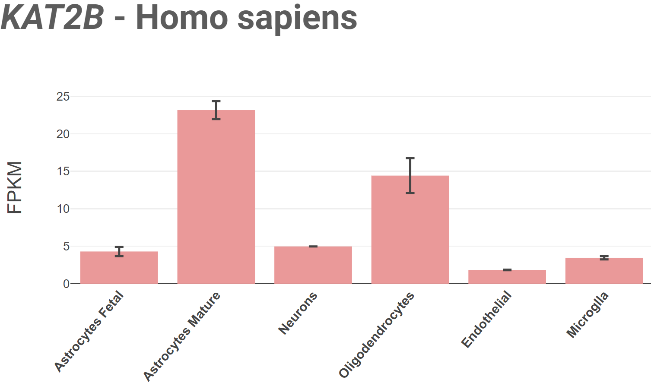

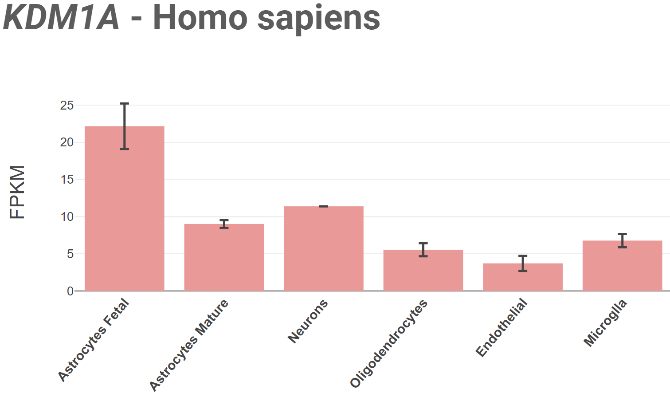


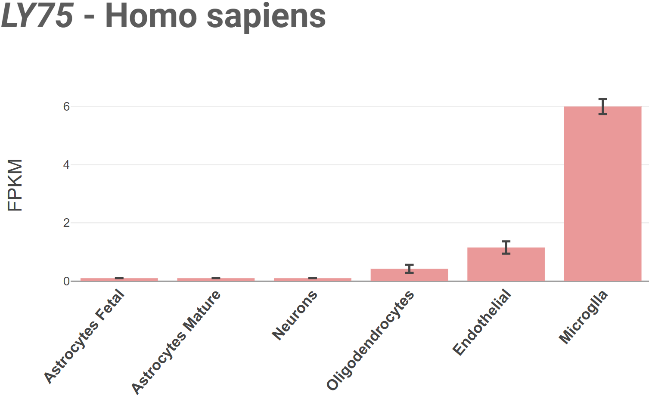

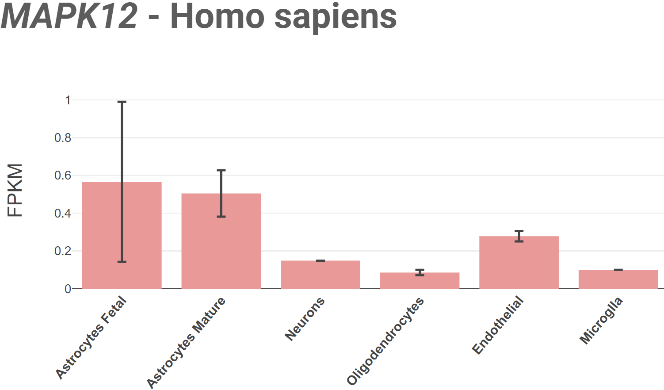


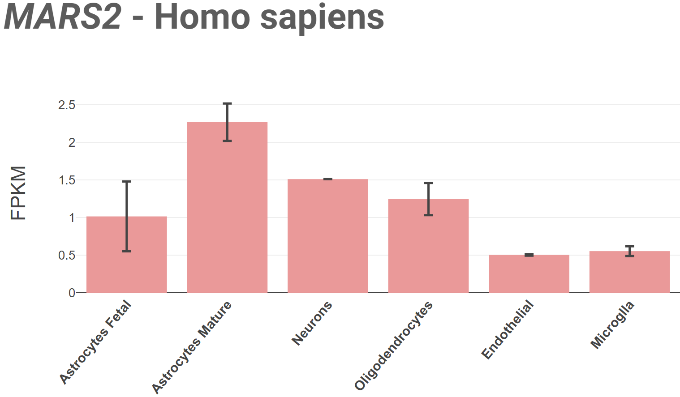

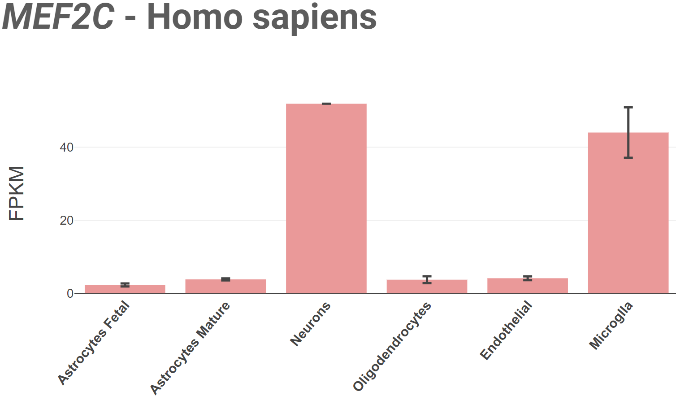


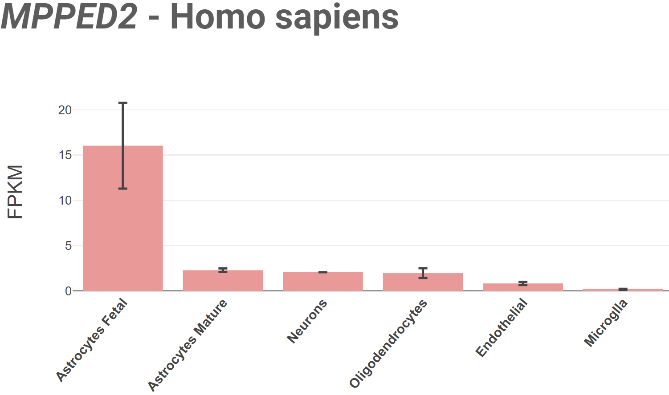

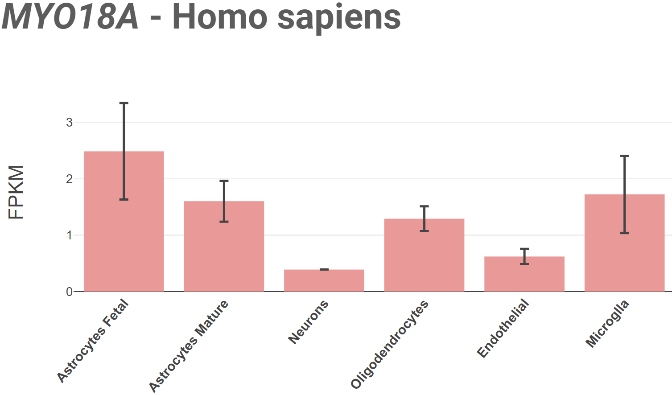


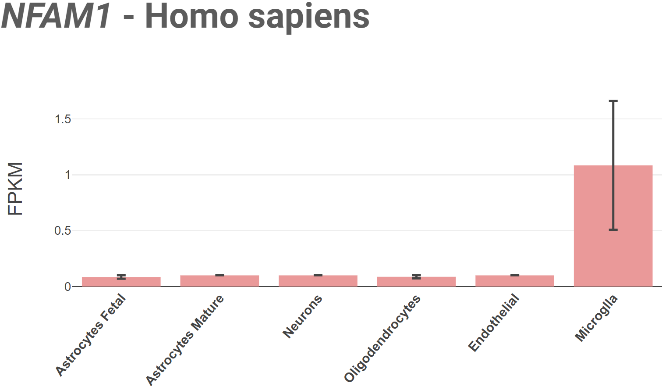

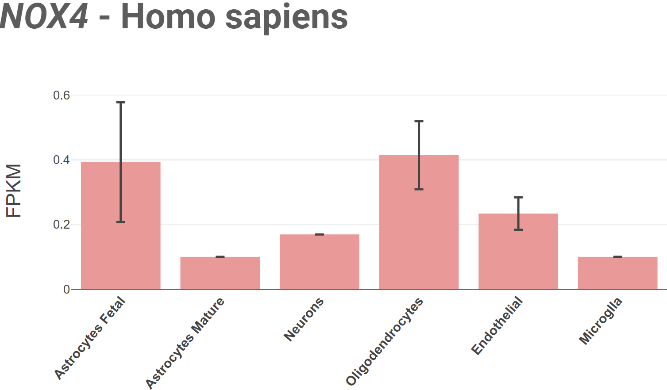


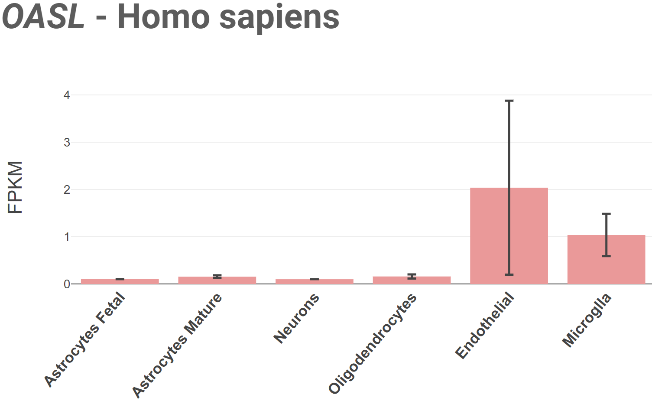

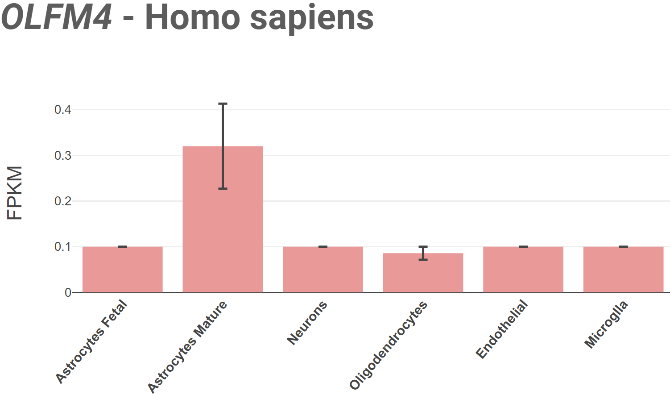


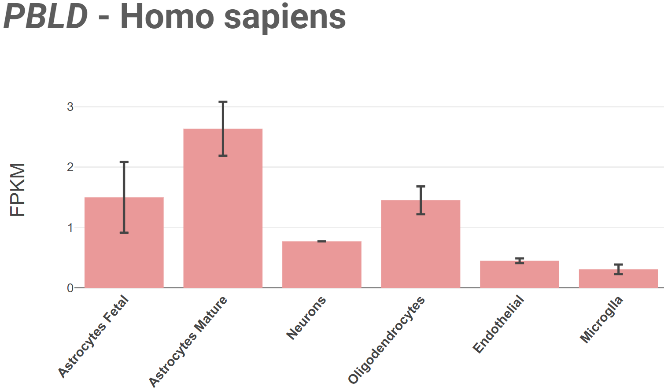

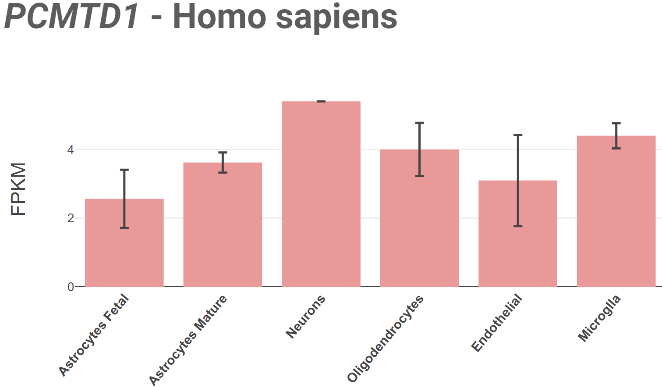


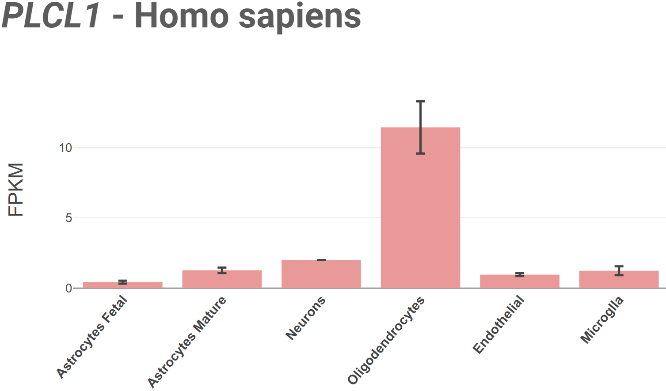

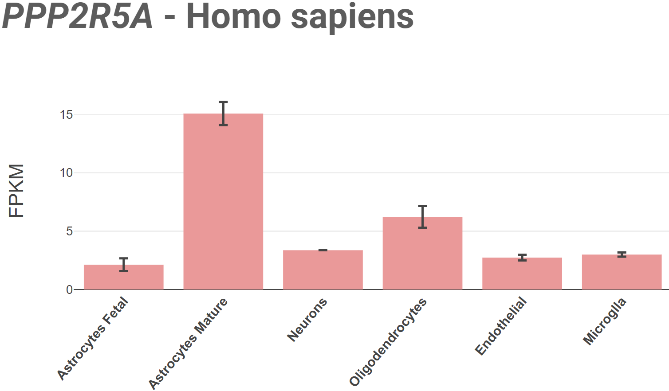


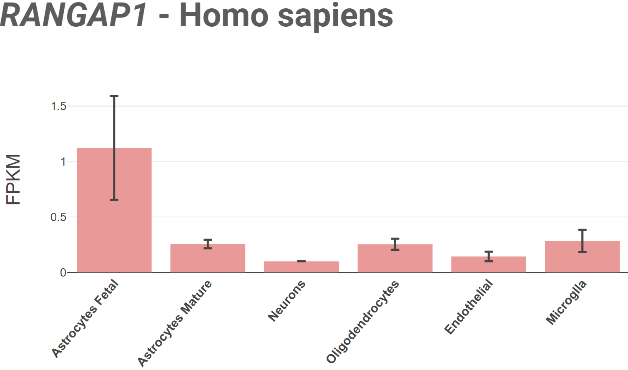

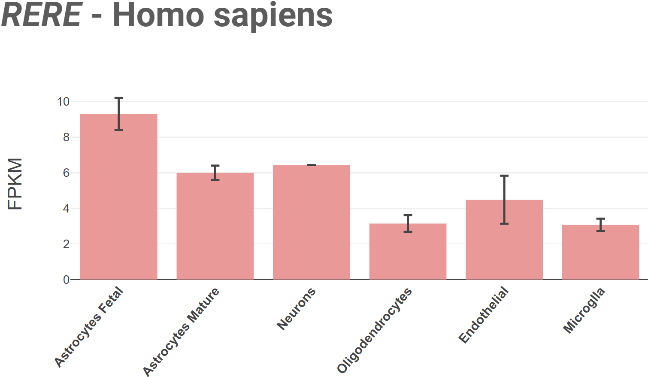


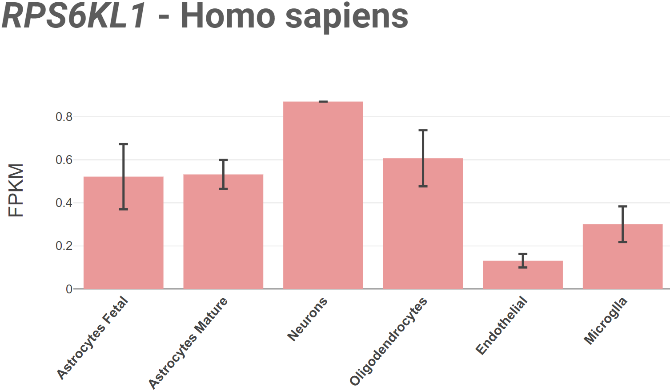

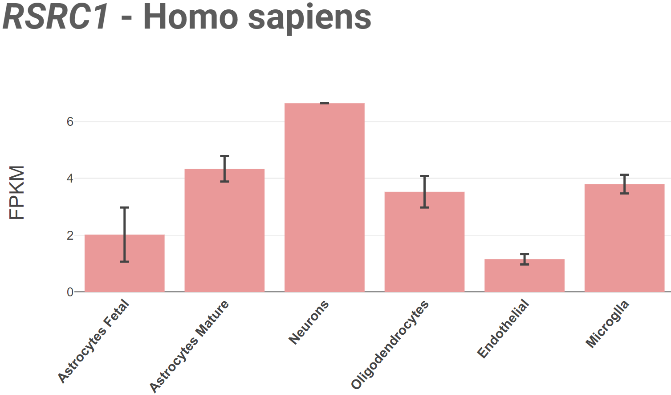


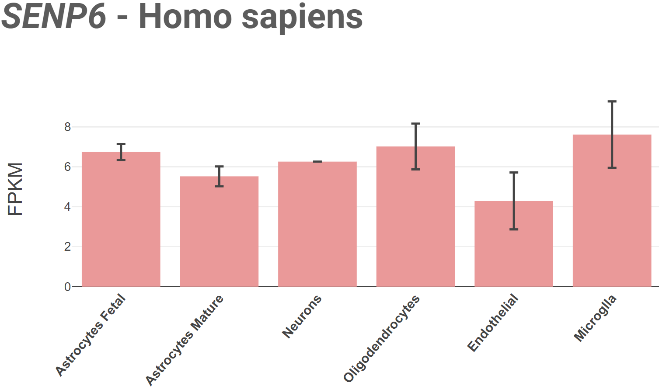

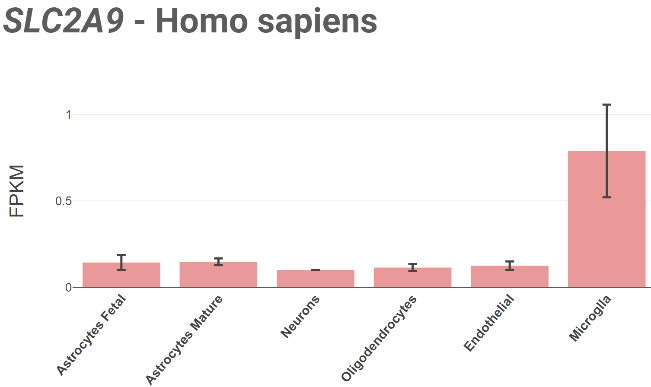


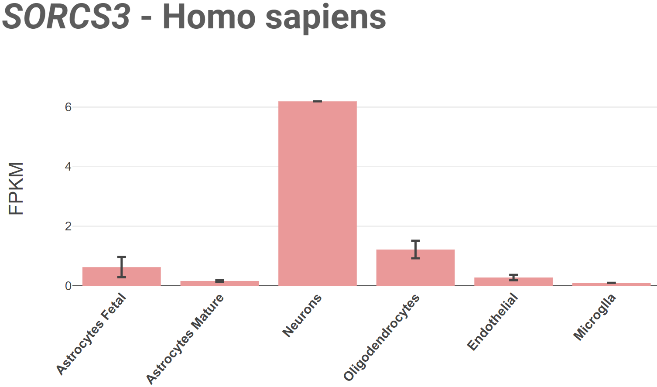

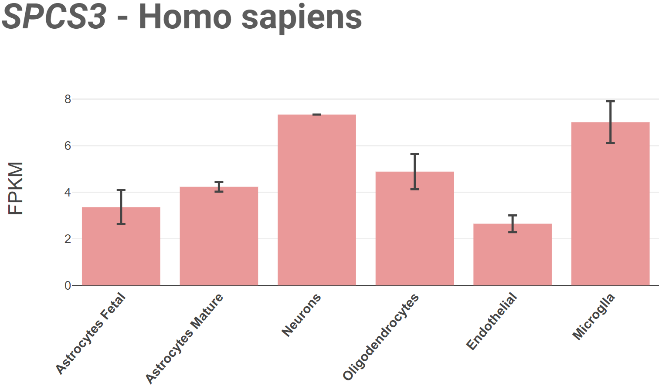


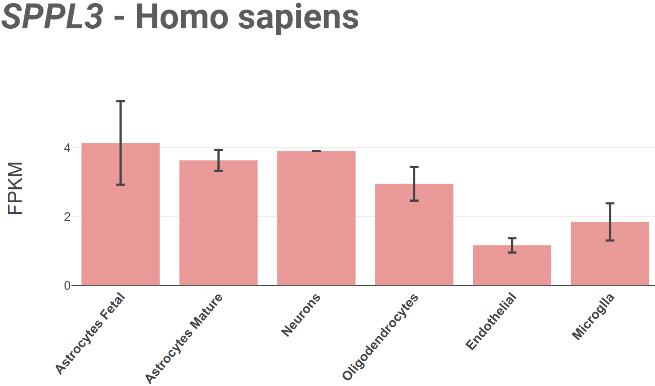

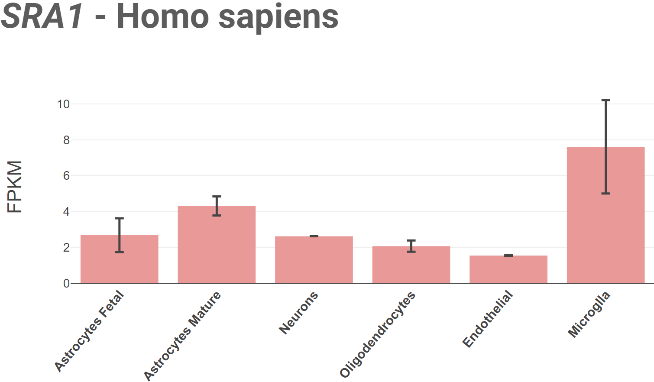


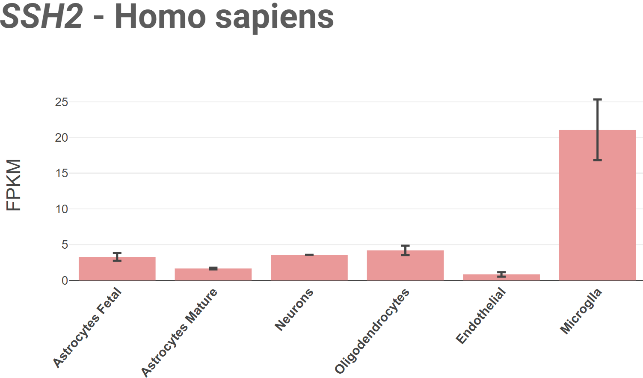

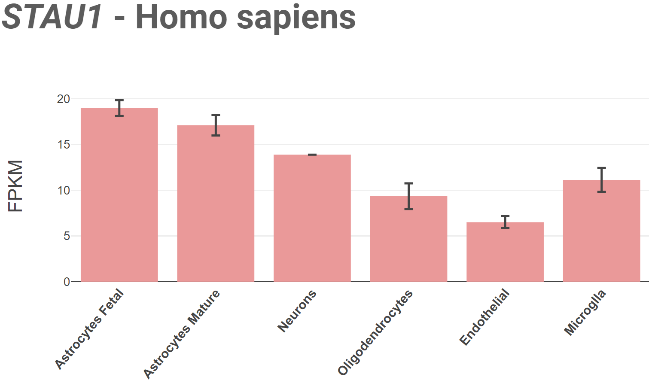


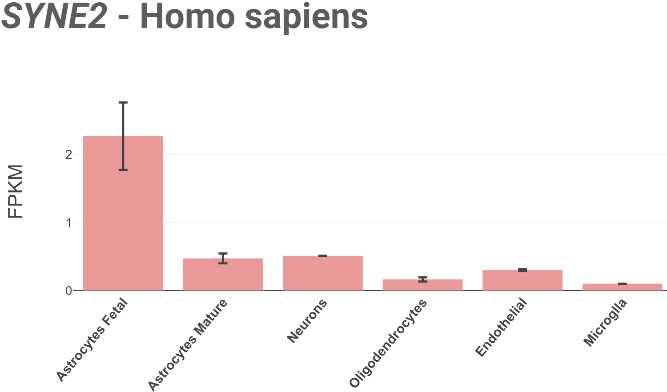

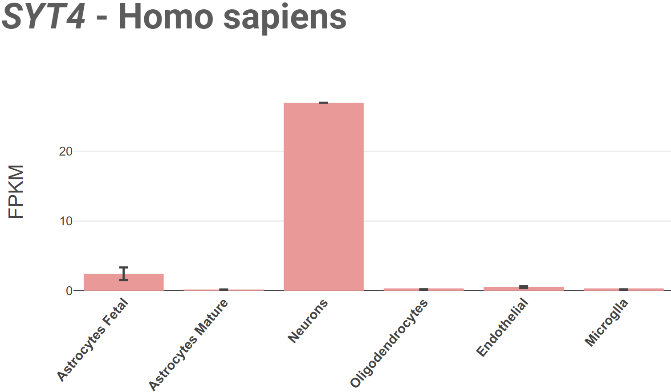


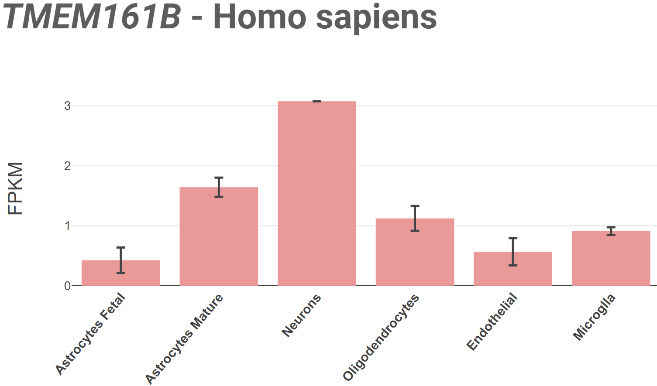

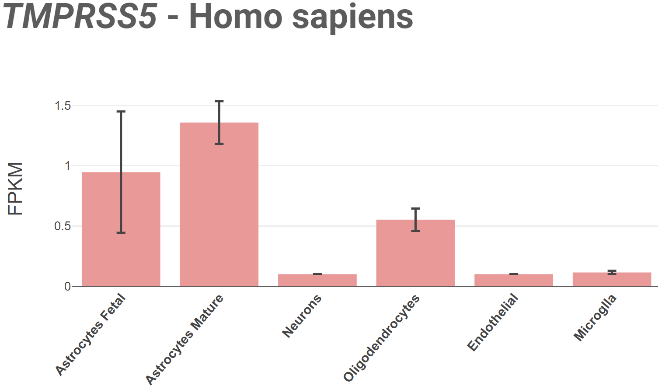


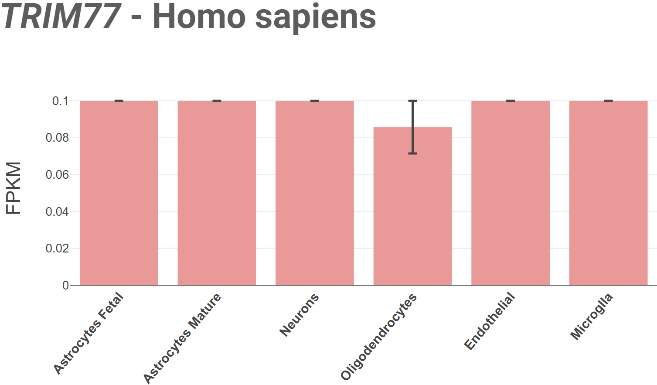

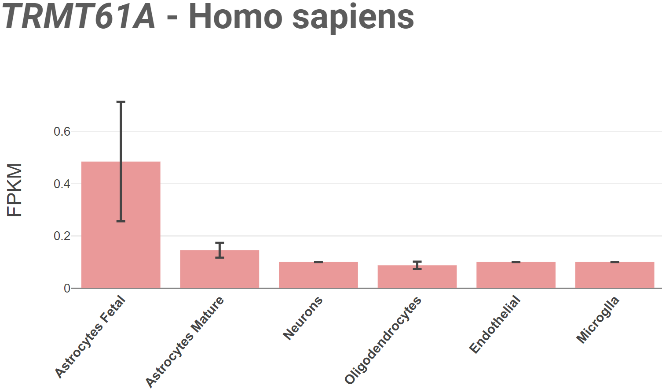


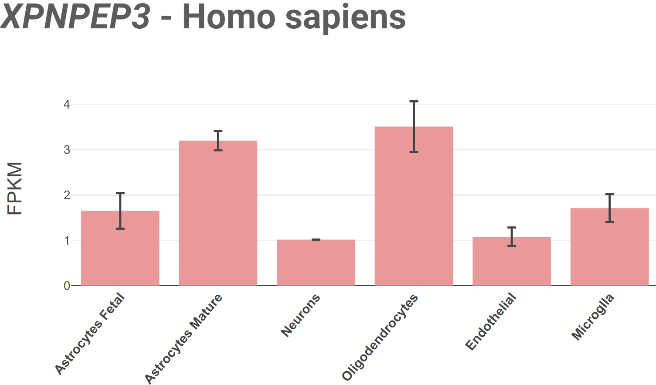

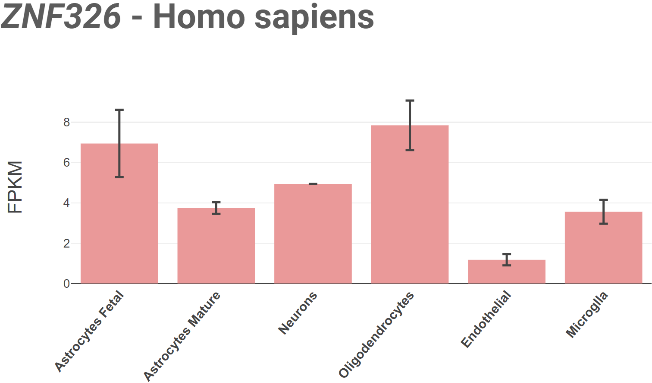


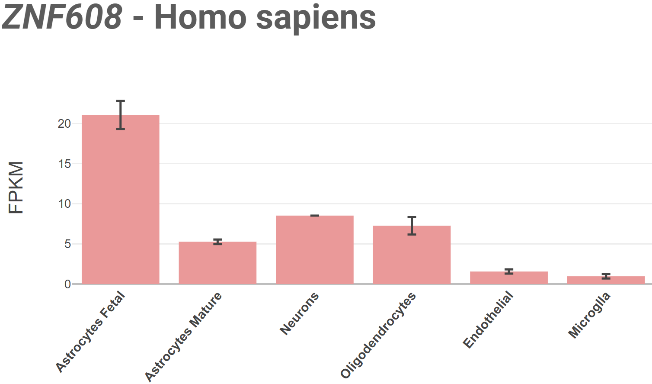


**Supplementary Figure 3.**

Shows the expression of genes implicated by shared loci in six different cell types in the cerebral cortex, using a publicly available RNA-sequencing and splicing database. The genes are the top genes identified by Open Targets to the loci shared between MDD and WBCs or IL-6 at conjFDR <0.05.

**Supplementary References**

Ahluwalia, T.S., Prins, B.P., Abdollahi, M., Armstrong, N.J., Aslibekyan, S., Bain, L., Jefferis, B., Baumert, J., Beekman, M., Ben-Shlomo, Y., 2021. Genome-wide association study of circulating interleukin 6 levels identifies novel loci. Human molecular genetics 30, 393-409.

Andreassen, O.A., Desikan, R.S., Wang, Y., Thompson, W.K., Schork, A.J., Zuber, V., Doncheva, N.T., Ellinghaus, E., Albrecht, M., Mattingsdal, M., 2015a. Abundant genetic overlap between blood lipids and immune-mediated diseases indicates shared molecular genetic mechanisms. PloS one 10, e0123057.

Andreassen, O.A., Djurovic, S., Thompson, W.K., Schork, A.J., Kendler, K.S., O’Donovan, M.C., Rujescu, D., Werge, T., van de Bunt, M., Morris, A.P., 2013a. Improved detection of common variants associated with schizophrenia by leveraging pleiotropy with cardiovascular-disease risk factors. The American Journal of Human Genetics 92, 197-209.

Andreassen, O.A., Harbo, H.F., Wang, Y., Thompson, W.K., Schork, A.J., Mattingsdal, M., Zuber, V., Bettella, F., Ripke, S., Kelsoe, J., 2015b. Genetic pleiotropy between multiple sclerosis and schizophrenia but not bipolar disorder: differential involvement of immune-related gene loci. Molecular psychiatry 20, 207-214.

Andreassen, O.A., Thompson, W.K., Dale, A.M., 2014. Boosting the power of schizophrenia genetics by leveraging new statistical tools. Schizophrenia bulletin 40, 13-17.

Andreassen, O.A., Thompson, W.K., Schork, A.J., Ripke, S., Mattingsdal, M., Kelsoe, J.R., Kendler, K.S., O'Donovan, M.C., Rujescu, D., Werge, T., 2013b. Improved detection of common variants associated with schizophrenia and bipolar disorder using pleiotropy-informed conditional false discovery rate. PLoS genetics 9, e1003455.

Boyle, A.P., Hong, E.L., Hariharan, M., Cheng, Y., Schaub, M.A., Kasowski, M., Karczewski, K.J., Park, J., Hitz, B.C., Weng, S., 2012. Annotation of functional variation in personal genomes using RegulomeDB. Genome research 22, 1790-1797.

Bulik-Sullivan, B.K., Loh, P.-R., Finucane, H.K., Ripke, S., Yang, J., Consortium, S.W.G.o.t.P.G., Patterson, N., Daly, M.J., Price, A.L., Neale, B.M., 2015. LD Score regression distinguishes confounding from polygenicity in genome-wide association studies. Nature genetics 47, 291-295.

Chen, M.-H., Raffield, L.M., Mousas, A., Sakaue, S., Huffman, J.E., Moscati, A., Trivedi, B., Jiang, T., Akbari, P., Vuckovic, D., 2020. Trans-ethnic and ancestry-specific blood-cell genetics in 746,667 individuals from 5 global populations. Cell 182, 1198-1213. e1114.

Efron, B., 2007. Size, power and false discovery rates. The Annals of Statistics 35, 1351-1377.

Efron, B., 2010. Large-scale inference: empirical Bayes methods for estimation, testing, and prediction. Cambridge University Press.

Efron, B., Tibshirani, R., 2002. Empirical Bayes methods and false discovery rates for microarrays. Genetic epidemiology 23, 70-86.

Frei, O., Holland, D., Smeland, O.B., Shadrin, A.A., Fan, C.C., Maeland, S., O’Connell, K.S., Wang, Y., Djurovic, S., Thompson, W.K., 2019. Bivariate causal mixture model quantifies polygenic overlap between complex traits beyond genetic correlation. Nature communications 10, 2417.

Ghoussaini, M., Mountjoy, E., Carmona, M., Peat, G., Schmidt, E.M., Hercules, A., Fumis, L., Miranda, A., Carvalho-Silva, D., Buniello, A., 2021. Open Targets Genetics: systematic identification of trait-associated genes using large-scale genetics and functional genomics. Nucleic acids research 49, D1311-D1320.

GTEx Consortium 2017. Genetic effects on gene expression across human tissues ,. Nature 550, 204-213.

Holland, D., Frei, O., Desikan, R., Fan, C.-C., Shadrin, A.A., Smeland, O.B., Sundar, V.S., Thompson, P., Andreassen, O.A., Dale, A.M., 2020. Beyond SNP heritability: Polygenicity and discoverability of phenotypes estimated with a univariate Gaussian mixture model. PLoS Genetics 16, e1008612.

Howard, D.M., Adams, M.J., Clarke, T.-K., Hafferty, J.D., Gibson, J., Shirali, M., Coleman, J.R., Hagenaars, S.P., Ward, J., Wigmore, E.M., 2019. Genome-wide meta-analysis of depression identifies 102 independent variants and highlights the importance of the prefrontal brain regions. Nature neuroscience 22, 343-352.

Hyde, C.L., Nagle, M.W., Tian, C., Chen, X., Paciga, S.A., Wendland, J.R., Tung, J.Y., Hinds, D.A., Perlis, R.H., Winslow, A.R., 2016. Identification of 15 genetic loci associated with risk of major depression in individuals of European descent. Nature genetics 48, 1031-1036.

Karadag, N., Shadrin, A.A., O’Connell, K.S., Hindley, G.F., Rahman, Z., Parker, N., Bahrami, S., Fominykh, V., Cheng, W., Holen, B., 2023. Identification of novel genomic risk loci shared between common epilepsies and psychiatric disorders. Brain 146, 3392-3403.

Kircher, M., Witten, D.M., Jain, P., O'roak, B.J., Cooper, G.M., Shendure, J., 2014. A general framework for estimating the relative pathogenicity of human genetic variants. Nature genetics 46, 310-315.

Kundaje, A., Meuleman, W., Ernst, J., Bilenky, M., Yen, A., Heravi-Moussavi, A., Kheradpour, P., Zhang, Z., Wang, J., 2015. Integrative analysis of 111 reference human epigenomes. Nature 518, 317-330.

Levey, D.F., Stein, M.B., Wendt, F.R., Pathak, G.A., Zhou, H., Aslan, M., Quaden, R., Harrington, K.M., Nuñez, Y.Z., Overstreet, C., 2021. Bi-ancestral depression GWAS in the Million Veteran Program and meta-analysis in> 1.2 million individuals highlight new therapeutic directions. Nature neuroscience 24, 954-963.

Nichols, T., Brett, M., Andersson, J., Wager, T., Poline, J.-B., 2005. Valid conjunction inference with the minimum statistic. Neuroimage 25, 653-660.

Roadmap Epigenomics Consortium 2015. Integrative analysis of 111 reference human epigenomes. Nature 518, 317-330.

Schweder, T., Spjøtvoll, E., 1982. Plots of p-values to evaluate many tests simultaneously. Biometrika 69, 493-502.

Smeland, O.B., Frei, O., Shadrin, A., O’Connell, K., Fan, C.-C., Bahrami, S., Holland, D., Djurovic, S., Thompson, W.K., Dale, A.M., 2020. Discovery of shared genomic loci using the conditional false discovery rate approach. Human genetics 139, 85-94.

Sun, B.B., Maranville, J.C., Peters, J.E., Stacey, D., Staley, J.R., Blackshaw, J., Burgess, S., Jiang, T., Paige, E., Surendran, P., 2018. Genomic atlas of the human plasma proteome. Nature 558, 73-79.

Sun, L., Craiu, R.V., Paterson, A.D., Bull, S.B., 2006. Stratified false discovery control for large‐scale hypothesis testing with application to genome‐wide association studies. Genetic Epidemiology: The Official Publication of the International Genetic Epidemiology Society 30, 519-530.

Watanabe, K., Taskesen, E., Van Bochoven, A., Posthuma, D., 2017. Functional mapping and annotation of genetic associations with FUMA. Nature communications 8, 1826.

Wiström, E.D., O'Connell, K.S., Karadag, N., Bahrami, S., Hindley, G.F., Lin, A., Cheng, W., Steen, N.E., Shadrin, A., Frei, O., 2022. Genome‐wide analysis reveals genetic overlap between alcohol use behaviours, schizophrenia and bipolar disorder and identifies novel shared risk loci. Addiction 117, 600-610.

Yoo, Y.J., Pinnaduwage, D., Waggott, D., Bull, S.B., Sun, L., 2009. Genome-wide association analyses of North American Rheumatoid Arthritis Consortium and Framingham Heart Study data utilizing genome-wide linkage results, BMC proceedings. Springer, pp. 1-5.

Zhu, Z., Zhang, F., Hu, H., Bakshi, A., Robinson, M.R., Powell, J.E., Montgomery, G.W., Goddard, M.E., Wray, N.R., Visscher, P.M., 2016. Integration of summary data from GWAS and eQTL studies predicts complex trait gene targets. Nature genetics 48, 481-487.
